# Proteomics in Diagnostic Evaluation and Treatment of Breast Cancer: A Scoping Review

**DOI:** 10.3390/jpm15050177

**Published:** 2025-04-27

**Authors:** Menelaos Zafrakas, Ioannis Gavalas, Panayiota Papasozomenou, Christos Emmanouilides, Maria Chatzidimitriou

**Affiliations:** 1 School of Health Science, International Hellenic University, 57400 Thessaloniki, Greece; ppapasoz@ihu.gr (P.P.); chdimitr@ihu.gr (M.C.); 2 European Interbalkan Medical Center, Department of Medical Oncology, 55535 Thessaloniki, Greece; igava@auth.gr (I.G.); chrem@interbalkan-hosp.gr (C.E.)

**Keywords:** proteomics, proteomic, breast cancer, scoping review, patient subgroups

## Abstract

**Objectives:** The aim of this scoping review was to delineate the current role and possible applications of proteomics in personalized breast cancer diagnostic evaluation and treatment. **Methods:** A comprehensive search in PubMed/MEDLINE and Scopus/EMBASE was conducted, according to the PRISMA–ScR guidelines. Inclusion criteria: proteomic studies of specimens from breast cancer patients, clinically relevant studies and clinical studies. Exclusion criteria: in silico, in vitro and studies in animal models, review articles, case reports, case series, comments, editorials, and articles in language other than English. The study protocol was registered in the Open Science Framework. **Results:** In total, 1093 records were identified, 170 papers were retrieved and 140 studies were selected for data extraction. Data analysis and synthesis of evidence showed that most proteomic analyses were conducted in breast tumor specimens (n = 77), followed by blood samples (n = 48), and less frequently in other biologic material taken from breast cancer patients (n = 19). The most commonly used methods were liquid chromatography–tandem mass spectrometry (LC–MS/MS), followed by Matrix-assisted laser desorption/ionization-time of flight (MALDI–TOF), Surface-Enhanced Laser Desorption/Ionization Time-of-Flight (SELDI–TOF) and Reverse Phase Protein Arrays (RPPA). **Conclusions:** The present review provides a thorough map of the published literature reporting clinically relevant results yielded from proteomic studies in various biological samples from different subgroups of breast cancer patients. This analysis shows that, although proteomic methods are not currently used in everyday practice to guide clinical decision-making, nevertheless numerous proteins identified by proteomics could be used as biomarkers for personalized diagnostic evaluation and treatment of breast cancer patients.

## 1. Introduction

Breast cancer is the most common malignancy in women worldwide, ranking first in 157 countries, and it is the leading cause of death from cancer in 112 countries [1]. Incidence rates are higher in transitioned countries as compared with transitioning countries (54.1 vs. 30.8 per 100,000), whereas mortality rates are lower in the former (11.3 vs. 15.3 per 100,000, respectively) [1]. In the USA, breast cancer alone accounts for 32% of new cancer diagnoses [2]; approximately one in eight women, or 12.5%, will be diagnosed with invasive breast cancer, and one in forty-three, or 2.3%, will die from the disease [3]. In many high-income countries, mortality rates have decreased since the early 1990s, following new developments in early detection by screening, and progress in treatment [1,4].

Since the dawn of the 21st century, intensive research in gene expression analysis has led to the identification of different multi-gene signatures and the characterization of four subtypes of breast cancer, luminal A, luminal B, ERBB-B2-overexpressing and basal-like, and their respective clinico-pathological surrogate definitions, based on immunohistochemistry, i.e., luminal A-like, luminal B-like, HER2 positive (non-luminal) and triple negative (ductal) [5,6,7]. In recent years, several multi-gene expression assays, have been increasingly used in clinical practice, playing a pivotal role in personalized decision-making on whether to administer, or not, chemotherapy to patients with early luminal breast cancer; these assays include the cDNA-microarray-based 70-gene assay (MammaPrint), and the RT–PCR-based methods 21-gene assay (Oncotype DX), 50-gene assay (PAM50), and 12-gene assay (Endopredict) [8,9]. Modern high-throughput proteomic analysis methods have emerged at the end of the last century [10,11], and since then they have been widely used in breast cancer research [12,13]. Still, in contrast to genomic analyses, it is not clear to clinicians whether proteomic-based methods have any clinical applications in breast cancer diagnosis and therapy. Hence, the aim of the present scoping review was to investigate and delineate the current role and possible emerging clinical applications of proteomic analyses in personalized diagnostic evaluation and treatment of breast cancer patients.

## 2. Materials and Methods

The present study was conducted according to the Preferred Reporting Items for Systematic Reviews and Meta-analyses extension for Scoping Reviews (PRISMA–ScR) [14]; the PRISMA–ScR Checklist is presented in Supplementary Table S1. The study protocol was registered in the Open Science Framework (registration DOI: https://doi.org/10.17605/OSF.IO/BE3WF, accessed on 23 March 2025).

### 2.1. Eligibility Criteria

The PCC (i.e., Population, Concept, Context) criteria [14,15] were used as follows: (1) Population: Breast cancer patients. (2) Concept: Proteomic(s) analyses applied in clinical and clinically relevant research in breast cancer. (3) Context: Breast cancer and normal breast tissue, lymph nodes, blood (serum and/or plasma) and any other biological material taken from breast cancer patients; in vitro studies in breast cancer cell lines would be excluded.

The following inclusion criteria were used: (1) Studies with proteomic analyses of specimens taken from breast cancer patients, i.e., primary and metastatic lesions, normal breast tissue, lymph nodes, blood (serum and plasma), other biological material; (2) clinically relevant studies for diagnostic and therapeutic target discovery and validation; (3) clinical studies. The following exclusion criteria were used: (1) in silico studies; (2) in vitro studies in breast cancer cell lines; (3) studies in animal models; (4) review articles; (5) case reports, case series and, in general, studies with a number of patient specimens lower than 10; (6) comments and editorials; (7) articles in language other than English.

### 2.2. Information Sources and Search

The present scoping review was conducted by searching the two primary online medical databases: the PubMed/MEDLINE database (PubMed) and the EMBASE database (Scopus) for articles published until 23 March 2025. The following search terms were used in both database searches: “proteomic” AND “breast cancer”, and “proteomics” AND “breast cancer”, all in title.

### 2.3. Selection of Sources of Evidence

For study selection, two authors (IG and PP) screened independently each study in order to exclude overlapping and duplicate publications, as well as studies not published in English, and then assessed the titles and/or abstracts of the remaining records according to the inclusion and exclusion criteria; after retrieval of eligible studies, the full-texts were further assessed according to the inclusion and exclusion criteria; any conflicts were resolved by discussion or referral to a third author (MZ), who also re-assessed and confirmed the inclusion of eligible publications.

### 2.4. Data Charting Process, Data Items, and Synthesis of Results

Data collection was carried out by two independent reviewers (IG and PP), by using Microsoft Excel filled out with data taken from the main text, the figures, and the tables of the included studies; any conflicts were resolved by discussion or referral to a third author (MZ), who also re-assessed and confirmed collection of data. Furthermore, two separate lists of excluded studies were constructed, i.e., in vitro and in silico studies.

The following data items were extracted from the included articles: (1) first author; (2) year of publication; (3) country of first author’s affiliation; (4) clinical setting, i.e., whether the possible clinical application of the study was diagnostic or relevant to treatment; (5) disease stage, i.e., ductal carcinoma in situ (stage 0), primary invasive breast cancer (stages I, II and III), and metastatic breast cancer (stage IV); (6) type of specimen analyzed; (7) number of specimens analyzed; (8) the primary proteomic method used; (9) other methods used; (10) main conclusions. Data items were not extracted from the excluded articles. Extracted data from included studies were further analyzed according to clinical relevance, i.e., according to (a) the diagnostic or therapeutic setting, (b) the type of tissue specimen analyzed, and (c) the disease stage.

## 3. Results

### 3.1. Search Results

**Figure 1:**
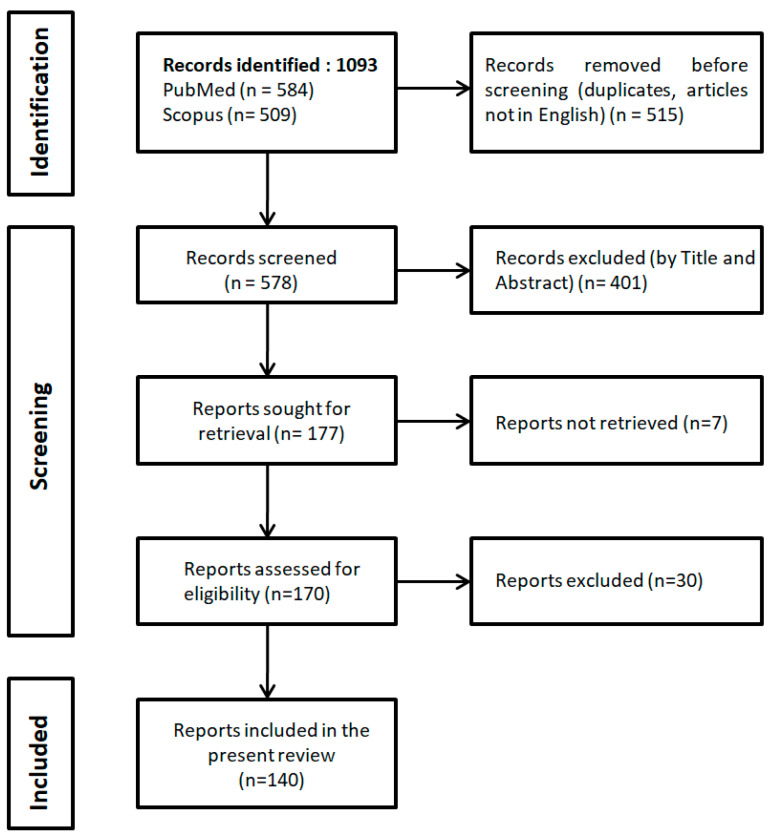
Preferred Reporting Items for Systematic Reviews and Meta-analyses extension for Scoping Reviews (PRISMA–ScR) flowchart.

The study selection process and results are presented diagrammatically as a flow chart in Figure 1, according to the Preferred Reporting Items for Systematic Reviews and Meta-analyses extension for Scoping Reviews (PRISMA–ScR) [14]. In brief, 1093 studies (584 in PubMed and 509 in Scopus) were identified by using the search terms “proteomic” AND “breast cancer”, and “proteomics” AND “breast cancer”, all in title. After removal of duplicates, overlapping and studies not published in English (n = 515), the titles and/or abstracts of 578 records were screened and 401 articles were excluded, leaving 177 articles to be sought for retrieval. Next, 170 articles were retrieved and, after assessment of their full-text, 30 reports were excluded according to the eligibility criteria. Finally, 140 articles were included [16,17,18,19,20,21,22,23,24,25,26,27,28,29,30,31,32,33,34,35,36,37,38,39,40,41,42,43,44,45,46,47,48,49,50,51,52,53,54,55,56,57,58,59,60,61,62,63,64,65,66,67,68,69,70,71,72,73,74,75,76,77,78,79,80,81,82,83,84,85,86,87,88,89,90,91,92,93,94,95,96,97,98,99,100,101,102,103,104,105,106,107,108,109,110,111,112,113,114,115,116,117,118,119,120,121,122,123,124,125,126,127,128,129,130,131,132,133,134,135,136,137,138,139,140,141,142,143,144,145,146,147,148,149,150,151,152,153,154,155], and data extraction followed. Two hundred and ninety eight excluded studies, i.e., 243 in vitro studies, 23 in silico studies and 32 reviews, are presented in Supplementary File S1–S3, respectively.

### 3.2. Year of Publication and Geographical Area of Included Studies

Regarding the year of publication, the number of studies appeared to increase over time. In detail, six studies were published between 2001 and 2004 [82,83,104,112,113,118], 22 studies between 2005 and 2009 [26,46,48,61,68,72,80,84,96,97,98,99,100,101,105,109,116,119,126,128,146,147], 30 studies between 2010 and 2014 [25,31,32,36,37,38,39,40,42,43,49,57,58,59,62,63,69,78,90,92,94,102,111,115,124,140,141,148,150,151], 38 studies between 2015 and 2019 [16,18,28,29,33,34,35,41,45,51,52,53,54,55,66,70,71,76,81,88,93,95,106,107,108,110,114,127,131,132,135,138,139,142,144,145,149,153] and 44 studies were published between 2020 and March 23, 2025 [17,19,20,21,22,23,24,27,30,44,47,50,56,60,64,65,67,73,74,75,77,79,85,86,87,89,91,103,117,120,121,122,123,125,129,130,133,134,136,137,143,152,154,155] (see Supplementary Figure S1).

Regarding the country of first author’s affiliation (a) 47 studies came from Europe, i.e., nine from Italy [26,37,38,45,57,58,96,109,110], eight from Germany [29,31,85,88,98,99,114,121], six from Spain [42,53,54,55,56,114], five from France [32,43,61,66,132], five from the Netherlands [34,44,59,92,94], four from Sweden [64,67,77,100], three from Denmark [36,65,129], two from the UK [95,144], two from the Czech Republic [33,108], two from Russia [125,130], and one from Norway [30]; (b) 46 studies came from Asia, i.e., 18 from China [21,47,72,85,87,113,121,134,135,136,137,138,139,140,143,145,152,153], six from South Korea [76,79,81,82,122,141], six from India [51,52,60,103,127,154], three from Israel [107,120,142], three from Singapore [101,149,150], two from Japan [90,128], two from Malaysia [20,74], two from Thailand [40,75], one from Iran [91], one from Saudi Arabia [16], one from Turkey [18], and one from Taiwan [155]; (c) 45 studies came from North and South America, i.e., 35 from the USA [17,19,25,27,46,48,50,62,63,68,69,70,71,73,78,80,83,84,89,93,97,104,105,111,116,118,119,123,124,126,133,146,147,148,151], seven from Brazil [23,24,35,41,102,106,117], two from Canada [22,39], and one from Mexico [49]; and (d) two studies came from Australia [28,112] (Supplementary Figure S2). It is noteworthy that, in 17 studies, the authors came from more than one country [16,26,33,42,50,55,59,63,65,84,88,94,116,123,126,131,133].

### 3.3. Clinical Application, Disease Stage and Specimen Type of Included Studies

Regarding the possible clinical application of each study 105 studies were relevant to diagnosis [16,18,20,21,22,23,24,25,26,27,28,29,30,31,33,34,35,37,38,39,40,41,45,46,49,50,51,52,53,54,57,58,59,60,61,64,67,70,71,72,73,74,75,79,80,81,82,83,84,85,88,89,93,94,95,96,97,98,99,101,102,103,104,105,107,108,109,110,111,112,113,114,115,116,117,118,119,122,123,125,127,128,129,130,131,132,133,134,135,136,139,140,142,143,144,145,146,147,148,149,150,151,152,153,154], 25 were relevant to treatment [17,19,32,36,43,44,47,56,62,65,66,76,77,86,90,91,92,106,120,121,124,137,138,141,155] and 10 were relevant to both diagnosis and treatment [42,48,55,63,68,69,78,87,100,126] (Supplementary Figure S3).

Regarding the disease stage, specimens taken from patients with primary invasive breast cancer (stages I, II and III) were examined in 135 studies (in 114 studies primary invasive alone [16,18,19,20,21,22,23,24,26,27,29,30,31,32,33,34,35,36,37,38,39,40,41,42,44,45,46,47,49,50,51,52,53,54,55,56,57,58,59,61,62,64,65,66,67,68,69,70,74,75,76,77,78,81,82,83,85,86,87,88,89,91,93,94,95,96,97,98,99,100,101,103,104,105,106,107,108,109,110,112,114,115,116,117,118,120,121,123,124,126,127,128,129,130,131,133,134,135,136,137,138,139,140,142,144,145,146,148,149,150,151,152,153,155], in 10 studies together with metastatic breast cancer [48,72,80,84,102,113,122,141,143,154], in eight studies together with DCIS [28,71,73,79,90,111,132,147], in two studies together with DCIS and metastatic [63,119], and in one study together with DCIS and recurrent breast cancer [25]), while specimens taken from patients with metastatic breast cancer were examined in seventeen studies (in five metastatic alone [17,43,60,92,125], in ten together with primary invasive breast cancer [48,72,80,84,102,113,122,141,143,154] and in two together with primary invasive breast cancer and DCIS [63,119]) (Supplementary Figure S4).

Regarding the type of specimen analyzed, breast cancer tissue was analyzed in 77 studies (Table 1), blood samples were analyzed in 48 studies (in 33 studies in serum and in 15 studies in plasma) (Table 2), and other biologic materials, i.e., nipple aspiration fluid (NAF), urine, saliva, tear fluid, pleural effusions, tumor interstitial fluid, and lymph nodes, were analyzed in 19 studies (Table 3). 

#### 3.3.1. Proteomic Studies in Breast Cancer Tissue

For each proteomic study in breast cancer tissue, the number of specimens analyzed, the primary proteomic method used, other methods used if any and the main findings of each study are presented in Table 1. In brief, 52 studies were relevant to breast cancer diagnostic evaluation [16,18,20,22,23,24,25,29,30,33,34,37,38,39,40,45,46,49,53,54,67,73,74,79,82,93,94,96,97,98,99,101,102,107,108,109,110,112,113,114,116,122,128,131,132,139,140,142,145,149,150,153], 18 were relevant to breast cancer therapy [17,32,36,44,47,56,62,65,66,76,77,90,120,121,124,138,140,155], and 8 studies were relevant to both breast cancer diagnostic evaluation and treatment [42,55,63,68,69,87,100,126].

In sixty-seven studies proteomic analysis was conducted in primary invasive breast tumors only [16,18,20,22,23,24,29,30,32,33,34,36,37,38,39,40,42,44,45,46,47,49,53,54,55,56,62,65,66,67,68,69,74,76,77,82,87,93,94,96,97,98,99,100,101,107,108,109,110,112,114,116,120,121,124,126,128,131,138,139,140,142,145,149,150,153,155], in four studies in both primary invasive breast tumors and ductal carcinoma in situ (DCIS) [73,79,90,132], in one study in primary invasive, locally recurrent and DCIS [25], in one study in primary invasive, metastatic and DCIS [63], in four studies in both primary and metastatic lesions [102,113,122,141], and in one study only in breast cancer metastases [17]. The number of breast cancer specimens analyzed ranged between 10 and 990 (median = 60). The most common proteomic method used was liquid chromatography–tandem mass spectrometry (LC–MS/MS), followed by Matrix-assisted laser desorption/ionization-time of flight (MALDI–TOF) and Reverse Phase Protein Arrays (RPPA). The most common additional methods used were immunohistochemistry and Western blot.

Regarding the main findings, 29 studies identified possible biomarkers for breast cancer diagnosis, prognosis, staging and disease progression [17,18,20,22,24,25,29,33,37,38,39,45,46,54,73,82,97,102,107,108,109,113,116,122,128,132,145,149,153], 17 studies identified possible biomarkers for specific breast cancer subtypes [23,34,42,49,54,55,68,76,87,94,96,98,114,131,139,142,150], 9 studies identified possible biomarkers for breast cancer diagnosis and pathogenesis [16,30,40,67,74,79,101,110,112], 16 studies identified possible biomarkers for response to therapy [32,44,47,63,65,66,69,77,93,99,100,120,124,138,140,141], and 7 studies identified possible therapeutic targets [36,56,62,90,121,126,155]. More details regarding the main findings of each study are presented in Supplementary Table S2.

#### 3.3.2. Proteomic Studies in Plasma and Serum from Breast Cancer Patients

An overview of proteomic studies in plasma and serum from breast cancer patients is presented in Table 2, showing the type and number of specimens analyzed, the primary proteomic method used, other methods used if any and the main findings of each study. In brief, serum was analyzed in 32 studies [26,27,43,48,50,51,57,58,59,61,70,71,72,79,83,87,88,90,94,111,113,119,123,125,134,135,136,137,144,148,149,154], and plasma was analyzed in 15 studies [19,21,41,64,77,81,85,106,117,127,130,133,143,150,152]. Of these, 39 studies were relevant to breast cancer diagnostic evaluation [21,26,27,41,50,51,57,58,59,61,64,70,71,72,79,81,83,87,88,94,111,113,117,119,123,125,127,130,133,134,135,136,143,144,148,149,150,152,154], 6 studies were relevant to breast cancer treatment [19,43,85,90,106,137], and 2 studies were relevant to both breast cancer diagnostic evaluation and treatment [48,77].

In 35, studies proteomic analysis was conducted in patients with primary invasive breast tumors (stage I-III) only [19,21,26,27,41,50,51,57,58,59,61,64,77,81,83,85,87,88,90,94,106,113,117,123,127,130,133,134,135,136,137,144,149,150,152], in 4 studies in patients with both primary invasive breast tumors and ductal carcinoma in situ (DCIS) [70,71,111,148], in 1 study in patients with primary invasive, metastatic and DCIS [119], in 5 studies in patients with primary and patients with metastatic disease [48,72,79,143,154], and in 2 studies only in patients with breast cancer metastases [43,125]. The number of breast cancer specimens analyzed ranged between 10 and 796 (median = 76). The most common proteomic method used was liquid chromatography–tandem mass spectrometry (LC–MS/MS), followed by Surface-Enhanced Laser Desorption/Ionization Time-of-Flight (SELDI–TOF) and Matrix-assisted laser desorption/ionization-time of flight (MALDI–TOF). The most common additional method used was Western blot.

Regarding the main findings, 14 studies identified possible biomarkers for early diagnosis of breast cancer [26,50,70,71,72,81,83,88,123,133,134,148,149,150], 16 studies identified possible biomarkers for diagnosis, prognosis, staging and disease progression [21,27,58,59,61,64,79,111,113,119,125,130,136,143,144,154], 6 studies identified possible biomarkers for specific breast cancer subtypes [41,51,57,117,127,152], 8 studies identified possible biomarkers for response to therapy [19,43,48,85,90,106,135,137], 1 study identified possible diagnostic and therapeutic targets [77], 1 study identified possible biomarkers for cancer related fatigue syndrome [94], and 1 study identified possible biomarkers for neuropathic pain [87]. More details regarding the main findings of each study are presented in Supplementary Table S3.

#### 3.3.3. Proteomic Studies in Other Biologic Material from Breast Cancer Patients

An overview of proteomic studies in biologic material from breast cancer patients other than breast cancer tissue, plasma and serum, i.e., nipple aspiration fluid, urine, saliva, tear fluid, pleural effusions, tumor interstitial fluid and lymph nodes, is given in Table 3, which presents the type and number of specimens analyzed, the primary proteomic method used, other methods used if any, and the main findings of each study. In brief, proteomic studies were conducted in nipple aspiration fluid (NAF) in five studies [35,82,104,105,118], in urine in three studies [28,52,74], in saliva in three studies [60,123,151], in tear fluid in two studies [25,50], in pleural effusions in one study [91], in tumor interstitial fluid in one study [129], and in lymph nodes in four studies [103,107,145,153]; eighteen studies were relevant to breast cancer diagnostic evaluation [25,28,35,50,52,60,74,82,103,104,105,107,118,123,129,145,151,153], and only one study was relevant to breast cancer therapy [91]. The number of breast cancer specimens analyzed ranged between 10 and 54 (median = 25). The most common proteomic method used was liquid chromatography–tandem mass spectrometry (LC–MS/MS), followed by Surface-Enhanced Laser Desorption/Ionization Time-of-Flight (SELDI–TOF) and Isobaric Tag for Relative and Absolute Quantification (iTRAQ). The most common additional method used was Western blot. Regarding the main findings, 12 studies identified possible biomarkers for differentiating breast cancer patients from healthy individuals [28,31,35,52,60,74,84,104,105,118,123,151], four studies identified possible biomarkers for nodal status [82,103,145,153], one study identified possible biomarkers for specific breast cancer subtypes [129], and one study identified possible biomarkers for response to therapy [91]. More details regarding the main findings of each study are presented in Supplementary Table S4.

### 3.4. Possible Clinical Use of Biomarkers Identified by the Included Studies

Studies with at least 30 specimens from breast cancer patients analyzed were further categorized according to their possible clinical use, in studies that identified biomarkers for breast cancer prognosis (Table 4), early diagnosis (Table 5), characterization of breast cancer subtypes (Table 6), and biomarkers for response to treatment (Table 7).

In Table 4, Table 5, Table 6 and Table 7 studies are presented in descending order of the number of specimens analyzed in each study, as this order reflects the impact of studies. Table 4 presents 11 studies that identified biomarkers for breast cancer prognosis; the number of specimens analyzed ranged between 60 and 990; these studies were carried out in primary tumor specimens (10 studies) or blood samples (1 study) from patients with non-metastatic invasive breast cancer (stages I-III). Table 5 presents 13 studies that identified biomarkers for early diagnosis of breast cancer; the number of specimens from breast cancer patients analyzed ranged between 30 and 796; it is noteworthy that all 13 studies were conducted in blood samples (serum or plasma) from patients with stage 0-IV disease. Table 6 shows 17 studies that identified biomarkers for characterization of breast cancer subtypes; the number of specimens analyzed ranged between 32 and 429; these studies were carried out either in primary tumor specimens (12 studies) or blood samples (5 studies) from patients with invasive non-metastatic breast cancer (stages I-III). Finally, Table 7 presents 21 studies that identified biomarkers for response to treatment; the number of specimens analyzed was between 36 and 880; these studies were carried out in primary tumor specimens (16 studies), blood samples (4 studies), and pleural effusions (1 study) from patients with stage 0-IV disease.

### 3.5. Overview of the Most Important Findings from the Largest Studies and the Most Commonly Used Proteomic Platforms Across the Studies

Regarding prognosis and response to treatment, in the largest study to date, Cawthorn et al. [39] analyzed a tissue microarray consisting of 990 early breast cancer cases (590 stage I and 400 stage II) and found that high expression levels of Decorin and Endoplasmin (HSP90B1) were associated with increased metastasis-poorer survival and may guide the use of hormonal therapy. Gonzalez-Angulo et al. [48] analyzed 880 primary breast tumor specimens and 256 FNAs obtained from primary breast cancer and developed a 10-protein biomarker panel that classifies breast cancer into prognostic subgroups, predicting relapse-free survival (RFS) and pathologic Complete Response (pCR) to neoadjuvant therapy. Bernhardt et al. [29] analyzed breast cancer specimens from 801 consecutive patients and identified SHMT2 and ASCT2 protein expression as novel potential prognostic biomarkers for breast cancer, as their high protein expression was associated with poor outcome.

Regarding early diagnosis, in the largest study to date, Grassmann et al. [64] anayzed plasma samples from 1577 women (796 incident breast cancer cases and 781 controls) participating in the prospective KARMA mammographic screening cohort and concluded that the levels of the studied plasma proteins were unlikely to offer additional benefits for risk prediction of short-term overall breast cancer risk, but could provide interesting insights into the biological basis of breast cancer in the future. Kim et al. [81] quantified three peptides, apolipoprotein C-1, carbonic anhydrase 1, and neural cell adhesion molecule L1-like protein in human plasma from 575 breast cancer patients and 454 healthy controls and concluded that these three petides can be a useful tool for breast cancer screening.

The most commonly used proteomic methods across the studies were mass spectrometry-based methods in 61 studies [4,5,6,7,8,9,10,13,19,20,21,25,26,32,34,35,37,38,39,40,51,52,53,59,60,61,62,63,65,72,82,88,91,92,93,95,97,103,106,107,108,111,114,115,116,117,118,119,121,122,125,126,127,128,129,130,131,133,134,138,139], most commonly liquid chromatography–tandem mass spectrometry (LC–MS/MS), followed by Matrix-assisted laser desorption/ionization-time of flight (MALDI–TOF), in 20 studies [1,16,22,23,30,64,67,70,77,85,86,87,96,101,102,105,123,135,136,137], Surface-Enhanced Laser Desorption/Ionization Time-of-Flight (SELDI–TOF) in 15 studies [11,17,28,42,43,44,46,54,57,69,71,81,83,90,104], Reverse Phase Protein Arrays (RPPA) in 11 studies [2,14,27,47,48,50,79,80,110,112,120], and Isobaric tag for absolute and relative quantification (iTRAQ) in 9 studies [18,24,36,73,89,109,113,132,140].

## 4. Discussion

Although proteomic analyses have been widely used in breast cancer research over the last two decades, it remains unclear if these efforts have been translated into direct clinical applications. The present scoping review is the first structured synthesis of evidence, delineating the current state and the possible future clinical applications of proteomic analyses in breast cancer diagnosis and treatment. To this end, we conducted a thorough search of the literature and found that, so far, the majority of pertinent proteomic analyses have been carried out in breast cancer cell lines rather than clinical specimens from breast cancer patients (see Supplementary File S1). After the search of the literature, study selection and data extraction followed and our data analysis and synthesis of results from 140 clinically relevant studies [16,17,18,19,20,21,22,23,24,25,26,27,28,29,30,31,32,33,34,35,36,37,38,39,40,41,42,43,44,45,46,47,48,49,50,51,52,53,54,55,56,57,58,59,60,61,62,63,64,65,66,67,68,69,70,71,72,73,74,75,76,77,78,79,80,81,82,83,84,85,86,87,88,89,90,91,92,93,94,95,96,97,98,99,100,101,102,103,104,105,106,107,108,109,110,111,112,113,114,115,116,117,118,119,120,121,122,123,124,125,126,127,128,129,130,131,132,133,134,135,136,137,138,139,140,141,142,143,144,145,146,147,148,149,150,151,152,153,154,155] showed that the number of publications regarding proteomic analyses in clinical specimens from breast cancer patients has been steadily increasing over time (see Supplementary Figure S1), and that three geographical areas, i.e., Europe, Asia, and the Americas, have an almost equal share in these publications (see Supplementary Figure S2). We also found that most clinically relevant studies have possible applications in diagnosis rather than therapy (see Supplementary Figure S3), and that most studies were carried out in specimens taken from patients with primary invasive breast cancer (stages I-III), rather than patients with metastatic disease (stage IV) or DCIS (stage 0) (see Supplementary Figure S4). Furthermore, our data analysis and synthesis of evidence showed that most proteomic analyses were conducted in breast tumor specimens, followed by plasma and serum, and only rarely in other biologic material taken from breast cancer patients. Overall, the most commonly used method in clinically relevant studies were liquid chromatography–tandem mass spectrometry (LC–MS/MS), followed by Matrix-assisted laser desorption/ionization-time of flight (MALDI–TOF), Reverse Phase Protein Arrays (RPPA) and Surface-Enhanced Laser Desorption/Ionization Time-of-Flight (SELDI–TOF) (see Table 1, Table 2 and Table 3).

In the present scoping review, neither a critical appraisal nor a risk of bias assessment across studies was conducted, since the former is not mandatory and the latter is not applicable in scoping reviews [14,15]. However, certain clinically relevant points should be highlighted. First, the wide-spread use of neoadjuvant chemotherapy in recent years has been a paradigm shift in breast cancer treatment, with new clinically critical questions arising, such as response to treatment, and residual disease after neoadjuvant therapy. However, studies with proteomic analyses of specimens from patients treated with neoadjuvant chemotherapy are still scarce [32,47,63,120,124,141]. Also hard to find are studies with proteomic analyses of specimens from patients diagnosed with triple negative tumors, the most aggressive form of breast cancer, with currently finite treatment options [36,55,56,60,65,85,86,114,121,124]. Likewise, proteomic analyses in specimens obtained from patients with metastatic disease are also scarce [17,43,48,63,72,79,102,113,119,122,125,141,143]. Intriguingly, though blood samples are theoretically more readily available than tumor tissue, our analysis showed that more studies have been published regarding proteomic analysis of breast tumor specimens than studies in blood taken from breast cancer patients (see Table 1 and Table 2). Even more rare are studies in lymph nodes, and surprisingly studies with proteomic analysis of urine from breast cancer patients (see Table 3). In recent years, the extent of axillary surgery has been steadily receding, due to the ever increasing number of screen-detected breast cancers at earlier stages, the wide-spread use of sentinel lymph node biopsy and, more recently, due to the use of targeted axillary dissection (TAD) following neoadjuvant chemotherapy [156,157]. With such novel developments, new clinical questions arise in respect to nodal status of breast cancer patients, with implications regarding patient diagnosis, staging, prognosis and treatment, directing proteomic and basic research in general to be focused on these issues.

Another noteworthy clinically relevant point is that, although different studies claim the identification of new therapeutic targets by using proteomic analysis, in fact these targets may not be all that new; for example, the MAGE genes were identified as potential therapeutic targets in breast cancer by RT–PCR gene expression analysis [158] many years before proteomic studies came to the same conclusion [36,121]. A final critical issue worthy to be addressed is that the number of specimens analyzed in proteomic studies is relatively low as compared with that of genomic studies. In particular, as shown in Table 1 and Table 4, the highest numbers of breast tumor specimens analyzed by proteomics in the included studies were 990 [39], 880 [62] and 801 [29] and the findings of these studies did not have any direct impact on clinical decision-making. In contrast, much higher numbers of breast tumor specimens have been analyzed in studies using multigene assays, such as the 70-gene assay (Mammaprint) (n = 6600) [159], the 21-gene assay (OncotypeDX) (n = 10,273) [160], the 50-gene assay (PAM50) (n = 2558) [161], and the 12-gene assay (Endopredict) (n = 2185) [162], and these genomic assays are used in everyday practice to guide clinical decision-making on whether or not to administer chemotherapy in early luminal A and B (estrogen-receptor-positive) breast cancer. This is not surprising, since historically the development of high-throughput transcriptomics preceded that of proteomics, and gene expression signatures with prognostic significance were already identified at the eve of the 21st century [5,6], well before the widespread use of proteomics. Hence, it seems that so far the relatively low number of specimens analyzed by proteomics has been a barrier to translation of findings into clinical practice. Besides small sample sizes, other such possible barriers may include lack of assay standardization, lack of reproducibility of results, costs, validation challenges, and diversity of proteomic methods applied. In the future, the investigational use of proteomic analyses in the translational arm of large, adequately powered, multicenter randomized clinical trials, with centralized laboratory facilities using standardized, reproducible and validated assays, might lead to the integration of proteomics in clinical practice, especially since many clinically relevant questions remain open. In particular, the aforementioned multigene assays (Mammaprint, OncotypeDX, PAM50 and Endopredict) provide prognostic and in part predictive information in a subset of breast cancer patients with early disease (hormone receptor positive, HER2-negative tumors, up to 5 cm in maximal diameter, with up to three positive lymph nodes), while no such robust evidence-based information is available for more advanced breast cancer stages, neither with transcriptomic nor with proteomic approaches. Therefore, proteomic analyses may be used for the identification of biomarkers providing prognostic information, especially in advanced breast cancer stages, as well as for the identification of biomarkers for other applications, such as early detection, response to treatment and resistance to treatment.

The main strength of the present study is its originality, as this is the first scoping review on this topic. Most of the other published articles reviewing the role of proteomics in breast cancer research are narrative reviews and there is a paucity of relevant systematic reviews (see Supplementary File S3). Hence, it is possible that the present study may lead to primary studies and systematic reviews regarding applications of proteomics in identifying biomarkers for early diagnosis of breast cancer, characterization of breast cancer subtypes, potential therapeutic targets, and specific biomarkers for response to treatment. Another strong point of this review is that we have focused on proteomic analyses of clinical specimens taken from patients with breast cancer, by excluding in vitro and in silico studies. On the other hand, a weakness of this study is that both the variety of proteomic methods used and the diversity of clinical specimens analyzed hamper comparisons between studies and data synthesis leading to clinically applicable conclusions. Nevertheless, it is the vast heterogeneity of breast cancer at the molecular, histopathological and clinical level that necessitates this multiplicity of methodological approaches.

## 5. Conclusions

This is the first methodologically structured review investigating the current state of proteomic applications in breast cancer diagnostic evaluation and treatment. Given the variety of available proteomic methods and the vast heterogeneity of breast cancer at the molecular, histopathological and clinical level, conducting a scoping review was deemed the most appropriate methodological approach. Our data analysis and synthesis of evidence showed a relatively low number of studies in blood and a paucity of studies in specimens such as lymph nodes and urine obtained from breast cancer patients. Furthermore, the number of studies with proteomic analyses focusing on certain breast cancer subtypes, especially on triple negative tumors, and on monitoring response to treatment in the neoadjuvant and metastatic setting is relatively low. Currently, in contrast to transcriptomics, proteomic analyses are not used in everyday practice to guide clinical decision-making. On the other hand, multiple proteomic studies yielded clinically relevant results, which hold the promise of translating these findings to routine clinical applications. Hence, it seems that there are multiple perspectives for proteomic studies in breast cancer research in the future. The insights provided by the present study can be used to develop new approaches in research and novel strategies in personalized diagnostic evaluation and treatment of breast cancer, and other tumor entities.

## Figures and Tables

**Table 1:** Proteomic studies in tumor tissue specimens from breast cancer patients.

Study	Country	Setting	BC Stage	Number of BC Specimens	Primary Proteomic Method	Additional Laboratory Methods	Main Findings
Abdullah et al., 2016 [16]	Saudi Arabia	Diagnosis	Primary	20	MALDI–TOF	n.a.	Biomarkers for diagnosis and pathogenesis
Akcakanat et al., 2021 [17]	USA	Therapy	Metastatic	37	RPPA	DNA and RNA sequencing	Biomarkers differentiating primary from metastatic BC
Akpinar et al., 2017 [18]	Turkey	Diagnosis	Primary	10	2D–PAGE	n.a.	Biomarkers differentiating primary from metastatic BC
Al-Wajeeh et al., 2020 [20]	Malaysia	Diagnosis	Primary	80	SDS–PAGE andLC–MS/MS	n.a.	Biomarkers for staging
Asleh et al., 2022 [22]	Canada	Diagnosis	Primary	300	LC–MS/MS-based proteomics	IHC	Biomarkers for diagnosis and prognosis
Azevedo et al., 2023 [23]	Brazil	Diagnosis	Primary	19	High-throughput MS	IHC	Biomarkers for BC subtypes
Azevedo et al., 2022 [24]	Brazil	Diagnosis	Primary	19	LC–MS/MS	In silico transcriptomic analysis	Biomarkers for diagnosis and prognosis
Bateman et al., 2010 [25]	USA	Diagnosis	P, R, DCIS	25	LC–MS/MS	IHC	Biomarkers for disease progression and recurrence
Bernhardt et al., 2017 [29]	Germany	Diagnosis	Primary	801	RPPA	IHC	Biomarkers for prognosis
Bjørnstad et al., 2024 [30]	Norway	Diagnosis	Primary	107	MS	Ms in vitro	Biomarkers for diagnosis and pathogenesis
Bonneterre et al., 2013 [32]	France	Therapy	Primary	149	SELDI–TOF MS	n.a.	Biomarkers for response to therapy
Bouchal et al., 2015 [33]	Czech Republic	Diagnosis	Primary	160	iTRAQ-based proteomics	IHC, transcriptomics	Biomarkers for staging and nodal status
Braakman et al., 2015 [34]	Nedetherlands	Diagnosis	Primary	11	nano-LC–MS/MS	DNA analysis	Biomarkers for BC subtypes
Cabezón et al., 2012 [36]	Denmark	Therapy	Primary	78	2D-PAGE and MS	2D Western Immunoblotting, IHC	Biomarkers for therapy
Cancemi et al., 2012 [37]	Italy	Diagnosis	Primary	100	MALDI–TOF MS	IHC, Western blot	Biomarkers for disease progression
Cancemi et al., 2010 [38]	Italy	Diagnosis	Primary	100	MALDI–TOF MS	Western blot, n terminal microsequencing	Biomarkers for prognosis
Cawthorn et al., 2012 [39]	Canada	Diagnosis	Primary	990	iTRAQ and LC–MS/MS	IHC, SRM–MS	Biomarkers for prognosis
Champattanachai et al., 2013 [40]	Thailand	Diagnosis	Primary	26	LC–MS/MS	in vitro assays	Biomarkers for diagnosis and pathogenesis
Creighton et al., 2010 [42]	Spain	D and T	Primary	429	RPPA	In vitro assays and quantitative real-time PCR	Biomarkers for BC subtypes
Debets et al., 2023 [44]	Netherlands	Therapy	Primary	45	Phosphoproteomics	n.a.	Biomarkers for response to therapy
Di Cara et al., 2019 [45]	Italy	Diagnosis	Primary	80	MALDI–TOF MS	n.a.	Biomarkers for disease progression and prognosis
Duan et al., 2023 [47]	China	Therapy	Primary	139	MS	n.a.	Biomarkers for response to therapy
Fonseca-Sánchez et al., 2012 [49]	Mexico	Diagnosis	Primary	105	LC/ESI-MS/MS	IHC, Western blot	Biomarkers for BC subtypes
Gámez-Pozo et al., 2017 [53]	Spain	Diagnosis	Primary	106	LC–MS	MicroRNA expression analysis	Biomarkers for BC subtypes
Gámez-Pozo et al., 2017 [54]	Spain	Diagnosis	Primary	60	LC–MS	Parallel reaction monitoring	Biomarkers for prognosis
Gámez-Pozo et al., 2015 [55]	Spain	D and T	Primary	96	LC–MS/MS	MicroRNA expression and in vitro assays	Biomarkers for BC subtypes
García-Adrián et al., 2021 [56]	Spain	Therapy	Primary	125	MS	n.a.	Biomarkers for therapy
Gonzalez-Angulo et al., 2013 [62]	USA	Therapy	Primary	175	RPPA	IHC	Biomarkers for therapy
Gonzalez-Angulo et al., 2011 [63]	USA	D and T	P, M, DCIS	880	RPPA	IHC	Biomarkers for prognosis and response to therapy
Gromova et al., 2021 [65]	Denmark	Therapy	Primary	44	RPPA	IHC and PCR	Biomarkers for response to therapy
Guerin et al., 2018 [66]	France	Therapy	Primary	46	MS RPLC	Western blot	Biomarkers for response to therapy
Gustafsson et al., 2024 [67]	Sweden	Diagnosis	Primary	63	LC–MS/MS	n.a.	Biomarkers for diagnosis and pathogenesis
He et al., 2011 [68]	USA	D and T	Primary	39	LC–MS	IHC	Biomarkers for BC subtypes and response to therapy
He et al., 2009 [69]	USA	D and T	Primary	52	SELDI–TOF MS	IHC	Biomarkers for response to therapy
Hulahan et al., 2024 [73]	USA	Diagnosis	P and DCIS	22	Multiplexed spatial proteomics	n.a.	Biomarkers differentiating invasive from DCIS
Izani Othman et al., 2009 [74]	Malaysia	Diagnosis	Primary	20	LC–MS/MS	Western blot, MS	Biomarkers for diagnosis and pathogenesis
Jeon et al., 2024 [76]	South Korea	Therapy	Primary	56	Mass spectrometry	n.a.	Biomarkers for BC subtypes
Johansson et al., 2015 [77]	Sweden	Therapy	Primary	24	nanoLC–MS/MS	Western blot, ELISA	Biomarkers for response to therapy
Kang et al., 2010 [79]	South Korea	Diagnosis	P and DCIS	164	MALDI–TOF MS	IHC, Western blot	Biomarkers for diagnosis and pathogenesis
Kim et al., 2009 [82]	South Korea	Diagnosis	Primary	17	MALDI–TOF MS	n.a.	Biomarkers for disease progression
Ku et al., 2025 [155]	Taiwan	Therapy	Primary	61	nanoLC–MS/MS	NGS	Biomarkers for therapy
Lin et al., 2021 [87]	China	D and T	Primary	24	iTRAQ LC–MS/MS	Western blot	Biomarkers for BC subtypes
Magara et al., 2024 [90]	Japan	Therapy	P and DCIS	133	Multilayered proteomics	Multilayered proteomics in BC cells	Biomarkers for therapy
Meric-Bernstam et al., 2014 [93]	USA	Diagnosis	primary	53	RPPA	IHC	Response to surgical treatment
Michaut et al., 2016 [94]	Netherlands	Diagnosis	Primary	55	RPPA	cDNA-microarrays, DNA-sequencing, Western blot	Biomarkers for BC subtypes
Moriggi et al., 2018 [96]	Italy	Diagnosis	Primary	26	2-DE, MALDI–MS	Gene expression microarrays, immunoblotting.	Biomarkers for BC subtypes
Nakagawa et al., 2006 [97]	USA	Diagnosis	Primary	65	SELDI–TOF MS	n.a.	Biomarkers for nodal status
Neubauer et al., 2008 [98]	Germany	Diagnosis	Primary	16	Quantitative multiplex proteomics	Western blot, immunofluoresence	Biomarkers for BC subtypes
Neubauer et al., 2006 [99]	Germany	Diagnosis	Primary	24	MALDI–TOF MS	n.a.	Biomarkers for response to therapy
Niméus et al., 2007 [100]	Sweden	D and T	Primary	20	MALDI–TOF-TOF MS	transcriptomics	Biomarkers for response to therapy
Ou et al., 2008 [101]	Singapore	Diagnosis	Primary	63	MALDI–TOF MS	cDNA microarrays, IHC, Western blot	Biomarkers for diagnosis and pathogenesis
Panis et al., 2013 [102]	Brazil	Diagnosis	P and M	135	label-free MS	Western blot, ELISA, IHC	Biomarkers for staging
Pozniak et al., 2016 [107]	Israel	Diagnosis	Primary	41/25	MS	Pulsed-SILAC Assay, IHC	High similarity in protein expression
Procházková et al., 2017 [108]	Czech Republic	Diagnosis	Primary	96	mTRAQ labeling (mTRAQ–SRM)	Transcriptomics, IHC	Biomarkers for disease progression
Pucci-Minafra et al., 2017 [109]	Italy	Diagnosis	Primary	13	2D gel electrophoresis and MS	Western blot	Biomarkers for prognosis
Pucci-Minafra et al., 2007 [110]	Italy	Diagnosis	Primary	37	MALDI–TOF	Western blot	Biomarkers for diagnosis and pathogenesis
Roberts et al., 2004 [112]	Australia	Diagnosis	Primary	27	2-DE	Western blot	Biomarkers for diagnosis and pathogenesis
Rojas et al., 2019 [113]	Spain	Diagnosis	P and M	51	PRM targeted proteomics	n.a.	Biomarkers for prediction of distant recurrence
Ruckhäberle et al., 2010 [114]	Germany	Diagnosis	Primary	19	Isobaric TMT label-based proteomics	Western blot, IHC, RNA expression microarray, LC–MS	Biomarkers for BC subtypes
Sanders et al., 2008 [116]	USA	Diagnosis	primary	122	MALDI–TOF MS	LC–MS/MS, IHC, HPLC	Biomarkers for disease progression, diagnosis and subtypes
Shenoy et al., 2020 [120]	Israel	Therapy	Primary	113	LC–MS/MS-based proteomic analysis	IHC, in vitro assays	Biomarkers for response to therapy
Shi et al., 2024 [121]	China	Therapy	Primary	50	MS-Based Label-Free Proteomics	in vitro assays	Biomarkers for therapy
Shin et al., 2020 [122]	South Korea	Diagnosis	P and M	36	Reversed-phase (RP)-nano LC–ESI–MS/MS	in vitro assays	Biomarkers for prediction of distant recurrence
Sohn et al., 2013 [124]	USA	Therapy	Primary	54	RPPA	n.a.	Biomarkers for response to therapy
Stemke-Hale et al., 2008 [126]	USA	D and T	Primary	547	RPPA	LC–MS/MS, IHC, Western blot, transcriptomics	Biomarkers for therapy
Tamesa et al., 2009 [128]	Japan	Diagnosis	Primary	40/30	LC–MS/MS	Western blot, transcriptomics	Biomarkers for diagnosis
Tyanova et al., 2016 [131]	Germany	Diagnosis	Primary	40	MS analysis	n.a.	Biomarkers for BC subtypes
Valo et al., 2019 [132]	France	Diagnosis	P and DCIS	72	SWATH–MS	IHC, ELISA	Biomarkers for diagnosis
Yang et al., 2016 [138]	China	Therapy	Primary	36	LC–MS/MS	n.a.	Biomarkers for response to therapy
Yang et al., 2015 [139]	China	Diagnosis	Primary	60	LC–MS/MS	n.a.	Biomarkers for BC subtypes
Yang et al., 2014 [140]	China	Diagnosis	Primary	36	LC–MS/MS	in vitro assays	Biomarkers for response to therapy
Yang et al., 2012 [141]	South Korea	Therapy	P and M	83	LC–MS/MS	IHC Western blot	Biomarkers for response to therapy
Yanovich et al., 2018 [142]	Israel	Diagnosis	Primary	109	LC–MS	n.a.	Biomarkers for BC subtypes
Zeng et al., 2017 [145]	China	Diagnosis	Primary	23/23	Quantitative iTRAQ	IHC	Biomarkers for prediction of distant recurrence
Zhang et al., 2008 [149]	Singapore	Diagnosis	primary	94	MALDI–TOF–TOF MS	IHC Western blot	Biomarkers for prognosis
Zhang et al., 2005 [150]	Singapore	Diagnosis	Primary	25	MALDI–TOF	IHC western blot, transcriptomics	Biomarkers for BC subtypes
Zhong et al., 2018 [153]	China	Diagnosis	Primary	54/54	iTRAQ	IHC	Biomarkers for nodal status

BC = breast cancer; CISH = Chromogenic in situ hybridization; D = Diagnosis; DCIS = Ductal carcinoma in situ; HPLC = High-Performance Liquid Chromatography; IA/MPD = Immunoassay using multiphoton-detection; IHC = Immunohistochemistry; iTRAQ = Isobaric Tag for Relative and Absolute Quantification; LC/ESI-MS/MS = Liquid Chromatography, Electrospray Ionization, Mass spectrometry; LC–MS/MS = Liquid chromatography–tandem mass spectrometry; M = Metastasis; MALDI–TOF = Matrix-assisted laser desorption/ionization-time of flight; MS = Mass spectrometry; n.a. = not available; nano-LC–MS/MS = nanoscale liquid chromatography–tandem mass spectrometry; P = Primary; PRM = Parallel reaction monitoring; RPLC = Reversed-phase liquid chromatography; RPPA = Reverse Phase Protein Arrays; SDS-PAGE = Sodium dodecyl sulfate polyacrylamide gel electrophoresis; SELDI–TOF= Surface-Enhanced Laser Desorption/Ionization Time-of-Flight; SRM-MS = Selected Reaction Monitoring Mass Spectrometry; SWATH = Sequential Window Acquisition of all Theoretical Mass Spectra; T = Therapy; TMT = Tandem Mass Tag; 2D-PAGE = Two-dimensional polyacrylamide gel electrophoresis.

**Table 2:** Proteomic studies in plasma and serum from breast cancer patients.

Study	Country	Setting	BC Stage	Type of Specimen	Number of BC Specimens	Primary Proteomic Method	Additional Laboratory Methods	Main Findings
Alvarez et al., 2022 [19]	USA	Therapy	Primary	Plasma Evs	17	PPLC and LC–MS/MS	Western blot	Biomarkers for response to therapy
An et al., 2022 [21]	China	Diagnosis	Primary	Plasma	107	Nano-LC–MS/MS	Metabolomic analysis	Biomarkers for diagnosis
Belluco et al., 2007 [26]	Italy	Diagnosis	Primary	Serum	155	SELDI–TOF MS	n.a.	Biomarkers for early diagnosis
Bera et al., 2020 [27]	USA	Diagnosis	Primary	Serum	240	Antibody Microarrays and MSD Multi-array	n.a.	Biomarkers for recurrence prediction
Corrêa et al., 2017 [41]	Brazil	Diagnosis	Primary	Plasma	107	Nano-LC–MS/MS	IHC, FISH, Western blot	Biomarkers for BC subtypes
Dalenc et al., 2010 [43]	France	Therapy	Metastatic	Serum	57	SELDI–TOF MS	LC–MS/MS	Biomarkers for response to therapy
Drukier et al., 2006 [46]	USA	Diagnosis	Primary	Serum	264	IA/MPD	ELISA, Luminex	Biomarkers for early diagnosis
Fernandez-Pol et al., 2005 [48]	USA	D and T	P and M	Serum	243	HPLC	MS, Western blot, Radioimmunoassay	Biomarkers for diagnosis and response to therapy
Fredolini et al., 2020 [50]	USA	Diagnosis	Primary	Serum	20	Affinity hydrogel nanoparticles coupled with LC–MS/MS	n.a.	Biomarkers for early diagnosis
Gajbhiye et al., 2017 [51]	India	Diagnosis	Primary	Serum	76	2D-DIGE, iTRAQ and SWATH–MS	n.a.	Biomarkers for BC subtypes
Garisi, Tommasi et al., 2012 [57]	Italy	Diagnosis	Primary	Serum	192	SELDI–TOF MS	n.a.	Biomarkers for BC subtypes
Garisi, Tufaro et al., 2012 [58]	Italy	Diagnosis	Primary	Serum	138	SELDI–TOF MS	n.a.	Biomarkers for diagnosis
Gast et al., 2011 [59]	Netherlands	Diagnosis	primary	Serum	82	SELDI–TOF MS	MALDI–TOF	Biomarkers for recurrence prediction
Goncalves et al., 2006 [61]	France	Diagnosis	primary	Serum	81	SELDI–TOF MS	Immunodepletion	Biomarkers for recurrence prediction
Grassmann et al., 2024 [64]	Sweden	Diagnosis	primary	Plasma	796	Proximity Extension Assay	n.a.	No benefit for recurrence prediction
Henderson et al., 2019 [70]	USA	Diagnosis	P and DCIS	Serum	123	Modified ECL based ELISA	n.a.	Biomarkers for early diagnosis
Henderson et al., 2016 [71]	USA	Diagnosis	P and DCIS	Serum	100	modified ELISA	n.a.	Biomarkers for early diagnosis
Hu et al., 2005 [72]	China	Diagnosis	P and M	Serum	49	SELDI–TOF MS	n.a.	Biomarkers for early diagnosis
Jordan et al., 2020 [77]	USA	D and T	primary	Plasma Evs	20	MS	Western blot, in vitro assays, Multiplex gene expression analysis	Biomarkers for diagnosis and therapy
Kaur et al., 2024 [79]	USA	Diagnosis	P and M	Serum	73	MS	n.a.	Biomarkers for disease progression
Kim et al., 2019 [81]	South Korea	Diagnosis	primary	Plasma	575	MRM MS	n.a.	Biomarkers for early diagnosis
Le Naour et al., 2001 [83]	USA	Diagnosis	primary	Serum	30	MALDI–TOF	Western blot, IHC	Biomarkers for early diagnosis
Li et al., 2024 [85]	China	Therapy	primary	Plasma	40	MS	Protein-protein interaction (PPI) analysis and Single-cell RNA sequencing	Biomarkers for response to therapy
Lötsch et al., 2022 [87]	Germany	Diagnosis	primary	Serum	27	PEA	n.a.	Biomarkers for neuropathic pain
Lourenco et al., 2017 [88]	USA	Diagnosis	primary	Serum	26	modified ELISA	n.a.	Biomarkers for early diagnosis
Majidzadeh-A et al., 2013 [90]	Iran	Therapy	primary	Serum	10	MALDI–TOF	n.a.	Biomarkers for response to therapy
Minton et al., 2013 [94]	UK	Diagnosis	primary	Serum	45	SELDI–TOF MS	LC–MS	Biomarkers for cancer Related Fatigue Syndrome
Pires et al., 2019 [106]	Brazil	Therapy	primary	Plasma	200	MS	Oxidative Stress Analyses	Biomarkers for response to therapy
Riley et al., 2011 [111]	USA	Diagnosis	P and DCIS	Serum	216	LC–MS/MS	n.a.	Biomarkers for diagnosis
Rui et al., 2003 [113]	China	Diagnosis	primary	Serum	145	MALDI–TOF	N-terminal sequencing	Biomarkers for diagnosis
Santana et al., 2024 [117]	Brazil	Diagnosis	primary	Plasma	143	LC–MS/MS	n.a.	Biomarkers for BC subtypes
Schaub et al., 2009 [119]	USA	Diagnosis	P, M and DCIS	Serum	125	MALDI–TOF MS	n.a.	Biomarkers for staging and nodal status
Sinha et al., 2023 [123]	USA	Diagnosis	primary	Saliva and Serum	15	iTRAQ analysis	n.a.	Biomarkers for early diagnosis
Starodubtseva et al., 2023 [125]	Russia	Diagnosis	metastatic	Serum	25	LC–MRM MS	Lipidomics	Biomarkers differentiating primary from metastatic BC
Suman et al., 2016 [127]	India	Diagnosis	primary	Plasma	32	iTRAQ analysis	Western blot, ELISA and IHC	Biomarkers for BC subtypes
Tomar et al. [154]	India	Diagnosis	P and M	Serum	12	MS	n.a	Biomarkers for diagnosis
Tutanova et al., 2020 [130]	Russia	Diagnosis	primary	Plasma, WBE	23	MS	Flow Cytometry	Biomarkers for diagnosis
Vinik et al., 2020 [133]	USA	Diagnosis	primary	Plasma Evs	52	RPPA	Immunoblotting	Biomarkers for early diagnosis
Xu et al., 2015 [135]	China	Diagnosis	primary	Serum	60	LC–MS/MS	ELISA	Biomarkers for response to therapy
Xu et al., 2024 [134]	China	Diagnosis	primary	Serum Evs	126	MS	IHC and in vitro cell assays	Biomarkers for early diagnosis and staging
Yan et al., 2022 [136]	China	Diagnosis	primary	Serum	64	MB–IMAC-Cu and MALDI–TOF MS	LC-ESI-MS/MS, ELISA	Biomarkers for diagnosis
Yang et al., 2020 [137]	China	Therapy	primary	Serum	51	Isobaric TMT label-based quantitative proteomics	LC–MS/MS	Biomarkers for response to therapy
Ye et al., 2024 [143]	China	Diagnosis	P and M	Plasma	51	UPLC–MS/MS	ELISA, metabolomics	Biomarkers differentiating different sites of metastasis
Zeidan et al., 2018 [144]	UK	Diagnosis	primary	Serum	399	LC–MS/MS	ELISA	Biomarkers for staging and nodal status
Zhang et al., 2013 [148]	USA	Diagnosis	P and DCIS	Serum	100	LC–MS/MS	n.a.	Biomarkers for early diagnosis
Zhang et al., 2015 [149]	USA	Diagnosis	primary	Serum	80	LC–MS/MS	n.a.	Biomarkers for early diagnosis
Zhang et al., 2013 [150]	USA	Diagnosis	primary	Plasma	80	LC–ESI–MS/MS	n.a.	Biomarkers for early diagnosis
Zhao et al., 2024 [152]	China	Diagnosis	primary	Plasma	10	LC–MS/MS	n.a.	Biomarkers for BC subtypes

BC = breast cancer; ECL = electro-chemiluminescent; EVs = extracellular vesicles; iTRAQ = Isobaric Tag for Relative and Absolute Quantification; LC = Liquid chromatography; LC–ESI–MS/MS = Liquid Chromatography-Electrospray Ionization-Tandem Mass Spectrometry; LC–MS/MS = Liquid chromatography–tandem mass spectrometry; MALDI–TOF = Matrix-assisted laser desorption/ionization-time of flight; MB–IMAC = Magnetic beads based immobilized metal ion affinity chromatography; MRM = Multiple reaction monitoring; MS = Mass spectrometry; PEA = proximity extension assay; PPLC = Particle purification liquid chromatography; RPPA = Reverse Phase Protein Arrays; SELDI–TOF= Surface-Enhanced Laser Desorption/Ionization Time-of-Flight; SWATH = Sequential Window Acquisition of all Theoretical Mass Spectra; TMT = Tandem Mass Tag; 2D-DIGE = two-dimensional difference gel electrophoresis; UPLC = Ultra-performance liquid chromatography; WBE = whole blood exosomes.

**Table 3:** Proteomic studies in nipple aspiration fluid (NAF), urine, saliva, tear fluid, pleural effusions, tumor interstitial fluid and lymph nodes from breast cancer patients.

Study	Country	Setting	BC Stage	Type of Specimen	Number of BC Specimens	Primary Proteomic Method	Additional Laboratory Methods	Main Findings
Brunoro et al., 2019 [35]	Brazil	Diagnosis	primary	NAF	10	MS	n.a.	Biomarkers differentiating BC from healthy
Kuerer et al., 2004 [82]	USA	Diagnosis	primary	NAF	23	SELDI–TOF MS	n.a.	Biomarkers for nodal status
Paweletz et al., 2001 [104]	USA	Diagnosis	primary	NAF	12	SELDI–TOF MS	n.a.	Biomarkers differentiating BC from healthy
Pawlik et al., 2006 [105]	USA	Diagnosis	primary	NAF	18	LC–MS/MS	Western blot	Biomarkers differentiating BC from healthy
Sauter et al., 2002 [118]	USA	Diagnosis	primary	NAF	20	SELDI–TOF MS	n.a.	Biomarkers differentiating BC from healthy
Beretov et al., 2015 [28]	Australia	Diagnosis	P and DCIS	Urine	20	LC–MS/MS	Western blot, IHC	Biomarkers differentiating BC from healthy
Gajbhiye et al., 2016 [52]	India	Diagnosis	primary	Urine	43	2D-DIGE, iTRAQ, SWATH MS	Western blot	Biomarkers differentiating BC from healthy
Jeanmard et al., 2023 [74]	Thailand	Diagnosis	primary	Urinary EVs	47	LC–MS/MS	Western blot	Biomarkers differentiating BC from healthy
Giri et al., 2022 [60]	India	Diagnosis	metastatic	Saliva	20	PRM–MS	Western blot	Biomarkers differentiating BC from healthy
Zhang et al., 2010 [151]	USA	Diagnosis	primary	saliva	40	MALDI–TOF	Western blot	Biomarkers differentiating BC from healthy
Sinha et al., 2023 [123]	USA	Diagnosis	primary	Saliva, Serum	15	iTRAQ proteomic analysis	n.a.	Biomarkers differentiating BC from healthy
Böhm et al., 2012 [31]	Germany	Diagnosis	primary	Tear fluid	25	MALDI–TOF	n.a.	Biomarkers differentiating BC from healthy
Lebrecht et al., 2009 [84]	Germany	Diagnosis	primary	Tear fluid	50	SELDI–TOF MS	n.a.	Biomarkers differentiating BC from healthy
Mayayo-Peralta et al., 2024 [91]	Netherlands	Therapy	metastatic	Pleural effusions	47	Phosphoproteomics analysis	n.a.	Biomarkers for response to therapy
Terkelsen et al., 2020 [129]	Denmark	Diagnosis	primary	TIF	35	LC–MS/MS	IHC	Biomarkers for BC subtypes
Pathania et al., 2022 [103]	India	Diagnosis	primary	SLN	13	iTRAQ proteomic analysis and MS	ELISA	Biomarkers for nodal status
Pozniak et al., 2016 [107]	Israel	Diagnosis	primary	BC, LN	41+25	MS	Pulsed-SILAC, IHC	No difference in protein expression
Zeng et al., 2017 [145]	China	Diagnosis	primary	BC, LN	23+23	iTRAQ proteomic analysis	IHC	Biomarkers for nodal status
Zhong et al., 2018 [153]	China	Diagnosis	primary	BC, LN	54+54	iTRAQ proteomic analysis	IHC	Biomarkers for nodal status

BC = breast cancer; EVs = extracellular vesicles; TIF = Tumor interstitial fluid; IHC = Immunohistochemistry; iTRAQ = Isobaric Tag for Relative and Absolute Quantification; LC–MS/MS = Liquid chromatography–tandem mass spectrometry; LN = Lymph nodes; MALDI–TOF = Matrix-assisted laser desorption/ionization-time of flight; MS = Mass spectrometry; NAF = Nipple aspiration fluid; PRM = Parallel reaction monitoring; SELDI–TOF = Surface-Enhanced Laser Desorption/Ionization Time-of-Flight; SLN = Sentinel lymph nodes; SWATH = Sequential Window Acquisition of all Theoretical Mass Spectra; 2D-DIGE = two-dimensional difference gel electrophoresis.

**Table 4:** Proteomic studies that identified biomarkers for prognosis of breast cancer patients.

Study	BC Stage	Type of Specimen	Number of BC Specimens	Primary Proteomic Method	Additional Laboratory Methods	Main Findings
Cawthorn et al., 2012 [39]	primary	Tumor	990	iTRAQ and LC–MS/MS	IHC, SRM–MS	High expression levels of Decorin and Endoplasmin (HSP90B1) are associated with increased metastasis-poorer survival and may guide the use of hormonal therapy.
Gonzalez-Angulo et al., 2011 [63]	P, M, DCIS	Tumor	880	RPPA	IHC	A 10-protein biomarker panel was developed that classifies breast cancer into prognostic groups that may have potential utility in the management of patients who receive anthracycline-taxane-based neoadjuvant systemic therapy.
Bernhardt et al., 2017 [29]	primary	Tumor	801	RPPA	IHC	SHMT2 and ASCT2 protein expression were identified as novel potential prognostic biomarkers for BC, as their high protein expression is associated with poor outcome.
Asleh et al., 2022 [22]	primary	Tumor	300	LC–MS/MS-based proteomics	IHC	Potential diagnostic and prognostic biomarkers were identified.
Cancemi et al., 2012 [37]	primary	Tumor	100	MALDI–TOF MS	IHC, Western blot	Deregulation of proteins of S100 family is associated with breast cancer progression and may serve as potential prognostic biomarkers for patient stratification.
Cancemi et al., 2010 [38]	primary	Tumor	100	MALDI–TOF MS	Western blot, n terminal microsequencing	S100A7 protein, with its two isoforms, serve a potential role in the progression and biological mechanisms of infiltrating ductal carcinoma.
Procházková et al., 2017 [108]	primary	Tumor	96	mTRAQ labeling (mTRAQ-SRM)	Transcriptomics, IHC	A panel of gene products that can contribute to breast cancer aggressiveness and metastasis was identified.
Zhang et al., 2008 [149]	primary	Tumor	94	MALDI–TOF-TOF MS	IHC Western blot	CK19 in HER-2+ breast cancer is associated with tumor aggressiveness, suggesting CK19’s potential role as a biomarker for identifying more aggressive BC subtypes.
Di Cara et al., 2019 [45]	primary	Tumor	80	MALDI–TOF MS	n.a.	MMP-2 and MMP-9 can be involved in the complicated scenario in which the mechanisms of tumor progression are correlated with unfavorable prognosis.
Kaur et al., 2024 [79]	P and M	Serum	73	MS	n.a.	A set of proteins that could be involved in breast cancer progression in serum was identified.
Gámez-Pozo et al., 2017 [54]	primary	Tumor	60	LC–MS	Parallel reaction monitoring	Some ER+/PR+ samples had a protein expression profile similar to that of triple negative breast cancer (TNBC) and had a clinical outcome similar to those with TNBC.

BC = breast cancer; DCIS = Ductal carcinoma in situ; IHC = Immunohistochemistry; iTRAQ = Isobaric Tag for Relative and Absolute Quantification; LC–MS/MS = Liquid chromatography–tandem mass spectrometry; M = Metastasis; MALDI–TOF = Matrix-assisted laser desorption/ionization-time of flight; MS = Mass spectrometry; n.a. = not available; P = Primary; RPPA = Reverse Phase Protein Arrays; SRM–MS = Selected Reaction Monitoring Mass Spectrometry.

**Table 5:** Proteomic studies that identified biomarkers for early diagnosis of breast cancer.

Study	BC Stage	Type of Specimen	Number of BC Specimens	Primary Proteomic Method	Additional Methods	Main Findings
Grassmann et al., 2024 [64]	primary	Plasma	796	Proximity Extension Assay	n.a.	No benefit for recurrence prediction
Kim et al., 2019 [81]	primary	Plasma	575	MRM MS	n.a.	Three specific peptides can be a useful tool for breast cancer screening and its accuracy is cancer-type specific.
Drukier et al., 2006 [46]	primary	Tumor	264	IA/MPD	ELISA, Luminex	Ultrasensitive, multi-biomarker immunoassays significantly improve early breast cancer detection accuracy.
Belluco et al., 2007 [26]	primary	Serum	155	SELDI–TOF MS	n.a.	A proteomic pattern consisting of 7 low-molecular-weight ion peaks is a highly sensitive and specific method for early detection of stage 1 breast cancer.
Xu et al., 2024 [134]	primary	Serum Evs	126	MS	IHC and in vitro cell assays	Proteins carried by breast cancer–derived EVs could be used as minimally invasive liquid biopsy tool for the early detection of breast cancer and for discriminating lymph node involvement and distant metastasis.
Henderson et al., 2019 [70]	P and DCIS	Serum	123	Modified ECL based ELISA	n.a.	Serum biomarkers provide clinicians with additional information for patients with indeterminate breast imaging results, potentially reducing false-positive breast biopsies.
Zhang et al., 2013 [148]	P and DCIS	Serum	100	LC–MS/MS	n.a.	Feed Forward Neural Network (FFNN) enhances the development of more accurate and reliable biomarker panels for the early diagnosis of breast cancer.
Henderson et al., 2016 [71]	P and DCIS	Serum	100	modified ELISA	n.a.	SPB and TAAb combinatorial protein biomarker assays may aid in the detection of early BC and guide decisions between imaging and tissue biopsy.
Zhang et al., 2015 [149]	primary	Serum	80	LC–MS/MS	n.a.	Pathway-based biomarkers can significantly enhance the early detection and diagnostic accuracy of breast cancer.
Zhang et al., 2013 [150]	primary	Plasma	80	LC-ESI-MS/MS	n.a.	Identification of eight alternative splicing isoform biomarkers can assist the early diagnosis of breast cancer.
Vinik et al., 2020 [133]	primary	Plasma Evs	52	RPPA	Immunoblotting	Several potential markers that could contribute to early detection of BC were identified.
Hu et al., 2005 [72]	P and M	Serum	49	SELDI–TOF MS	n.a.	SELDI–TOF-MS combined with bioinformatics tools is a promising approach for the early detection of breast cancer, identifying four candidate biomarkers.
Le Naour et al., 2001 [83]	primary	Serum	30	MALDI–TOF	Western blot, IHC	RS/DJ-1 is a novel circulating tumor antigen eliciting a humoral immune response in breast cancer patients and can be used for early detection and monitoring of BCr.

BC = breast cancer; DCIS = Ductal carcinoma in situ; EVs = extracellular vesicles; IA/MPD = Immunoassay using multiphoton-detection; IHC = Immunohistochemistry; iTRAQ = Isobaric Tag for Relative and Absolute Quantification; LC/ESI-MS/MS = Liquid Chromatography, Electrospray Ionization, Mass spectrometry; LC–MS/MS = Liquid chromatography–tandem mass spectrometry; M = Metastasis; MALDI–TOF = Matrix-assisted laser desorption/ionization-time of flight; MS = Mass spectrometry; n.a. = not available; P = Primary; RPPA = Reverse Phase Protein Arrays; SELDI–TOF = Surface-Enhanced Laser Desorption/Ionization Time-of-Flight.

**Table 6:** Proteomic studies that identified biomarkers for characterization of breast cancer subtypes.

Study	BC Stage	Type of Specimen	Number of BC Specimens	Primary Proteomic Method	Additional Laboratory Methods	Main Findings
Creighton et al., 2010 [42]	primary	Tumor	429	RPPA	In vitro assays and quantitative real-time PCR	Hyperactive PI3K signaling is associated with low estrogen receptor (ER) levels and luminal B molecular subtype in ER-positive BC.
Garisi et al., 2012 [57]	primary	Serum	192	SELDI–TOF MS	n.a.	The serum profile of familial breast cancer patients was different when compared with that of sporadic breast cancer patients.
Santana et al., 2024 [117]	primary	Plasma	143	LC–MS/MS	n.a.	The HDL proteome showed discriminatory abilities across different clinical stages of breast cancer and a distinct profile in triple negative breast cancer.
Sanders et al., 2008 [116]	primary	Tumor	122	MALDI–TOF MS	LC–MS/MS, IHC, HPLC	S100A6 (calcyclin) and S100A8 (calgranulin A) highlight potential roles in cancer progression, diagnosis, and molecular classification.
Yanovich et al., 2018 [142]	primary	Tumor	109	LC–MS	n.a.	A novel luminal subtype characterized by increased PI3K signaling has been identified.
Corrêa et al., 2017 [41]	primary	Plasma	107	Nano-LC–MS/MS	IHC, FISH, Western blot	The plasma proteomic profile of breast cancer subtypes was determined.
Gámez-Pozo et al., 2017 [53]	primary	Tumor	106	LC–MS	MicroRNA expression analysis	ER+ BC and triple negative breast cancer exhibit distinct molecular and metabolic profiles.
Fonseca-Sánchez et al., 2012 [49]	primary	Tumor	105	LC/ESI-MS/MS	IHC, Western blot	Glyoxalase 1 (GLO1) is overexpressed in breast cancer and correlates significantly with high tumor grade.
Gámez-Pozo et al., 2015 [55]	primary	Tumor	96	LC–MS/MS	MicroRNA expression and in vitro assays	Some ER+/PR+ samples had a protein expression profile similar to that of triple negative breast cancer (TNBC) and had a clinical outcome similar to those with TNBC.
Gajbhiye et al., 2017 [51]	primary	Serum	76	2D-DIGE, iTRAQ and SWATH-MS	n.a.	Serum proteome alterations may help to distinguish breast cancer subtypes (luminal A and, B, HER2-positive and triple negative BCr).
Jeon et al., 2024 [76]	primary	Tumor	56	Mass spectrometry	n.a.	Coronin-1A and titin were upregulated in the immune-inflamed subtype, and α-1-antitrypsin was upregulated in the immune-excluded/desert subtype.
Michaut et al., 2016 [94]	primary	Tumor	55	RPPA	cDNA-microarrays, DNA-sequencing, Western blot	Two biologically distinct subtypes of invasive lobular breast cancer were identified.
He et al., 2009 [69]	primary	Tumor	52	SELDI–TOF MS	IHC	Protein biosignatures were identified as having potential utility in tumor classification and predicting therapeutic responses.
Tyanova et al., 2016 [131]	primary	Tumor	40	MS analysis	n.a.	Global profiling of breast cancer clinical samples allows the attribution of biological processes to the different breast cancer subtypes (ER/PR, HER2 positive and TNBC).
Yang et al., 2014 [140]	primary	Tumor	36	LC–MS/MS	in vitro assays	The level of FR isoforms was associated with several histopathological features and molecular subtypes.
Terkelsen et al., 2020 [129]	primary	TIF	35	LC–MS/MS	IHC	Ten proteins, AGR3, BCAM, CELSR1, MIEN1, NAT1, PIP4K2B, SEC23B, THTPA, TMEM51, and ULBP2 stratify the tumor subtype-specific TIFs.
Suman et al., 2016 [127]	primary	Plasma	32	iTRAQ analysis	Western blot, ELISA and IHC	Four proteins (FN1, A2M, C4BPA and CFB) had strong association with molecular subtypes of breast cancer.

BC = breast cancer; DCIS = Ductal carcinoma in situ; HPLC = High-Performance Liquid Chromatography; IHC = Immunohistochemistry; iTRAQ = Isobaric Tag for Relative and Absolute Quantification; LC/ESI-MS/MS = Liquid Chromatography, Electrospray Ionization, Mass spectrometry; LC–MS/MS = Liquid chromatography–tandem mass spectrometry; M = Metastasis; MALDI–TOF = Matrix-assisted laser desorption/ionization-time of flight; MS = Mass spectrometry; n.a. = not available; nano-LC–MS/MS = nanoscale liquid chromatography–tandem mass spectrometry; P = Primary; RPPA = Reverse Phase Protein Arrays; SELDI–TOF = Surface-Enhanced Laser Desorption/Ionization Time-of-Flight; SWATH = Sequential Window Acquisition of all Theoretical Mass Spectra; TIF = Tissue Interstitial Fluid TMT = Tandem Mass Tag.

**Table 7:** Proteomic studies that identified biomarkers for response to treatment in breast cancer patients.

Study	BC Stage	Type of Specimen	Number of BC Specimens	Primary Proteomic Method	Additional Laboratory Methods	Main Findings
Gonzalez-Angulo et al., 2011 [63]	P, M, DCIS	Tumor	880	RPPA	IHC	A 10-protein biomarker panel was developed that classifies breast cancer into prognostic groups that may have potential utility in the management of patients who receive anthracycline-taxane-based neoadjuvant systemic therapy.
Fernandez-Pol et al., 2005 [48]	P and M	Serum	243	HPLC	MS, Western blot, Radioimmunoassay	MPS-1 is useful for early detection, monitoring, and management of breast cancer, superior than CA-15-3 and CEA.
Pires et al., 2019 [106]	primary	Plasma	200	MS	Oxidative Stress Analyses	The connection between inflammation, the complement and oxidative stress seems to be a pivotal axis in chemoresistance of luminal A breast cancer.
Bonneterre et al., 2013 [32]	primary	Tumor	149	SELDI–TOF MS	n.a.	A combined signature using the cytosol and plasma proteomic data may identify breast cancer patients like to achieve complete response in neoadjuvant chemotherapy.
Duan et al., 2023 [47]	primary	Tumor	139	MS	n.a.	115 proteins were differentially expressed between patients with pathologic Complete Response (pCR) and the non-pCR group.
Shenoy et al., 2020 [120]	primary	Tumor	113	LC–MS/MS-based proteomic analysis	IHC, in vitro assays	Two proteins of proline biosynthesis pathway, PYCR1 and ALDH18A1, were significantly associated with resistance to treatment.
Yang et al., 2012 [141]	P and M	Tumor	83	LC–MS/MS	IHC Western blot	ERK/Bcl-2-mediated anti-apoptosis was investigated in general and in the development of drug resistance.
Xu et al., 2015 [135]	primary	Serum	60	LC–MS/MS	ELISA	SISCAPA-targeted proteomics alllowed quatification of low-abundant serum transferrin receptor in breast cancer patients pre- and post-chemotherapy.
Yang et al., 2015 [139]	primary	Tumor	60	LC–MS/MS	n.a.	TfR levels in breast tissue can be measured precisely with LC–MS/MS and could possibly improve the diagnosis of breast cancer and assessment of drug resistance.
Dalenc et al., 2010 [43]	metastatic	Serum	57	SELDI–TOF MS	LC–MS/MS	Fibrinogen α peptide could serve as a predictive biomarker for therapeutic response in ER+ breast cancer patients undergoing the tipifarnib and tamoxifen combination therapy.
Sohn et al., 2013 [124]	primary	Tumor	54	RPPA	n.a.	AKT, IGFBP2, LKB1, S6 and Stathmin predict relapse-free survival (RFS) in residual triple negative BC patients after neoadjuvant chemotherapy, while PI3K pathway may represent a potential therapeutic target.
Meric-Bernstam et al., 2014 [93]	primary	Tumor	53	RPPA	IHC	PI3K pathway activation is greater in core needle biopsy compared with postexcision surgical samples, suggesting a potential loss of phosphorylation during surgical manipulation, or with cold ischemia of surgical specimens.
He et al., 2009 [69]	primary	Tumor	52	SELDI–TOF MS	IHC	Protein biosignatures were identified as having potential utility in tumor classification and predicting therapeutic responses.
Yang et al., 2020 [137]	primary	Serum	51	Isobaric TMT quantitative proteomics	LC–MS/MS	A serum-based protein signature that potentially predicts the therapeutic effects of trastuzumab-based therapy for HER2-positive breast cancer patients was developed.
Mayayo-Peralta et al., 2024 [91]	metastatic	Pleural effusions	47	Phosphoproteomics analysis	n.a.	Evidence for decreased activity of several key kinases in ERα-converted metastases was found.
Guerin et al., 2018 [66]	primary	Tumor	46	MS RPLC	Western blot	Differential gene expression correlated with sensitivity to trastuzumab.
Debets et al., 2023 [44]	primary	Tumor	45	Phosphoproteomics	n.a.	Treatment response to trastuzumab, pertuzumab may be predicted.
Gromova et al., 2021 [65]	primary	Tumor	44	RPPA	IHC and PCR	High-level c-Kit expression is a frequent event in triple negative BC (TNBC), and activating mutations were also present, suggesting a potential effect of c-Kit inhibitors on TNBCs.
Li et al., 2024 [85]	primary	Plasma	40	MS	Protein-protein interaction (PPI) analysis and Single-cell RNA sequencing	Specific plasma proteins act as predictive biomarkers of response to immunotherapy.
He et al., 2011 [68]	primary	Tumor	39	LC–MS	IHC	Proteomic profiling of breast cancer tissues can predict tumor response to neoadjuvant chemotherapy.
Yang et al., 2016 [138]	primary	Tumor	36	LC–MS/MS	n.a.	FKBP4 and S100A9 are promising predictive biomarkers for determining drug resistance in breast cancer patients undergoing neoadjuvant chemotherapy.

BC = breast cancer; DCIS = Ductal carcinoma in situ; HPLC = High-Performance Liquid Chromatography; IHC = Immunohistochemistry; LC–MS/MS = Liquid chromatography–tandem mass spectrometry; M = Metastasis; MALDI–TOF = Matrix-assisted laser desorption/ionization-time of flight; MS = Mass spectrometry; n.a. = not available; nano-LC–MS/MS = nanoscale liquid chromatography–tandem mass spectrometry; P = Primary; PRM = Parallel reaction monitoring; RPLC = Reversed-phase liquid chromatography; RPPA = Reverse Phase Protein Arrays; SELDI–TOF = Surface-Enhanced Laser Desorption/Ionization Time-of-Flight; TMT = Tandem Mass Tag.

## Data Availability

The original data presented in the study are openly available inMedline/PubMed (https://pubmed.ncbi.nlm.nih.gov/, accessed on 23 March 2025), and EMBASE/Scopus (https://www.elsevier.com/products/scopus, accessed on 23 March 2025).

## Supplementary Materials

The following supporting information can be downloaded at: https://www.mdpi.com/article/10.3390/jpm15050177/s1, Supplementary Table S1. PRISMA-ScR Checklist, Supplementary Table S2. Main findings and conclusions of proteomic studies in tumor tissue specimens from breast cancer patients, Supplementary Table S3. Main findings and conclusions of proteomic studies in plasma and serum from breast cancer patients. Supplementary Table S4. Main findings and conclusions of proteomic studies in nipple aspiration fluid (NAF), urine, saliva, tear fluid, pleural effusions, tumor interstitial fluid and lymph nodes from breast cancer patients. Supplementary Figure S1: Number of studies according to the year of publication. Supplementary Figure S2: Number of studies according to geographical area. Supplementary Figure S3: Number of studies according to clinical relevance. Supplementary Figure S4: Number of studies according to disease stage of analyzed clinical specimens. Supplementary File S1: List of In Vitro Studies. Supplementary File S2: List of In Silico Studies. Supplementary File S3: List of Reviews.
