# Supplementary material for: Proteomics in Diagnostic Evaluation and Treatment of Breast Cancer: A Scoping Review
Source: J Pers Med. 2025 Apr 27;15(5):177. doi: 10.3390/jpm15050177 (PMC12113354; doi:10.3390/jpm15050177)
Supplement: Supplementary file 1 [file jpm-15-00177-s001.zip › jpm-3577502-supplementary.pdf]

Zafrakas et al. Proteomics in diagnostic evaluation and treatment of breast cancer: A scoping review.

Supplementary Table S1. PRISMA-ScR Checklist.

Section	Item	PRISMA-ScR Checklist Item	Yes/No
**Title**	1	Identify the report as a scoping review	Yes
**Abstract**			
Structured summary	2	Provide a structured summary that includes (as applicable) background, objectives, eligibility criteria, sources of evidence, charting methods, results, and conclusions that relate to the review questions and objectives.	Yes
**Introduction**			
Rationale	3	Describe the rationale for the review in the context of what is already known. Explain why the review questions/objectives lend themselves to a scoping review approach.	Yes
Objectives	4	Provide an explicit statement of the questions and objectives being addressed with reference to their key elements (e.g., population or participants, concepts, and context) or other relevant key elements used to conceptualize the review questions and/or objectives.	Yes
**Methods**			
Protocol and registration	5	Indicate whether a review protocol exists; state if and where it can be accessed (e.g., a Web address); and if available, provide registration information, including the registration number.	Yes
Eligibility criteria	6	Specify characteristics of the sources of evidence used as eligibility criteria (e.g., years considered, language, and publication status), and provide a rationale.	Yes
Information sources	7	Describe all information sources in the search (e.g., databases with dates of coverage and contact with authors to identify additional sources), as well as the date the most recent search was executed.	Yes
Search	8	Present the full electronic search strategy for at least 1 database, including any limits used, such that it could be repeated.	Yes
Selection of sources of Evidence	9	State the process for selecting sources of evidence (i.e., screening and eligibility) included in the scoping review.	Yes
Data charting process	10	Describe the methods of charting data from the included sources of evidence (e.g., calibrated forms or forms that have been tested by the team before their use, and whether data charting was done independently or in duplicate) and any processes for obtaining and confirming data from investigators.	Yes

Supplementary Table S1. (continued).

Data items	11	List and define all variables for which data were sought and any assumptions and simplifications made.	Yes
Critical appraisal of individual sources of evidence	12	If done, provide a rationale for conducting a critical appraisal of included sources of evidence; describe the methods used and how this information was used in any data synthesis (if appropriate).	No
Summary measures	13	Not applicable for scoping reviews.	No
Synthesis of results	14	Describe the methods of handling and summarizing the data that were charted.	Yes
Risk of bias across studies	15	Not applicable for scoping reviews.	No
Additional analyses	16	Not applicable for scoping reviews.	No
**Results**			
Selection of sources of evidence	17	Give numbers of sources of evidence screened, assessed for eligibility, and included in the review, with reasons for exclusions at each stage, ideally using a flow diagram.	Yes
Characteristics of sources of evidence	18	For each source of evidence, present characteristics for which data were charted and provide the citations.	Yes
Critical appraisal within sources of evidence	19	If done, present data on critical appraisal of included sources of evidence (see item 12).	No
Results of individual sources of evidence	20	For each included source of evidence, present the relevant data that were charted that relate to the review questions and objectives.	Yes
Synthesis of results	21	Summarize and/or present the charting results as they relate to the review questions and objectives.	Yes
Risk of bias across studies	22	Not applicable for scoping reviews.	No
Additional analyses	23	Not applicable for scoping reviews.	No
**Discussion**			
Summary of evidence	24	Summarize the main results (including an overview of concepts, themes, and types of evidence available), link to the review questions and objectives, , and consider the relevance to key groups	Yes
Limitations	25	Discuss the limitations of the scoping review process.	Yes
Conclusions	26	Provide a general interpretation of the results with respect to the review questions and objectives, as well as potential implications and/or next steps.	Yes
**Funding**	27	Describe sources of funding for the included sources of evidence, as well as sources of funding for the scoping review. Describe the role of the funders of the scoping review.	Yes

Supplementary Table S2. Main findings and conclusions of proteomic studies in tumor tissue specimens from breast cancer patients.

Study	Setting	BC stage	Number of BC specimens	Primary Proteomic method	Main findings and conclusions
Abdullah et al. 2016 [16]	Diagnosis	primary	20	MALDI-TOF	Many types of proteins were identified as fundamental steps for developing molecular markers for diagnosis of human breast cancer.
Akcakanat et al. 2021 [17]	Therapy	metastatic	37	RPPA	Both genomic and transcriptional profiling demonstrated discordances between primary and metastatic tumors and this is important for biomarker-driven personalized therapy.
Akpinar et al. 2017 [18]	Diagnosis	primary	10	2D-PAGE	2D electrophoresis may be used to differentiate metastatic from non-metastatic forms of BC.
Al-Wajeeh et al. 2020 [20]	Diagnosis	primary	80	SDS-PAGE & LC-MS/MS	Knowledge of protein expression, especially in stage 2 and stage 3 breast cancer, can provide important clues that may enable the discovery of novel biomarkers in carcinogenesis.
Asleh et al. 2022 [22]	Diagnosis	primary	300	LC-MS/MS-based proteomics	Potential diagnostic and prognostic biomarkers were identified.
Azevedo et al. 2023 [23]	Diagnosis	primary	19	High-throughput MS	Ribosomal proteins have clinical potential as biomarkers of diagnostic and prognostic significance in breast cancer.
Azevedo et al. 2022 [24]	Diagnosis	primary	19	LC-MS/MS	Potential protein biomarkers for the stratification of breast cancer patients were identified.
Bateman et al. 2010 [25]	Diagnosis	P, R, DCIS	25	LC-MS/MS	TSP-1, CDH1, CTNNA1 and CTNNB1 were identified as biomarkers of disease progression, while IRS-1 and PARK7 as biomarkers associated with breast cancer recurrence.
Bernhardt et al. 2017 [29]	Diagnosis	primary	801	RPPA	SHMT2 and ASCT2 protein expression were identified as novel potential prognostic biomarkers for BC, as their high protein expression is associated with poor outcome.
Bjørnstad et al. 2024 [30]	Diagnosis	primary	107	MS	Neural involvement plays an active role in breast cancer.
Bonneterre et al. 2013 [32]	Therapy	primary	149	SELDI-TOF MS	A combined signature using the cytosol and plasma proteomic data may identify breast cancer patients like to achieve complete response in neoadjuvant chemotherapy.
Bouchal et al. 2015 [33]	Diagnosis	primary	160	iTRAQ-based proteomics	CPB1, PDLIM2, RNF25, RELA, STMN1, TMSB10 identify a high-risk subgroup of low-grade breast cancer patients prone to early nodal metastasis and tumor aggressiveness.

Supplementary Table S2. (continued – part 2).

Study	Setting	BC stage	Number of BC specimens	Primary Proteomic method	Main findings and conclusions
Braakman et al. 2015 [34]	Diagnosis	primary	11	nano-LC-MS/MS	Proteomic analysis using AGPC organic fractions can distinguish breast cancer molecular subtypes and support personalized diagnostic and therapeutic decision-making.
Cabezón et al. 2012 [36]	Therapy	primary	78	2D-PAGE and MS	Immunotherapy targeting MAGE-A4 might be a therapeutic option for TNBC.
Cancemi et al. 2012 [37]	Diagnosis	primary	100	MALDI-TOF MS	Deregulation of proteins of S100 family is associated with breast cancer progression and may serve as potential prognostic biomarkers for patient stratification.
Cancemi et al. 2010 [38]	Diagnosis	primary	100	MALDI-TOF MS	S100A7 protein, with its two isoforms, serve a potential role in the progression and biological mechanisms of infiltrating ductal carcinoma.
Cawthorn et al. 2012 [39]	Diagnosis	primary	990	iTRAQ and LC-MS/MS	High expression levels of Decorin and Endoplasmin (HSP90B1) are associated with increased metastasis-poorer survival and may guide the use of hormonal therapy.
Champattanachai et al. 2013 [40]	Diagnosis	primary	26	LC-MS/MS	Aberrant protein O-GlcNAcylation is associated with BC. Abnormal modification of these O-GlcNAc-modified proteins might be one of the vital malignant characteristics of cancer.
Creighton et al. 2010 [42]	D & T	primary	429	RPPA	Hyperactive PI3K signaling is associated with low estrogen receptor (ER) levels and luminal B molecular subtype in ER-positive breast cancer.
Debets et al. 2023 [44]	Therapy	primary	45	Phosphoproteomics	Treatment response to trastuzumab, pertuzumab may be predicted.
Di Cara et al. 2019 [45]	Diagnosis	primary	80	MALDI-TOF MS	MMP-2 and MMP-9 can be involved in the complicated scenario in which the mechanisms of tumor progression are correlated with unfavorable prognosis.
Duan et al. 2023 [47]	Therapy	primary	139	MS	115 proteins were differentially expressed between patients with pathologic Complete Response (pCR) and the non-pCR group.
Fonseca-Sánchez et al. 2012 [49]	Diagnosis	primary	105	LC/ESI-MS/MS	Glyoxalase 1 (GLO1) is overexpressed in breast cancer and correlates significantly with high tumor grade.
Gámez-Pozo et al. 2017 [53]	Diagnosis	primary	106	LC-MS	ER+ BC and triple negative breast cancer exhibit distinct molecular and metabolic profiles.
Gámez-Pozo et al. 2017 [54]	Diagnosis	primary	60	LC-MS	Some ER+/PR+ samples had a protein expression profile similar to that of triple negative breast cancer (TNBC) and had a clinical outcome similar to those with TNBC.
Gámez-Pozo et al. 2015 [55]	D & T	primary	96	LC-MS/MS	A protein combination signature was identified, that complements histopathological prognostic factors in triple negative TNBC treated with adjuvant chemotherapy.

Supplementary Table S2 (continued – part 3)

Study	Setting	BC stage	Number of BC specimens	Primary Proteomic method	Main findings and conclusions
García-Adrián et al. 2021 [56]	Therapy	primary	125	MS	Two molecular groups were identified showing several differentially expressed proteins, that may serve to design new therapeutic strategies in the future.
Gonzalez-Angulo et al. 2013 [62]	Therapy	primary	175	RPPA	CHK1pS345, Caveolin1, and RAB25 for residual BC and CD31 and Cyclin E1 for hormone-receptor-positive breast cancer predicted relapse-free survival (RFS), offering potential therapeutic targets and tools for risk stratification in resistant BCr.
Gonzalez-Angulo et al. 2011 [63]	D & T	P, M, DCIS	880	RPPA	A 10-protein biomarker panel classifies breast cancer into prognostic subgroups, predict relapse-free survival (RFS) and pathologic Complete Response (pCR) to neoadjuvant therapy.
Gromova et al. 2021 [65]	Therapy	primary	44	RPPA	High-level c-Kit expression is a frequent event in triple negative BC (TNBC), and activating mutations were also present, suggesting a potential effect of c-Kit inhibitors on TNBCs.
Guerin et al. 2018 [66]	Therapy	primary	46	MS RPLC	Differential gene expression correlated with sensitivity to trastuzumab.
Gustafsson et al. 2024 [67]	Diagnosis	primary	63	LC-MS/MS	Different qualities of tumor microenvironment have the potential to add clinically relevant information for breast cancer.
He et al. 2011 [68]	D & T	primary	39	LC-MS	Proteomic profiling of breast cancer tissues can predict tumor response to neoadjuvant chemotherapy.
He et al. 2009 [69]	D & T	primary	52	SELDI-TOF MS	Protein biosignatures were identified as having potential utility in tumor classification and predicting therapeutic responses.
Hulahan et al. 2024 [73]	Diagnosis	P & DCIS	22	Multiplexed spatial proteomics	Eight fibrillar collagen peptides could distinguish with high specificity and sensitivity between ductal carcinoma in situ (DCIS) and invasive breast cancer.
Izani Othman et al. 2009 [74]	Diagnosis	primary	20	LC-MS/MS	82 common and differentially expressed proteins from normal and cancerous breast tissues were identified.
Jeon et al. 2024 [76]	Therapy	primary	56	Mass spectrometry	Coronin-1A and titin were upregulated in the immune-inflamed subtype, and α-1-antitrypsin was upregulated in the immune-excluded/desert subtype.
Johansson et al. 2015 [77]	Therapy	primary	24	nanoLC-MS/MS	High levels of Calcyphosine (CAPS) in tumor tissue are a potential predictive biomarker for tamoxifen resistance in estrogen-receptor-positive breast cancer.
Kang et al. 2010 [79]	Diagnosis	P & DCIS	164	MALDI-TOF MS	RhoGDIα, CAPG, WDR1, CK8 demonstrated potential importance in the tumor microenvironment and invasion processes.
Kim et al. 2009 [82]	Diagnosis	primary	17	MALDI-TOF MS	Upregulation of actin-remodeling proteins, particularly coronin-1A and p34-Arc, in breast cancer is associated with increased cancer invasiveness.

Supplementary Table S2. (continued – part 4).

Study	Setting	BC stage	Number of BC specimens	Primary Proteomic method	Main findings and conclusions
Ku et al. 2025 [155]	Therapy	primary	61	nanoLC-MS/MS	By combining NGS with proteomics correlations between specific gene alterations (FANCA, HRAS, PIK3CA, MAP2K1, JAK2) and the expression of 22 proteins were found, suggesting potential molecular mechanisms underlying breast cancer development and progression.
Lin et al. 2021 [87]	D & T	primary	24	iTRAQ LC-MS/MS	Proteomic data were generated for triple negative breast cancer accurate subtype classification and therapeutic targets research.
Magara et al. 2024 [90]	Therapy	P & DCIS	133	Multilayered proteomics	JAM-A-targeted therapy ideally combined with LAT1-targeted therapy may be a new therapeutic strategy.
Meric-Bernstam et al. 2014 [93]	Diagnosis	primary	53	RPPA	PI3K pathway activation is greater in core needle biopsy compared with postexcision surgical samples, suggesting a potential loss of phosphorylation during surgical manipulation, or with cold ischemia of surgical specimens.
Michaut et al. 2016 [94]	Diagnosis	primary	55	RPPA	Two biologically distinct subtypes of invasive lobular breast cancer were identified.
Moriggi et al. 2018 [96]	Diagnosis	primary	26	2-DE, MALDI-MS	58 overexpressed genes, encoding for 43 extracellular matrix proteins, are associated with structural and metabolic changes in hormone-receptor negative and triple negative BC.
Nakagawa et al. 2006 97]	Diagnosis	primary	65	SELDI-TOF MS	Protein peaks (4,871 Da and 8,596 Da) combined with lymphovascular invasion can predict axillary lymph node metastasis in primary breast cancer.
Neubauer et al. 2008 [98]	Diagnosis	primary	16	Quantitative multiplex proteomics	Cyt-b5, CRABP-II, Neudesin/SPUF and Hemoglobin may explain differences in tamoxifen responsiveness between ER+/PR+ and ER+/PR− breast cancers.
Neubauer et al. 2006 [99]	Diagnosis	primary	24	MALDI-TOF MS	PGRMC1 phosphorylation may be involved in the clinical differences that underpin breast tumors of differing ER status.
Niméus et al. 2007 [100]	D & T	primary	20	MALDI-TOF-TOF MS	Several Proteins were identified to be differentially expressed between breast cancer patients with and without distant recurrences after adjuvant CMF therapy.
Ou et al. 2008 [101]	Diagnosis	primary	63	MALDI-TOF MS	HSP90α, NDRG1, Annexin A1, FASN, TPM4, 6PGL, and CAZ2 are upregulated in breast cancer tissues, while AHSG and 14-3-3 sigma protein are downregulated.
Panis et al. 2013 [102]	Diagnosis	P & M	135	label-free MS	Gelsolin, lumican, clusterin, SALL4, PMS2, hTERT, TNF-$\alpha$and GRHL3 are differentially expressed between early and advanced breast cancer stages.

Supplementary Table S2. (continued – part 5).

Study	Setting	BC stage	Number of BC specimens	Primary Proteomic method	Main findings and conclusions
Pozniak et al. 2016 [107]	Diagnosis	primary	41/25	MS	The comparison between primary tumors and their matched lymph node metastases showed high similarity in protein expression.
Procházková et al. 2017 [108]	Diagnosis	primary	96	mTRAQ labeling (mTRAQ-SRM)	A panel of gene products that can contribute to breast cancer aggressiveness and metastasis was identified.
Pucci-Minafra et al. 2017 [109]	Diagnosis	primary	13	2D gel electrophoresis and MS	Glycolytic enzymes, detox and heat shock proteins, members of annexin and S100 protein families, cathepsin D, DDAH2 and PARK7 were found in all patients.
Pucci-Minafra et al. 2007 [110]	Diagnosis	primary	37	MALDI-TOF	Large-scale proteomics is a valid tool to evaluate on the same assay individual proteins, to be proposed as prognostic indicators and susceptibility to metastasis in breast cancer.
Roberts et al. 2004 [112]	Diagnosis	primary	27	2-DE	Novel insights into the proteomic mapping of ERα, HER2/neu, cytokeratin 7, Bax and p53 in early BC tissue was highlighted.
Rojas et al. 2019 [113]	Diagnosis	P & M	51	PRM targeted proteomics	Three possible biomarkers may predict appearance of CNS metastases in triple negative breast cancer.
Ruckhäberle et al. 2010 [114]	Diagnosis	primary	19	Isobaric TMT label-based proteomics	Isobaric TMT duplex approach allows the quantification of differences in protein expression levels; 18 proteins had significantly different expression between the ER+ & the ER-.
Sanders et al. 2008 [116]	Diagnosis	primary	122	MALDI-TOF MS	S100A6 (calcyclin) and S100A8 (calgranulin A) highlight potential roles in cancer progression, diagnosis, and molecular classification.
Shenoy et al. 2020 [120]	Therapy	primary	113	LC-MS/MS-based proteomic analysis	Two proteins of proline biosynthesis pathway, PYCR1 and ALDH18A1, were significantly associated with resistance to treatment.
Shi et al. 2024 [121]	Therapy	primary	50	MS-Based Label-Free Proteomics	MAGE-D2 appears to be an important therapeutic target of triple negative breast cancer.
Shin et al. 2020 [122]	Diagnosis	P & M	36	Reversed-phase (RP)-nano LC–ESI–MS/MS	TUBB2A was identified as a novel biomarker for the prediction of distant metastases.
Sohn et al. 2013 [124]	Therapy	primary	54	RPPA	AKT, IGFBP2, LKB1, S6 and Stathmin predict relapse-free survival (RFS) in residual triple negative BC patients after neoadjuvant chemotherapy, while PI3K pathway may represent a potential therapeutic target.

Supplementary Table S2. (continued – part 6).

Study	Setting	BC stage	Number of BC specimens	Primary Proteomic method	Main findings and conclusions
Stemke-Hale et al. 2008 [126]	D & T	primary	547	RPPA	Mutations in the PI3K/AKT pathway, particularly in PIK3CA, PTEN, and AKT1, are subtype-specific and may be used as biomarkers and therapeutic targets in breast cancer.
Tamesa et al. 2009 [128]	Diagnosis	primary	40/30	LC-MS/MS	Autoantibodies against cyclophilin A and triosephosphate isomerase could serve as potential biomarkers for breast cancer diagnosis.
Tyanova et al. 2016 [131]	Diagnosis	primary	40	MS analysis	Global profiling of breast cancer clinical samples allows the attribution of biological processes to the different breast cancer subtypes (ER/PR, HER2 positive and TNBC).
Valo et al. 2019 [132]	Diagnosis	P & DCIS	72	SWATH-MS	Circulating OLFM4 could be an interesting biomarker of early breast cancer.
Yang et al. 2016 [138]	Therapy	primary	36	LC-MS/MS	FKBP4 and S100A9 are promising predictive biomarkers for determining drug resistance in breast cancer patients undergoing neoadjuvant chemotherapy.
Yang et al. 2015 [139]	Diagnosis	primary	60	LC-MS/MS	TfR levels in breast tissue can be measured precisely with LC-MS/MS and could possibly improve the diagnosis of breast cancer and assessment of drug resistance.
Yang et al. 2014 [140]	Diagnosis	primary	36	LC-MS/MS	The level of FR isoforms was associated with several histopathological features and molecular subtypes.
Yang et al. 2012 [141]	Therapy	P & M	83	LC-MS/MS	ERK/Bcl-2-mediated anti-apoptosis was investigated in general and in the development of drug resistance.
Yanovich et al. 2018 [142]	Diagnosis	primary	109	LC-MS	A novel luminal subtype characterized by increased PI3K signaling has been identified.
Zeng et al. 2017 [145]	Diagnosis	primary	23/23	Quantitative iTRAQ	NUCB2 can be used as a potential biomarker for breast cancer metastasis and a prognostic predictor of breast cancer patients.
Zhang et al. 2008 [149]	Diagnosis	primary	94	MALDI-TOF-TOF MS	CK19 in HER-2+ breast cancer is associated with tumor aggressiveness, suggesting CK19's potential role as a biomarker for identifying more aggressive breast cancer subtypes.
Zhang et al. 2005 [150]	Diagnosis	primary	25	MALDI-TOF	HnRNP H1, RKIP, and GRP78 are linked to the aggressive phenotype of HER-2/neu + breast cancer.
Zhong et al. 2018 [153]	Diagnosis	primary	54/54	iTRAQ	Protein S100-A8 may be associated with lymph nodes metastasis of breast cancer and may be a marker for progression of breast cancer.

BC = breast cancer; D = Diagnosis; DCIS = Ductal carcinoma in situ; ER = estrogen receptor(s); HPLC = High-Performance Liquid Chromatography; iTRAQ = Isobaric Tag for Relative and Absolute Quantification; LC/ESI-MS/MS = Liquid Chromatography, Electrospray Ionization, Mass spectrometry; LC-MS/MS = Liquid chromatography-tandem mass spectrometry; M = Metastasis; MALDI-TOF = Matrix-assisted laser desorption/ionization-time of flight; MS = Mass spectrometry; n.a. = not available; nano-LC-MS/MS = nanoscale liquid chromatography-tandem mass spectrometry; P = Primary; PR = progesterone receptor(s); PRM = Parallel reaction monitoring; RPLC = Reversed-phase liquid chromatography;RPPA = Reverse Phase Protein Arrays; SDS-PAGE = Sodium dodecyl sulfate polyacrylamide gel electrophoresis; SELDI-TOF = Surface-Enhanced Laser Desorption/Ionization Time-of-Flight; SRM-MS = Selected Reaction Monitoring Mass Spectrometry; SWATH = Sequential Window Acquisition of all Theoretical Mass Spectra; T = Therapy; TMT = Tandem Mass Tag; TNBC = triple negative breast cancer; 2D-PAGE = Two-dimensional polyacrylamide gel electrophoresis.

Supplementary Table S3. Main findings and conclusions of proteomic studies in plasma and serum from breast cancer patients.

Study	Setting	BC stage	Type of specimen	Number of BC specimens	Primary Proteomic method	Main findings and conclusions
Alvarez et al. 2022 [19]	Therapy	primary	Plasma Evs	17	PPLC and LC-MS/MS	PPLC-isolated BEVs and five associated proteins may serve to identify non-responders to spare them the toxic effects of neoadjuvant chemotherapy.
An et al. 2022 [21]	Diagnosis	primary	Plasma	107	Nano-LC-MS/MS	A panel of 47 plasma metabolites, including sphingomyelins, glutamate, and cysteine could be potential diagnostic biomarkers for breast cancer.
Belluco et al. 2007 [26]	Diagnosis	primary	Serum	155	SELDI-TOF MS	A proteomic pattern consisting of 7 low-molecular-weight ion peaks is a highly sensitive and specific method for early detection of stage 1 breast cancer.
Bera et al. 2020 [27]	Diagnosis	primary	Serum	240	Antibody Microarrays and MSD Multi-array	The epigenetic regulation of inflammatory processes plays a critical role in breast cancer recurrence and identified proteins could lead to the development of a serum-based breast cancer recurrence assay.
Corrêa et al. 2017 [41]	Diagnosis	primary	Plasma	107	Nano-LC-MS/MS	The plasma proteomic profile of breast cancer subtypes was determined.
Dalenc et al. 2010 [43]	Therapy	metastatic	Serum	57	SELDI-TOF MS	Fibrinogen α peptide could serve as a predictive biomarker for therapeutic response in ER+ breast cancer patients undergoing the tipifarnib and tamoxifen combination therapy.
Drukier et al. 2006 [46]	Diagnosis	primary		264	IA/MPD	Ultrasensitive, multi-biomarker immunoassays significantly improve early breast cancer detection accuracy.
Fernandez-Pol et al. 2005 [48]	D & T	P & M	Serum	243	HPLC	MPS-1 is useful for early detection, monitoring, and management of breast cancer, superior than CA-15-3 and CEA.
Fredolini et al. 2020 [50]	Diagnosis	primary	Serum	20	Affinity hydrogel nanoparticles coupled with LC-MS/MS	A highly specific and sensitive protein signature indicative of early-stage breast cancer was identified and verified.
Gajbhiye et al. 2017 [51]	Diagnosis	primary	Serum	76	2D-DIGE, iTRAQ and SWATH-MS	Serum proteome alterations may help to distinguish breast cancer subtypes (luminal A and, B, HER2-positive and triple negative breast cancer).
Garisi, Tommasi et al. 2012 [57]	Diagnosis	primary	Serum	192	SELDI-TOF MS	The serum profile of familial breast cancer patients was different when compared with that of sporadic breast cancer patients.

Supplementary Table S3. (continued – part 2).

Study	Setting	BC stage	Type of specimen	Number of BC specimens	Primary Proteomic method	Main findings and conclusions
Garisi, Tufaro et al. 2012 [58]	Diagnosis	primary	Serum	138	SELDI-TOF MS	Normal weight women have a significantly higher probability of having a smaller breast tumor at time of diagnosis and negative axillary lymph nodes. Increased BMI is associated with an altered protein profile in BC patients.
Gast et al. 2011 [59]	Diagnosis	primary	Serum	82	SELDI-TOF MS	Four protein peaks were identified as having significant prognostic value for relapse-free survival (RFS) in high-risk primary breast cancer patients.
Goncalves et al. 2006 [61]	Diagnosis	primary	Serum	81	SELDI-TOF MS	A multiprotein signature, including haptoglobin, transferrin, and apolipoproteins predicts metastatic relapse in high-risk early BC patients.
Grassmann et al. 2024 [64]	Diagnosis	primary	Plasma	796	Proximity Extension Assay	The studied plasma proteins are unlikely to offer additional benefits for risk prediction of short-term overall breast cancer risk.
Henderson et al. 2019 [70]	Diagnosis	P & DCIS	Serum	123	Modified ECL based ELISA	Serum biomarkers provide clinicians with additional information for patients with indeterminate breast imaging results, potentially reducing false-positive breast biopsies.
Henderson et al. 2016 [71]	Diagnosis	P & DCIS	Serum	100	modified ELISA	SPB and TAAb combinatorial protein biomarker assays may aid in the detection of early BC and guide decisions between imaging and tissue biopsy.
Hu et al. 2005 [72]	Diagnosis	P & M	Serum	49	SELDI-TOF MS	SELDI-TOF-MS combined with bioinformatics tools is a promising approach for the early detection of breast cancer, identifying four candidate biomarkers.
Jordan et al. 2020 [77]	D & T	primary	Plasma Evs	20	MS	Circulating EVs from young breast cancer patients could serve as cancer biomarkers, and potential targets for individualized cancer treatment.
Kaur et al. 2024 [79]	Diagnosis	P & M	Serum	73	MS	A set of proteins that could be involved in breast cancer progression in serum was identified.
Kim et al. 2019 [81]	Diagnosis	primary	Plasma	575	MRM MS	Three specific peptides can be a useful tool for breast cancer screening and its accuracy is cancer-type specific.

Supplementary Table S3. (continued – part 3).

Study	Setting	BC stage	Type of specimen	Number of BC specimens	Primary Proteomic method	Main findings and conclusions
Le Naour et al. 2001 [83]	Diagnosis	primary	Serum	30	MALDI-TOF	RS/DJ-1 is a novel circulating tumor antigen eliciting a humoral immune response in breast cancer patients and can be used for early detection and monitoring of breast cancer.
Li et al. 2024 [85]	Therapy	primary	Plasma	40	MS	Specific plasma proteins act as predictive biomarkers of response to immunotherapy.
Lötsch et al. 2022 [87]	Diagnosis	primary	Serum	27	PEA	Serum proteomics markers associated with the development of neuropathic pain after an intraoperative nerve lesion were identified.
Lourenco et al. 2017 [88]	Diagnosis	primary	Serum	26	modified ELISA	Serum biomarkers can effectively detect breast cancer when used in conjunction with imaging and can substantially reduce unnecessary medical procedures.
Majidzadeh-A et al. 2013 [90]	Therapy	primary	Serum	10	MALDI-TOF	CLU, SAA, TTR, ApoE, ApoA-IV, SCCA2, Fibrinogen b chain, C4b-BP a chain may contribute to tamoxifen resistance mechanisms.
Minton et al. 2013 [94]	Diagnosis	primary	Serum	45	SELDI-TOF MS	SAA, Colectin, IgG KLC, C1q hold a potential role in immune dysregulation and sustained inflammation in Cancer Related Fatigue Syndrome.
Pires et al. 2019 [106]	Therapy	primary	Plasma	200	MS	The connection between inflammation, the complement and oxidative stress seems to be a pivotal axis in chemoresistance of luminal A breast cancer.
Riley et al. 2011 [111]	Diagnosis	P & DCIS	Serum	216	LC-MS/MS	A LC-MS proteomics dataset has been generated that includes more than 800 discrete human plasma profiles.
Rui et al. 2003 [113]	Diagnosis	primary	Serum	145	MALDI-TOF	HSP27 and 14-3-3 sigma are key biomarkers in distinguishing breast cancer from non-cancer conditions.
Santana et al. 2024 [117]	Diagnosis	primary	Plasma	143	LC-MS/MS	The HDL proteome showed discriminatory abilities across different clinical stages of breast cancer and a distinct profile in triple negative breast cancer.
Schaub et al. 2009 [119]	Diagnosis	P, M & DCIS	Serum	125	MALDI-TOF MS	Several differentially expressed proteins and peptides associated with BC staging, nodal status, and obesity were identified.

Supplementary Table S3. (continued – part 4).

Study	Setting	BC stage	Type of specimen	Number of BC specimens	Primary Proteomic method	Main findings and conclusions
Sinha et al. 2023 [123]	Diagnosis	primary	Saliva and Serum	15	iTRAQ analysis	Several candidate biomarkers for the early detection of breast cancer in serum and saliva were identified.
Starodubtseva et al. 2023 [125]	Diagnosis	metastatic	Serum	25	LC-MRM MS	Distinguishing between metastatic and non-metastatic breast cancer based on proteomic features had 90% accuracy (and 80% for lipidomic).
Suman et al. 2016 [127]	Diagnosis	primary	Plasma	32	iTRAQ analysis	Four proteins (FN1, A2M, C4BPA and CFB) had strong association with molecular subtypes of breast cancer.
Tomar et al. [154]	Diagnosis	P & M	Serum	12	MS	Two proteins, SBSN and PFN1, were progressively overexpressed and underexpressed, respectively, in breast cancer compared to control samples, ranging from early-stage to metastatic cases.
Tutanova et al. 2020 [130]	Diagnosis	primary	Plasma, WBE	23	MS	Total blood exosomes contain more malignant neoplasm-associated proteins than plasma exosomes.
Vinik et al. 2020 [133]	Diagnosis	primary	Plasma Evs	52	RPPA	Several potential markers that could contribute to early detection of BC were identified
Xu et al. 2015 [135]	Diagnosis	primary	Serum	60	LC-MS/MS	SISCAPA-targeted proteomics alllowed quatification of low-abundant serum transferrin receptor in breast cancer patients pre- and post-chemotherapy.
Xu et al. 2024 [134]	Diagnosis	primary	Serum Evs	126	MS	Proteins carried by breast cancer–derived EVs could be used as minimally invasive liquid biopsy tool for the early detection of breast cancer and for discriminating lymph node involvement and distant metastasis.
Yan et al. 2022 [136]	Diagnosis	primary	Serum	64	MB-IMAC-Cu and MALDI-TOF MS	APOC1 could serve as a candidate serum diagnostic biomarker for breast cancer.
Yang et al. 2020 [137]	Therapy	primary	Serum	51	Isobaric TMT label-based quantitative proteomics	A serum-based protein signature that potentially predicts the therapeutic effects of trastuzumab-based therapy for HER2-positive breast cancer patients was developed.
Ye et al. 2024 [143]	Diagnosis	P & M	Plasma	51	UPLC-MS/MS	Distinct dysregulations in plasma proteins and metabolites among breast cancer patients with bone, liver, and lung metastases were identified.

Supplementary Table S3. (continued – part 5).

Study	Setting	BC stage	Type of specimen	Number of BC specimens	Primary Proteomic method	Main findings and conclusions
Zeidan et al. 2018 [144]	Diagnosis	primary	Serum	399	LC-MS/MS	Higher circulating resistin correlated with node-negative patients and longer DFS independent of BMI and ER status in women with early onset BC.
Zhang et al. 2013 [148]	Diagnosis	P & DCIS	Serum	100	LC-MS/MS	Feed Forward Neural Network (FFNN) enhances the development of more accurate and reliable biomarker panels for the early diagnosis of breast cancer.
Zhang et al. 2015 [149]	Diagnosis	primary	Serum	80	LC-MS/MS	Pathway-based biomarkers can significantly enhance the early detection and diagnostic accuracy of breast cancer.
Zhang et al. 2013 [150]	Diagnosis	primary	Plasma	80	LC-ESI-MS/MS	Identification of eight alternative splicing isoform biomarkers can assist the early diagnosis of breast cancer.
Zhao et al. 2024 [152]	Diagnosis	primary	Plasma	10	LC-MS/MS	Proteomic signatures of patients with luminal-A BC, multiple differential proteins, and three plasma proteins as potential diagnostic biomarkers for breast cancer were identified.

BC = breast cancer; ER = estrogen resceptor(s); EVs = extracellular vesicles; iTRAQ = Isobaric Tag for Relative and Absolute Quantification; LC = Liquid chromatography**;** LC-ESI-MS/MS = Liquid Chromatography-Electrospray Ionization-Tandem Mass Spectrometry; LC-MS/MS = Liquid chromatography-tandem mass spectrometry**;** MALDI-TOF = Matrix-assisted laser desorption/ionization-time of flight; MB-IMAC = Magnetic beads based immobilized metal ion affinity chromatography; MRM = Multiple reaction monitoring; MS = Mass spectrometry; PEA = proximity extension assay; PPLC = Particle purification liquid chromatography; RPPA = Reverse Phase Protein Arrays; SELDI-TOF = Surface-Enhanced Laser Desorption/Ionization Time-of-Flight; SWATH = Sequential Window Acquisition of all Theoretical Mass Spectra; TMT = Tandem Mass Tag; 2D-DIGE = two-dimensional difference gel electrophoresis; UPLC = Ultra-performance liquid chromatography; WBE = whole blood exosomes.

Supplementary Table S4. Main findings and conclusions of proteomic studies in nipple aspiration fluid (NAF), urine, saliva, tear fluid, pleural effusions, tumor interstitial fluid and lymph nodes from breast cancer patients.

Study	Setting	BC stage	Type of specimen	Number of BC specimens	Primary proteomic Method	Main findings and conclusions
Brunoro et al. 2019 [35]	Diagnosis	primary	NAF	10	MS	Three proteins were found at higher levels in nipple aspiration fluid of breast cancer patients.
Kuerer et al. 2004 [82]	Diagnosis	primary	NAF	23	SELDI-TOF MS	Differential protein expression in NAF is correlated with presence and extent of axillary lymph node metastases in early breast cancer patients.
Paweletz et al. 2001 [104]	Diagnosis	primary	NAF	12	SELDI-TOF MS	Distinct LMW protein peaks at NAF analysis, including those at 4233.09 Da and 9470.0 Da, could differentiate breast cancer from normal conditions.
Pawlik et al. 2006 [105]	Diagnosis	primary	NAF	18	LC-MS/MS	Several proteins, notably vitamin D-binding protein, were found to be differentially expressed in NAF between BC tissues and normal breasts.
Sauter et al. 2002 [118]	Diagnosis	primary	NAF	20	SELDI-TOF MS	Five protein peaks were found to be differentially expressed in NAF analysis between breast cancer and normal indivuduals.
Beretov et al. 2015 [28]	Diagnosis	P & DCIS	Urine	20	LC-MS/MS	Urine is a useful non-invasive source of biomarkers that have potential for clinical use in the detection of breast cancer.
Gajbhiye et al. 2016 [52]	Diagnosis	primary	Urine	43	2D-DIGE, iTRAQ, SWATH MS	A panel of noninvasive candidate urinary protein markers for HER2-enriched breast cancer BC was identified.
Jeanmard et al. 2023 [74]	Diagnosis	primary	Urinary EVs	47	LC-MS/MS	Urinary extracellular vesicle proteins may be used as screening biomarkers for breast cancer.
Giri et al. 2022 [60]	Diagnosis	metastatic	Saliva	20	PRM-MS	Three salivary proteins successfully differentiated triple negative breast cancer patients from healthy subjects with 80% sensitivity and 95% specificity.
Zhang et al. 2010 [151]	Diagnosis	primary	saliva	40	MALDI-TOF	Carbonic Anhydrase VI, Psoriasin and transcriptomic biomarkers are useful for early breast cancer detection.
Sinha et al. 2023 [123]	Diagnosis	primary	Saliva, Serum	15	iTRAQ proteomic analysis	Several candidate biomarkers for the early detection of breast cancer in serum and saliva were identified.

Supplementary Table 4 (continued)

Study	Setting	BC stage	Type of specimen	Number of BC specimens	Primary proteomic Method	Main findings and conclusions
Böhm et al. 2012 [31]	Diagnosis	primary	Tear fluid	25	MALDI-TOF	Proteomic analysis of tear fluid may contribute to early breast cancer detection.
Lebrecht et al. 2009 [84]	Diagnosis	primary	Tear fluid	50	SELDI-TOF MS	Proteomic profiling of tear fluid identified a biomarker panel with potential utility in breast cancer diagnosis.
Mayayo-Peralta et al. 2024 [91]	Therapy	metastatic	Pleural effusions	47	Phosphoproteomics analysis	Evidence for decreased activity of several key kinases in ERα-converted metastases was found.
Terkelsen et al. 2020 [129]	Diagnosis	primary	TIF	35	LC-MS/MS	Ten proteins, AGR3, BCAM, CELSR1, MIEN1, NAT1, PIP4K2B, SEC23B, THTPA, TMEM51, and ULBP2 stratify the tumor subtype-specific TIFs.
Pathania et al. 2022 [103]	Diagnosis	primary	SLN	13	iTRAQ proteomic analysis & MS	Extracellular Matrix proteins may be used to assess the status of sentinel lymph nodes to guide surgical intervention in early breast cancer.
Pozniak et al. 2016 [107]	Diagnosis	primary	BC, LN	41+25	MS	The comparison between primary tumors and their matched lymph node metastases showed high similarity in protein expression.
Zeng et al. 2017 [145]	Diagnosis	primary	BC, LN	23+23	iTRAQ proteomic analysis	NUCB2 can be used as a potential biomarker for breast cancer metastasis and a prognostic predictor of breast cancer patients.
Zhong et al. 2018 [153]	Diagnosis	primary	BC, LN	54+54	iTRAQ proteomic analysis	Protein S100-A8 may be associated with lymph nodes metastasis of breast cancer and be a marker for breast cancer progression.

BC = breast cancer; EVs = extracellular vesicles; TIF = Tumor interstitial fluid; IHC = Immunohistochemistry; iTRAQ = Isobaric Tag for Relative and Absolute Quantification; LC-MS/MS = Liquid chromatography-tandem mass spectrometry; LN = Lymph nodes; MALDI-TOF = Matrix-assisted laser desorption/ionization-time of flight; MS = Mass spectrometry; NAF = Nipple aspiration fluid; PRM = Parallel reaction monitoring; SELDI-TOF = Surface-Enhanced Laser Desorption/Ionization Time-of-Flight; SLN = Sentinel lymph nodes; SWATH = Sequential Window Acquisition of all Theoretical Mass Spectra; 2D-DIGE = two-dimensional difference gel electrophoresis.

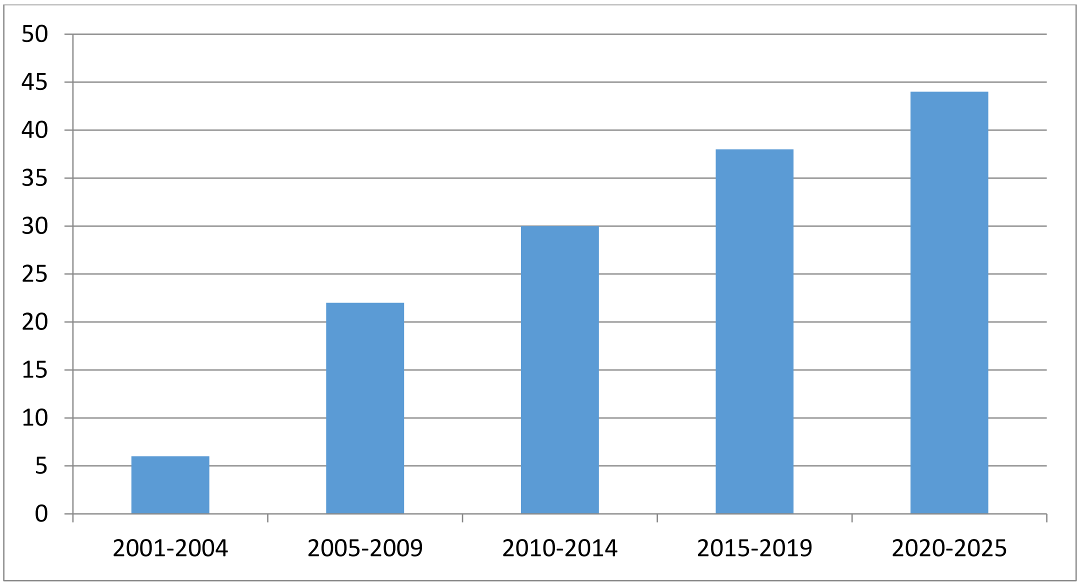

Supplementary Figure S1: Number of studies according to the year of publication.

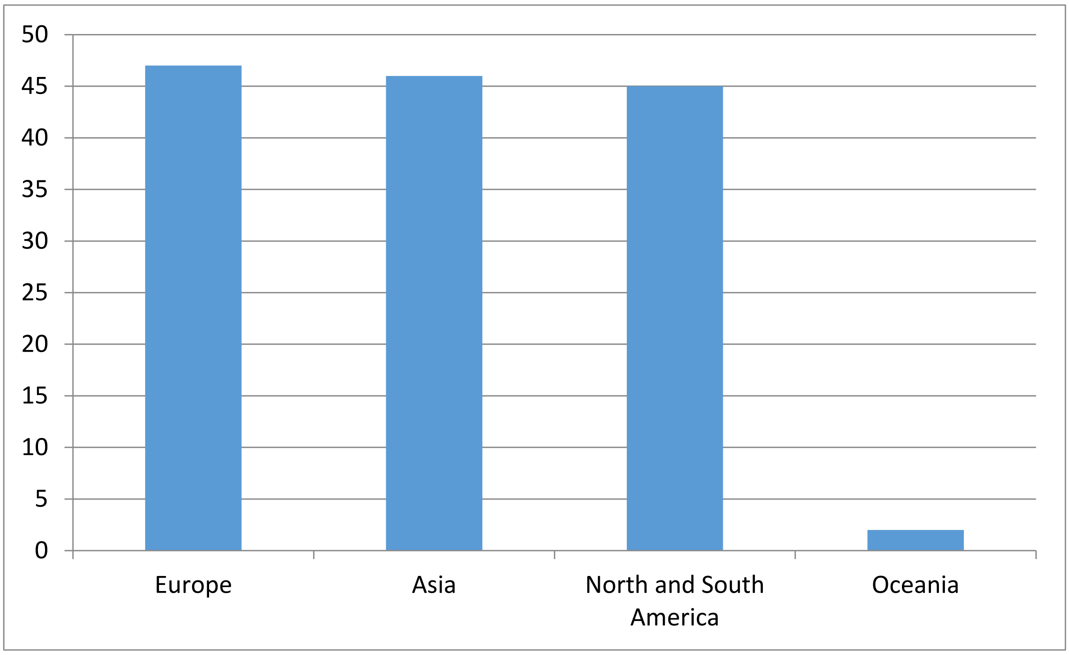

Supplementary Figure S2: Number of studies according to geographical area.

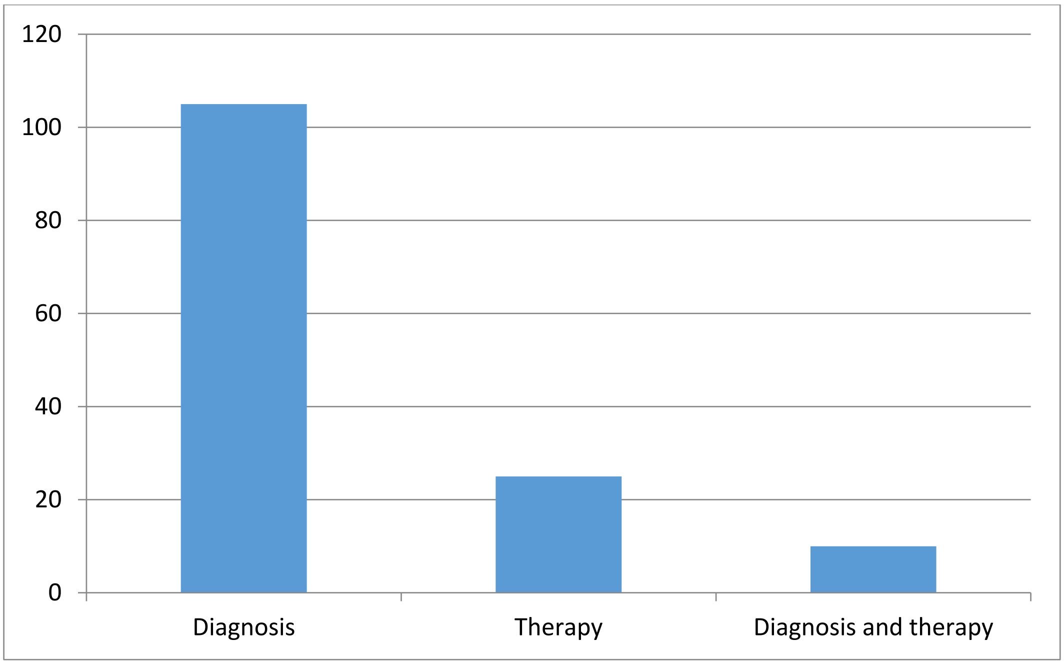

Supplementary Figure S3: Number of studies according to clinical relevance.

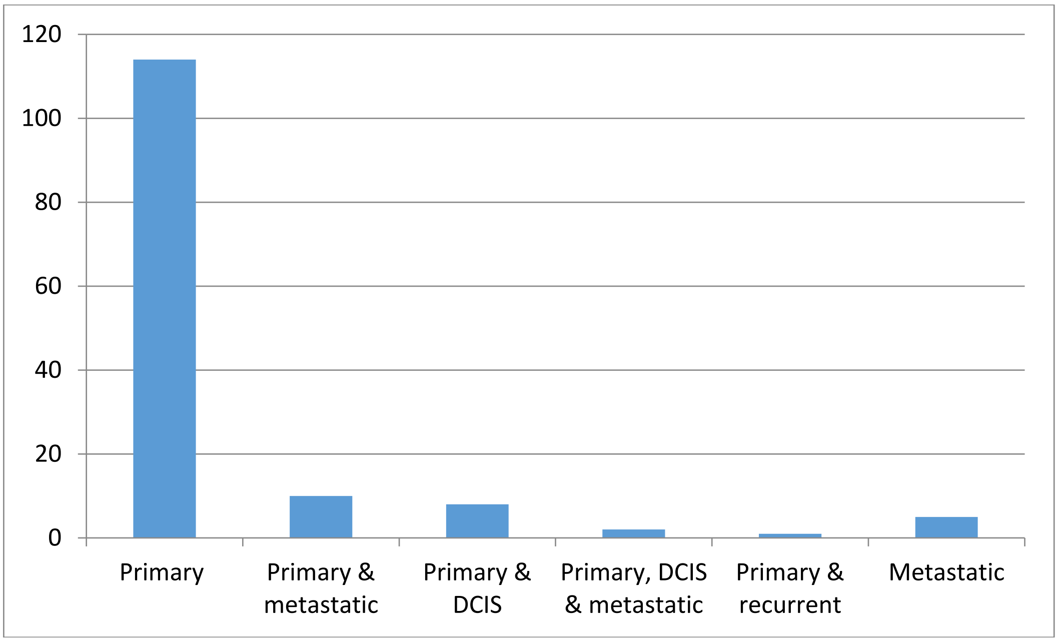

Supplementary Figure S4: Number of studies according to disease stage of analyzed clinical specimens.

Supplementary File S1: List of In Vitro Studies

1. Adam PJ, Boyd R, Tyson KL, Fletcher GC, Stamps A, Hudson L, Poyser HR, Redpath N, Griffiths M, Steers G, Harris AL, Patel S, Berry J, Loader JA, Townsend RR, Daviet L, Legrain P, Parekh R, Terrett JA. Comprehensive proteomic analysis of breast cancer cell membranes reveals unique proteins with potential roles in clinical cancer. J Biol Chem 2003; 278: 6482-6489. doi: 10.1074/jbc.M210184200.
2. Ahuja S, Lazar IM. Proteomic insights into breast cancer response to brain cell-secreted factors. Sci Rep 2024; 14: 19351. doi: 10.1038/s41598-024-70386-7.
3. Aka JA, Lin SX.. Comparison of functional proteomic analyses of human breast cancer cell lines T47D and MCF7. PLoS One 2012; 7: e31532.doi: 10.1371/journal.pone.0031532.
4. Akekawatchai C, Roytrakul S, Phaonakrop N, Jaresitthikunchai J, Jitrapakdee S. Proteomic Analysis of the Anoikis-Resistant Human Breast Cancer Cell Lines. Methods Mol Biol 2020:2138:185-193. doi: 10.1007/978-1-0716-0471-7_11.
5. Akhtar U, Khurshid Y, El-Aarag B, Syed B, Khan IA, Parang K, Ahmed A. Proteomic characterization and cytotoxic potential of proteins from Cuscuta (Cuscuta epithymum (L.) crude herbal product against MCF-7 human breast cancer cell line. BMC Complement Med Ther 2024; 24: 195. doi: 10.1186/s12906-024-04495-1.
6. Alexandrova E, Giurato G, Saggese P, Pecoraro G, Lamberti J, Ravo M, Rizzo F, Rocco D, Tarallo R, Nyman TA, Collina F, Cantile M, Di Bonito M, Botti G, Nassa G, Weisz A. Interaction Proteomics Identifies ERbeta Association with Chromatin Repressive Complexes to Inhibit Cholesterol Biosynthesis and Exert An Oncosuppressive Role in Triple-negative Breast Cancer. Mol Cell Proteomics 2020; 19: 245-260. doi: 10.1074/mcp.RA119.001817.
7. Al-Zaidan L, El Ruz RA, Malki AM. Screening Novel Molecular Targets of Metformin in Breast Cancer by Proteomic Approach. Front Public Health 2017: 5: 277. doi: 10.3389/fpubh.2017.00277.
8. Antwi K, Hanavan PD, Myers CE, Ruiz YW, Thompson EJ, Lake DF. Proteomic identification of an MHC-binding peptidome from pancreas and breast cancer cell lines. Mol Immunol2009; 46: 2931-2937. doi: 10.1016/j.molimm.2009.06.021.
9. Balasubramani M, Nakao C, Uechi GT, Cardamone J, Kamath K, Leslie KL, Balachandran R, Wilson L, Day BW, Jordan MA. Characterization and detection of cellular and proteomic alterations in stable stathmin-overexpressing, taxol-resistant BT549 breast cancer cells using offgelIEF/PAGE difference gel electrophoresis. Mutat Res 2011; 722: 154-164.doi: 10.1016/j.mrgentox.2010.08.019.
10. Balsa LM, Rodriguez MR, Ferraresi-Curotto V, Parajón-Costa BS, Gonzalez-Baró AC, León IE.Finding New Molecular Targets of Two Copper(II)-Hydrazone Complexes on Triple-Negative Breast Cancer Cells Using Mass-Spectrometry-Based Quantitative Proteomics. Int J Mol Sci 2023; 24: 7531. doi: 10.3390/ijms24087531.
11. Barneh F, Salimi M, Goshadrou F, Ashtiani M, Mirzaie M, Zali H, Jafari M. Valproic acid inhibits the protective effects of stromal cells against chemotherapy in breast cancer: Insights from proteomics and systems biology. J Cell Biochem 2018; 119: 9270-9283. doi: 10.1002/jcb.27196.
12. Bateman NW, Sun M, Hood BL, Flint MS, Conrads TP. Defining central themes in breast cancer biology by differential proteomics: conserved regulation of cell spreading and focal adhesion kinase. J Proteome Res 2010; 9: 5311-5324. doi: 10.1021/pr100580e.
13. Bernhardt S, Tönsing C, Mitra D, Erdem N, Müller-Decker K, Korf U, Kreutz C, Timmer J, Wiemann S. Functional Proteomics of Breast Cancer Metabolism Identifies GLUL as Responder during Hypoxic Adaptation. J Proteome Res 2019; 18: 1352-1362. doi: 10.1021/acs.jproteome.8b00944.

14. Beykou M, Arias-Garcia M, Roumeliotis TI, Choudhary JS, Moser N, Georgiou P, Bakal C. Proteomic characterisation of triple negative breast cancer cells following CDK4/6 inhibition. Sci Data 2022; 9: 395. doi: 10.1038/s41597-022-01512-1.
15. Bober P, Alexovic M, Talian I, Tomkova Z, Viscorova Z, Benckova M, Andrasina I, Ciccocioppo R, Petrovic D, Adamek M, Kruzliak P, Sabo J. Proteomic analysis of the vitamin C effect on the doxorubicin cytotoxicity in the MCF-7 breast cancer cell line. J Cancer Res Clin Oncol 2017; 143: 35-42. doi: 10.1007/s00432-016-2259-4.
16. Boyd ZS, Wu QJ, O'Brien C, Spoerke J, Savage H, Fielder PJ, Amler L, Yan Y, Lackner MR. Proteomic analysis of breast cancer molecular subtypes and biomarkers of response to targeted kinase inhibitors using reverse-phase protein microarrays. Mol Cancer Ther 2008; 7: 3695-3706.doi: 10.1158/1535-7163.MCT-08-0810.
17. Boyer AP, Collier TS, Vidavsky I, Bose R. Quantitative proteomics with siRNA screening identifies novel mechanisms of trastuzumab resistance in HER2 amplified breast cancers. Mol Cell Proteomics 2013; 12: 180-193. doi: 10.1074/mcp.M112.020115.
18. Braakman RB, Luider TM, Martens JW, Foekens JA, Umar A. Laser capture microdissection applications in breast cancer proteomics. Methods Mol Biol 2011: 755: 143-54. doi: 10.1007/978-1-61779-163-5_11.
19. Braga FHG, Gómez-Mendoza DP, Lemos RP, Rodrigues-Ribeiro L, Raíssa-Oliveira B, Rodrigues ALP, Gorshkov V, Kjeldsen F, Cruz JS, Verano-Braga T. Proteomic analysis reveals stage-specific reprogramed metabolism for the primary breast cancer cell lines MGSO-3 and MACL-1. Proteomics 2022; 22: e2200095. doi: 10.1002/pmic.202200095.
20. Brauer BL, Wiredu K, Gerber SA, Kettenbach AN. Evaluation of Quantification and Normalization Strategies for Phosphoprotein Phosphatase Affinity Proteomics: Application to Breast Cancer Signaling. J Proteome Res 2023; 22: 47-61. doi: 10.1021/acs.jproteome.2c00465.
21. Brown KJ, Fenselau C. Investigation of doxorubicin resistance in MCF-7 breast cancer cells using shot-gun comparative proteomics with proteolytic 18O labeling. J Proteome Res 2004; 3: 455-462. doi: 10.1021/pr0340835.
22. Cai J, Chen S, Zhang W, Wei Y, Lu J, Xing J, Dong Y. Proteomic analysis of differentially expressed proteins in 5-fluorouracil-treated human breast cancer MCF-7 cells. Clin Transl Oncol 2014; 16: 650-659. doi: 10.1007/s12094-013-1127-9.
23. Calderón-González KG, Valero Rustarazo ML, Labra-Barrios ML, Bazán-Méndez CI, Tavera-Tapia A, Herrera-Aguirre ME, Sánchez del Pino MM, Gallegos-Pérez JL, González-Márquez H, Hernández-Hernández JM, León-Ávila G, Rodríguez-Cuevas S, Guisa-Hohenstein F, Luna-Arias JP.Determination of the protein expression profiles of breast cancer cell lines by quantitative proteomics using iTRAQ labelling and tandem mass spectrometry. J Proteomics 2015: 124: 50-78.doi: 10.1016/j.jprot.2015.04.018.
24. Canelle L, Bousquet J, Pionneau C, Hardouin J, Choquet-Kastylevsky G, Joubert-Caron R, Caron M. A proteomic approach to investigatepotential biomarkers directed against membrane-associated breast cancer proteins. Electrophoresis 2006; 27: 1609-1616.doi: 10.1002/elps.200500712.
25. Cao L, Zhou Y, Li X, Lin S, Tan Z, Guan F. Integrating transcriptomics, proteomics, glycomics and glycoproteomics to characterize paclitaxel resistance in breast cancer cells. J Proteomics 2021: 243: 104266. doi: 10.1016/j.jprot.2021.104266.
26. Caruso JA, Stemmer PM. Proteomic profiling of lipid rafts in a human breast cancer model of tumorigenic progression. Clin Exp Metastasis 2011; 28: 529-540.δoi: 10.1007/s10585-011-9389-5.
27. Cha HK, Cheon S, Kim H, Lee KM, Ryu HS, Han D. Discovery of Proteins Responsible for Resistance to Three Chemotherapy Drugs in Breast Cancer Cells Using Proteomics and Bioinformatics Analysis. Molecules 2022; 27: 1762. doi: 10.3390/molecules27061762.
28. Chang HY, Li MH, Huang TC, Hsu CL, Tsai SR, Lee SC, Huang HC, Juan HF. Quantitative proteomics reveals middle infrared radiation-interfered networks in breast cancer cells. J Proteome Res 2015; 14: 1250-1262.doi: 10.1021/pr5011873.

29. Chao T, Ladd JJ, Qiu J, Johnson MM, Israel R, Chin A, Wang H, Prentice RL, Feng Z, Disis ML, Hanash S. Proteomic profiling of the autoimmune response to breast cancer antigens uncovers a suppressive effect of hormone therapy. Proteomics Clin Appl 2013; 7: 327-336. doi: 10.1002/prca.201200058.
30. Chen S, Cai J, Zhang W, Zheng X, Hu S, Lu J, Xing J, Dong Y. Proteomic identification of differentially expressed proteins associated with the multiple drug resistance in methotrexate-resistant human breast cancer cells. Int J Oncol 2014; 45: 448-458. doi: 10.3892/ijo.2014.2389.
31. Chen S, Dong Q, Hu S, Cai J, Zhang W, Sun J, Wang T, Xie J, He H, Xing J, Lu J, Dong Y. Proteomic analysis of the proteins that are associated with the resistance to paclitaxel in human breast cancer cells. Mol Biosyst 2014; 10: 294-303. doi: 10.1039/c3mb70428a.
32. Chen ST, Pan TL, Tsai YC, Huang CM. Proteomics reveals protein profile changes in doxorubicin--treated MCF-7 human breast cancer cells. Cancer Lett 2002; 181: 95-107. doi: 10.1016/s0304-3835(02)00025-3.
33. Chen YW, Chou HC, Lyu PC, Yin HS, Huang FL, Chang WS, Fan CY, Tu IF, Lai TC, Lin ST, Lu YC, Wu CL, Huang SH, Chan HL. Mitochondrial proteomics analysis of tumorigenic and metastatic breast cancer markers. Funct Integr Genomics 2011; 11: 225-239. doi: 10.1007/s10142-011-0210-y.
34. Chesor M, Roytrakul S, Graidist P, Kanokwiroon K.Proteomics analysis of siRNA-mediated silencing of Wilms' tumor 1 in the MDA-MB-468 breast cancer cell line. Oncol Rep 2014; 31: 1754-1760. doi: 10.3892/or.2014.3013.
35. Chou HC, Lu YC, Cheng CS, Chen YW, Lyu PC, Lin CW, Timms JF, Chan HL. Proteomic and redox-proteomic analysis of berberine-induced cytotoxicity in breast cancer cells. J Proteomics 2012; 75: 3158-3176. doi: 10.1016/j.jprot.2012.03.010.
36. Chuthapisith S, Layfield R, Kerr ID, Hughes C, Eremin O. Proteomic profiling of MCF-7 breast cancer cells with chemoresistance to different types of anti-cancer drugs. Int J Oncol 2007; 30: 1545-1551.
37. Chutoe C, Inson I, Krobthong S, Phueakphud N, Khunluck T, Wongtrakoongate P, Charoenphandhu N, Lertsuwan K. Combinatorial effects of cannabinoid receptor 1 and 2 agonists on characteristics and proteomic alteration in MDA-MB-231 breast cancer cells. PLoS One 2024; 19: e0312851. doi: 10.1371/journal.pone.0312851.
38. Cirillo F, Nassa G, Tarallo R, Stellato C, De Filippo MR, Ambrosino C, Baumann M, Nyman TA, Weisz A. Molecular mechanisms of selective estrogen receptor modulator activity in human breast cancer cells: identification of novel nuclear cofactors of antiestrogen-ERα complexes by interaction proteomics. J Proteome Res 2013; 12: 421-431. doi: 10.1021/pr300753u.
39. Clark DJ, Fondrie WE, Liao Z, Hanson PI, Fulton A, Mao L, Yang AJ. Redefining the Breast Cancer Exosome Proteome by Tandem Mass Tag Quantitative Proteomics and Multivariate Cluster Analysis. Anal Chem 2015; 87: 10462-10469. doi: 10.1021/acs.analchem.5b02586.
40. Clulow JA, Storck EM, Lanyon-Hogg T, Kalesh KA, Jones LH, Tate EW. Competition-based, quantitative chemical proteomics in breast cancer cells identifies new target profiles for sulforaphane. Chem Commun (Camb) 2017 May 4;53(37):5182-5185. doi: 10.1039/c6cc08797c.
41. Coló GP, Schweitzer K, Oresti GM, Alonso EG, Chávez LF, Mascaró M, Giorgi G, Curino AC, Facchinetti MM. Proteomic analysis of the effect of hemin in breast cancer. Sci Rep 2023; 13: 10091. doi: 10.1038/s41598-023-35125-4.
42. Costanzo M, Fiocchetti M, Ascenzi P, Marino M, Caterino M, Ruoppolo M. Proteomic and Bioinformatic Investigation of Altered Pathways in Neuroglobin-Deficient Breast Cancer Cells. Molecules 2021; 26: 2397. doi: 10.3390/molecules26082397.
43. Coumans JV, Gau D, Poljak A, Wasinger V, Roy P, Moens PD. Profilin-1 overexpression in MDA-MB-231 breast cancer cells is associated with alterations in proteomics biomarkers of cell proliferation, survival, and motility as revealed by global proteomics analyses.OMICS 2014; 18: 778-791.doi: 10.1089/omi.2014.0075.

44. Cuomo A, Moretti S, Minucci S, Bonaldi T. SILAC-based proteomic analysis to dissect the "histone modification signature" of human breast cancer cells. Amino Acids 2011; 41: 387-399. doi: 10.1007/s00726-010-0668-2.
45. Del Gaudio F, Guerrera IC, Riccio R, Monti MC. Quantitative proteomics discloses monacolin K-induced alterations in triple-negative breast cancer cell proteomes and phosphoproteomes. Mol Omics 2020; 16: 19-30. doi: 10.1039/c9mo00140a.
46. Devlin L, Okletey J, Perkins G, Bowen JR, Nakos K, Montagna C, Spiliotis ET. Proteomic profiling of the oncogenic septin 9 reveals isoform-specific interactions in breast cancer cells. Proteomics 2021; 21: e2100155. doi: 10.1002/pmic.202100155.
47. Díaz-Chávez J, Fonseca-Sánchez MA, Arechaga-Ocampo E, Flores-Pérez A, Palacios-Rodríguez Y, Domínguez-Gómez G, Marchat LA, Fuentes-Mera L, Mendoza-Hernández G, Gariglio P, López-Camarillo C.Proteomic profiling reveals that resveratrol inhibits HSP27 expression and sensitizes breast cancer cells to doxorubicin therapy. PLoS One 2013; 8: e64378. doi: 10.1371/journal.pone.0064378.
48. DI Cara G, Marengo G, Albanese NN, Marabeti MR, Musso R, Cancemi P, Pucci-Minafra I. Proteomic profiling of Trastuzumab (Herceptin(R))-sensitive and -resistant SKBR-3 breast cancer cells. Anticancer Res 2013; 33: 489-503.
49. Dolai S, Xu Q, Liu F, Molloy MP. Quantitative chemical proteomics in small-scale culture of phorbol ester stimulated basal breast cancer cells. Proteomics 2011; 11: 2683-92.doi: 10.1002/pmic.201000801.
50. D'Santos C, Taylor C, Carroll JS, Mohammed H.RIME proteomics of estrogen and progesterone receptors in breast cancer. Data Brief 2015 Sep 3:5:276-80. doi: 10.1016/j.dib.2015.08.019.
51. Dwek MV, Rawlings SL. Breast cancer proteomics using two-dimensional electrophoresis: studying the breast cancer proteome. Methods Mol Med 2006: 120: 231-43. doi: 10.1385/1-59259-969-9:231.
52. Faktor J, Knopfova L, Lapcik P, Janacova L, Paralova V, Bouchalova P, Muller P, Benes P, Bouchal P. Proteomics Identification and Validation of Desmocollin-1 and Catechol-O-Methyltransferase as Proteins Associated with Breast Cancer Cell Migration and Metastasis. Proteomics 2019; 19: e1900073. doi: 10.1002/pmic.201900073
53. Flodrova D, Benkovska D, Macejova D, Bialesova L, Bobalova J, Brtko J.Effects of retinoic acid isomers on proteomic pattern in human breast cancer MCF-7 cell line. Endocr Regul 2013; 47: 205-209 doi: 10.4149/endo_2013_04_205.
54. Flodrova D, Benkovska D, Macejova D, Bialesova L, Hunakova L, Brtko J, Bobalova J. Proteomic analysis of changes in the protein composition of MCF-7 human breast cancer cells induced by all-trans retinoic acid, 9-cis retinoic acid, and their combination. Toxicol Lett 2015; 232: 226-232. doi: 10.1016/j.toxlet.2014.09.030.
55. Flodrova D, Toporova L, Lastovickova M, Macejova D, Hunakova L, Brtko J, Bobalova J.Consequences of the natural retinoid/retinoid X receptor ligands action in human breast cancer MDA-MB-231 cell line: Focus on functional proteomics. Toxicol Lett 2017 : 281: 26-34. doi: 10.1016/j.toxlet.2017.09.001.
56. Fontana S, Pucci-Minafra I, Becchi M, Freyria AM, Minafra S.Effect of collagen substrates on proteomic modulation of breast cancer cells. Proteomics 2004; 4: 849-860. doi: 10.1002/pmic.200300582.
57. Fujioka H, Sakai A, Tanaka S, Kimura K, Miyamoto A, Iwamoto M, Uchiyama K. Comparative proteomic analysis of paclitaxel resistance-related proteins in human breast cancer cell lines. Oncol Lett 2017; 13: 289-295. doi: 10.3892/ol.2016.5455.
58. Gallegos KM, Patel JR, Llopis SD, Walker RR, Davidson AM, Zhang W, Zhang K, Tilghman SL. Quantitative Proteomic Profiling Identifies a Potential Novel Chaperone Marker in Resistant Breast Cancer. Front Oncol 2021: 11: 540134. doi: 10.3389/fonc.2021.540134.

59. Gangoda L, Liem M, Ang CS, Keerthikumar S, Adda CG, Parker BS, Mathivanan S. Proteomic Profiling of Exosomes Secreted by Breast Cancer Cells with Varying Metastatic Potential. Proteomics 2017; 17. doi: 10.1002/pmic.201600370.
60. Gao Z, Yang YY, Huang M, Qi TF, Wang H, Wang Y. Targeted Proteomic Analysis of Small GTPases in Radioresistant Breast Cancer Cells. Anal Chem 2022; 94: 14925-14930. doi: 10.1021/acs.analchem.2c02389.
61. Ge Y, Rajkumar L, Guzman RC, Nandi S, Patton WF, Agnew BJ. Multiplexed fluorescence detection of phosphorylation, glycosylation, and total protein in the proteomic analysis of breast cancer refractoriness. Proteomics 2004; 4: 3464-3467. doi: 10.1002/pmic.200400957.
62. Gehrmann ML, Hathout Y, Fenselau C. Evaluation of metabolic labeling for comparative proteomics in breast cancer cells. J Proteome Res 2004; 3: 1063-1068. doi: 10.1021/pr049906k.
63. Geiger T, Madden SF, Gallagher WM, Cox J, Mann M. Proteomic portrait of human breast cancer progression identifies novel prognostic markers. Cancer Res 2012; 72: 2428-2439. doi: 10.1158/0008-5472.CAN-11-3711.
64. Gelsomino L, Barone I, Caruso A, Giordano F, Brindisi M, Morello G, Accattatis FM, Panza S, Cappello AR, Bonofiglio D, Andò S, Catalano S, Giordano C. Proteomic Profiling of Extracellular Vesicles Released by Leptin-Treated Breast Cancer Cells: A Potential Role in Cancer Metabolism. Int J Mol Sci 2022; 23: 12941. doi: 10.3390/ijms232112941.
65. Gharbi S, Gaffney P, Yang A, Zvelebil MJ, Cramer R, Waterfield MD, Timms JF. Evaluation of two-dimensional differential gel electrophoresis for proteomic expression analysis of a model breast cancer cell system. Mol Cell Proteomics 2002; 1: 91-98. doi: 10.1074/mcp.t100007-mcp200.
66. Giguère SS, Guise AJ, Jean Beltran PM, Joshi PM, Greco TM, Quach OL, Kong J, Cristea IM. The Proteomic Profile of Deleted in Breast Cancer 1 (DBC1) Interactions Points to a Multifaceted Regulation of Gene Expression. Mol Cell Proteomics 2016; 15: 791-809. doi: 10.1074/mcp.M115.054619.
67. Going CC, Tailor D, Kumar V, Birk AM, Pandrala M, Rice MA, Stoyanova T, Malhotra S, Pitteri SJ. Quantitative Proteomic Profiling Reveals Key Pathways in the Anticancer Action of Methoxychalcone Derivatives in Triple Negative Breast Cancer. J Proteome Res 2018; 17: 3574-3585. doi: 10.1021/acs.jproteome.8b00636.
68. Guney Eskiler G, Yanar S, Akpinar G, Kasap M. Proteomic analysis of talazoparib resistance in triple-negative breast cancer cells. J Biochem Mol Toxicol 2021; 35: e22678. doi: 10.1002/jbt.22678.
69. Guo L, Xiao Y, Fan M, Li JJ, Wang Y.Profiling global kinome signatures of the radioresistant MCF-7/C6 breast cancer cells using MRM-based targeted proteomics, J Proteome Res 2015; 14: 193-201. doi: 10.1021/pr500919w.
70. Guo ML, Sun MX, Lan JZ, Yan LS, Zhang JJ, Hu XX, Xu S, Mao DH, Yang HS, Liu YW, Chen TX. Proteomic analysis of the effects of cell culture density on the metastasis of breast cancer cells. Cell Biochem Funct 2019; 37: 72-83. doi: 10.1002/cbf.3377.
71. Guo Y, Deng X, Wang S, Yuan Y, Guo Z, Hao H, Jiao Y, Li P, Han S.SILAC proteomics based on 3D cell spheroids unveils the role of RAC2 in regulating the crosstalk between triple-negative breast cancer cells and tumor-associated macrophages. Int J Biol Macromol; 254: 127639. doi: 10.1016/j.ijbiomac.2023.127639.
72. Hamler RL, Zhu K, Buchanan NS, Kreunin P, Kachman MT, Miller FR, Lubman DM. A two-dimensional liquid-phase separation method coupled with mass spectrometry for proteomic studies of breast cancer and biomarker identification. Proteomics 2004; 4: 562-577.doi: 10.1002/pmic.200300606.
73. Han R, Sun X, Wu Y, Yang YH, Wang QC, Zhang XT, Ding T, Yang JT.Proteomic and Phosphoproteomic Profiling of Matrix Stiffness-Induced Stemness-Dormancy State Transition in Breast Cancer Cells. J Proteome Res 2024; 23: 4658-4673. doi: 10.1021/acs.jproteome.4c00563.

74. Han R, Wu Y, Yang Y, Wang Q, Ding T, Zhang X, Yang J. Dynamic Proteomic and Acetylomic Profiling of Mechanically Induced Cancer Stemness in Breast Cancer Cells. Proteomics 2025: e202400409. doi: 10.1002/pmic.202400409.
75. Hardouin J, Canelle L, Vlieghe C, Lasserre JP, Caron M, Joubert-Caron R. Proteomic Analysis of the MCF7 Breast Cancer Cell Line. Cancer Genomics Proteomics 2006; 3: 355-368.
76. Hathout Y, Gehrmann ML, Chertov A, Fenselau C. Proteomic phenotyping: metastatic and invasive breast cancer. Cancer Lett 2004; 210: 245-253. doi: 10.1016/j.canlet.2004.01.019.
77. Hoedt E, Chaoui K, Huvent I, Mariller C, Monsarrat B, Burlet-Schiltz O, Pierce A. SILAC-based proteomic profiling of the human MDA-MB-231 metastatic breast cancer cell line in response to the two antitumoral lactoferrin isoforms: the secreted lactoferrin and the intracellular delta-lactoferrin. PLoS One 2014; 9: e104563. doi: 10.1371/journal.pone.0104563.
78. Hong KU, Gardner JQ, Doll MA, Stepp MW, Wilkey DW, Benz FW, Cai J, Merchant ML, Hein DW. Dataset for proteomic analysis of arylamine *N*-acetyltransferase 1 knockout MDA-MB-231 breast cancer cells. Data Brief 2022: 45:108634. doi: 10.1016/j.dib.2022.108634.
79. Hong KU, Gardner JQ, Doll MA, Stepp MW, Wilkey DW, Benz FW, Cai J, Merchant ML, Hein DW. Proteomic analysis of arylamine *N*-acetyltransferase 1 knockout breast cancer cells: Implications in immune evasion and mitochondrial biogenesis. Toxicol Rep 2022: 9: 1566-1573. doi: 10.1016/j.toxrep.2022.07.010.
80. Hu ZZ, Kagan BL, Ariazi EA, Rosenthal DS, Zhang L, Li JV, Huang H, Wu C, Jordan VC, Riegel AT, Wellstein A. Proteomic analysis of pathways involved in estrogen-induced growth and apoptosis of breast cancer cells. PLoS One 2011; 6: e20410.doi: 10.1371/journal.pone.0020410.
81. Huang A, Huang SY, Shah P, Ku WC, Huang KT, Liu YF, Su CL, Huang RFS. Suboptimal folic acid exposure rewires oncogenic metabolism and proteomics signatures to mediate human breast cancer malignancy. Nutr Biochem 2022: 106: 109000. doi: 10.1016/j.jnutbio.2022.109000.
82. Huang HJ, Lin CC, Chou HC, Chen YW, Lin ST, Lin YC, Lin DY, Lyu KW, Chan HL. Proteomic analysis of rhein-induced cyt: ER stress mediates cell death in breast cancer cells. Mol Biosyst 2014; 10: 3086-3100. doi: 10.1039/c4mb00451e.
83. Huber M, Bahr I, Krätzschmar JR, Becker A, Müller EC, Donner P, Pohlenz HD, Schneider MR, Sommer A. Comparison of proteomic and genomic analyses of the human breast cancer cell line T47D and the antiestrogen-resistant derivative T47D-r. Mol Cell Proteomics 2004; 3: 43-55. doi: 10.1074/mcp.M300047-MCP200.
84. Ilieș M, Uifălean A, Pașca S, Dhople VM, Lalk M, Iuga CA, Hammer E. From Proteomics to Personalized Medicine: The Importance of Isoflavone Dose and Estrogen Receptor Status in Breast Cancer Cells. J Pers Med 2020; 10: 292. doi: 10.3390/jpm10040292.
85. Imai K, Ichibangase T, Saitoh R, Hoshikawa Y. A proteomics study on human breast cancer cell lines by fluorogenic derivatization-liquid chromatography/tandem mass spectrometry. Biomed Chromatogr 2008; 22: 1304-1314. doi: 10.1002/bmc.1102.
86. Jami MS, Hou J, Liu M, Varney ML, Hassan H, Dong J, Geng L, Wang J, Yu F, Huang X, Peng H, Fu K, Li Y, Singh RK, Ding SJ. Functional proteomic analysis reveals the involvement of KIAA1199 in breast cancer growth, motility and invasiveness. BMC Cancer 2014: 14:194. doi: 10.1186/1471-2407-14-194.
87. Jin G, Wang K, Liu Y, Liu X, Zhang X, Zhang H. Proteomic Level Changes on Treatment in MCF-7/DDP Breast Cancer Drug- Resistant Cells. Anticancer Agents Med Chem 2020; 20: 687-699. doi: 10.2174/1871520620666200213102849.
88. Journet A, Chapel A, Kieffer S, Roux F, Garin J. Proteomic analysis of human lysosomes: application to monocytic and breast cancer cells. Proteomics 2002; 2: 1026-1040. doi: 10.1002/1615-9861(200208)2:8<1026::AID-PROT1026>3.0.CO;2-I.

89. Kalita-de Croft P, Straube J, Lim M, Al-Ejeh F, Lakhani SR, Saunus JM. Proteomic Analysis of the Breast Cancer Brain Metastasis Microenvironment. Int J Mol Sci 2019; 20: 2524. doi: 10.3390/ijms20102524.
90. Kalocsay M, Berberich MJ, Everley RA, Nariya MK, Chung M, Gaudio B, Victor C, Bradshaw GA, Eisert RJ, Hafner M, Sorger PK, Mills CE, Subramanian K. Proteomic profiling across breast cancer cell lines and models. Sci Data 2023; 10: 514. doi: 10.1038/s41597-023-02355-0.
91. Kamal AH, Han BS, Choi JS, Cho K, Kim SY, Kim WK, Lee SC, Bae KH.Proteomic analysis of the effect of retinoic acids on the human breast cancer cell line MCF-7. Mol Biol Rep 2014 May;41(5):3499-507. doi: 10.1007/s11033-014-3212-8.
92. Kanaujiya JK, Lochab S, Kapoor I, Pal P, Datta D, Bhatt ML, Sanyal S, Behre G, Trivedi AK. Proteomic identification of Profilin1 as a corepressor of estrogen receptor alpha in MCF7 breast cancer cells. Proteomics 2013; 13: 2100-2112. doi: 10.1002/pmic.201200534.
93. Kanugula AK, Dhople VM, Völker U, Ummanni R, Kotamraju S. Fluvastatin mediated breast cancer cell death: a proteomic approach to identify differentially regulated proteins in MDA-MB-231 cells. PLoS One 2014; 9: e108890. doi: 10.1371/journal.pone.0108890.
94. Karcini A, Mercier NR, Lazar IM.Proteomic assessment of SKBR3/HER2+ breast cancer cellular response to Lapatinib and investigational Ipatasertib kinase inhibitors. Front Pharmacol 2024: 15: 1413818. doi: 10.3389/fphar.2024.1413818.
95. Khazaei G, Shamsabadi FT, Yamchi A, Golalipour M, Jhingan GD, Shahbazi M. Proteomics evaluation of MDA-MB-231 breast cancer cells in response to RNAi-induced silencing of hPTTG. Life Sci 2019: 239: 116873. doi: 10.1016/j.lfs.2019.116873.
96. Kim H, Woo J, Dan K, Lee KM, Jin MS, Park IA, Ryu HS, Han D. Quantitative Proteomics Reveals Knockdown of CD44 Promotes Proliferation and Migration in Claudin-Low MDA-MB-231 and Hs 578T Breast Cancer Cell Lines. J Proteome Res 2021; 20: 3720-3733. doi: 10.1021/acs.jproteome.1c00293.
97. Kim MH, Jung SY, Ahn J, Hwang SG, Woo HJ, An S, Nam SY, Lim DS, Song JY. Quantitative proteomic analysis of single or fractionated radiation-induced proteins in human breast cancer MDA-MB-231 cells. Cell Biosci 2015: 5: 2. doi: 10.1186/2045-3701-5-2.
98. Kischel P, Guillonneau F, Dumont B, Bellahcène A, Stresing V, Clézardin P, De Pauw EA, Castronovo V. Cell membrane proteomic analysis identifies proteins differentially expressed in osteotropic human breast cancer cells. Neoplasia 2008; 10: 1014-1020.doi: 10.1593/neo.08570.
99. Klawitter J, Klawitter J, Gurshtein J, Corby K, Fong S, Tagliaferri M, Quattrochi L, Cohen I, Shtivelman E, Christians U. Bezielle (BZL101)-induced oxidative stress damage followed by redistribution of metabolic fluxes in breast cancer cells: a combined proteomic and metabolomic study. Int J Cancer 2011; 129: 2945-2957.doi: 10.1002/ijc.25965.
100. Koh EY, You JE, Jung SH, Kim PH. Biological Functions and Identification of Novel Biomarker Expressed on the Surface of Breast Cancer-Derived Cancer Stem Cells via Proteomic Analysis. Mol Cells 2020; 43: 384-396. doi: 10.14348/molcells.2020.2230.
101. Korak T, Bal Albayrak MG, Kasap M, Akpinar G. Thymoquinone and Metabolic Reprogramming in Breast Cancer: A New Dimension From Proteomic Analysis. J Biochem Mol Toxicol 2025; 39: e70124. doi: 10.1002/jbt.70124.
102. Kosok M, Alli-Shaik A, Bay BH, Gunaratne J. Comprehensive Proteomic Characterization Reveals Subclass-Specific Molecular Aberrations within Triple-negative Breast Cancer. iScience 2020; 23: 100868. doi: 10.1016/j.isci.2020.100868.
103. Kuciauskas D, Dreize N, Ger M, Kaupinis A, Zemaitis K, Stankevicius V, Suziedelis K, Cicenas J, Graves LM, Valius M. Proteomic Analysis of Breast Cancer Resistance to the Anticancer Drug RH1 Reveals the Importance of Cancer Stem Cells. Cancers (Basel) 2019; 11: 972. doi: 10.3390/cancers11070972.
104. Kulasingam V, Diamandis EP.Proteomics analysis of conditioned media from three breast cancer cell lines: a mine for biomarkers and therapeutic targets. Mol Cell Proteomics 2007; 6: 1997-2011. doi: 10.1074/mcp.M600465-MCP200.

105. Kulkarni YM, Suarez V, Klinke DJ 2nd. Inferring predominant pathways in cellular models of breast cancer using limited sample proteomic profiling. BMC Cancer 2010: 10: 291.doi: 10.1186/1471-2407-10-291.
106. Kusza DA, Hunter R, Schäfer G, Smith M, Katz AA, Kaschula CH. Activity-Based Proteomic Identification of the *S*-Thiolation Targets of Ajoene in MDA-MB-231 Breast Cancer Cells. J Agric Food Chem 2022; 70: 14679-14692. doi: 10.1021/acs.jafc.2c04972.
107. Lacasse V, Beaudoin S, Jean S, Leyton JV. A Novel Proteomic Method Reveals NLS Tagging of T-DM1 Contravenes Classical Nuclear Transport in a Model of HER2-Positive Breast Cancer. Mol Ther Methods Clin Dev 2020: 19:99-119. doi: 10.1016/j.omtm.2020.08.016.
108. Lai TC, Chou HC, Chen YW, Lee TR, Chan HT, Shen HH, Lee WT, Lin ST, Lu YC, Wu CL, Chan HL. Secretomic and proteomic analysis of potential breast cancer markers by two-dimensional differential gel electrophoresis. J Proteome Res 2010; 9: 1302-1322.doi: 10.1021/pr900825t.
109. Lazzarini R, Eléxpuru-Zabaleta M, Piva F, Giulietti M, Fulgenzi G, Tartaglione MF, Zingaretti L, Tagliabracci A, Valentino M, Santarelli L, Bracci M. Effects of extremely low-frequency magnetic fields on human MDA-MB-231 breast cancer cells: proteomic characterization. Ecotoxicol Environ Saf 2023: 253: 114650. doi: 10.1016/j.ecoenv.2023.114650.
110. Lee K, Wang T, Paszczynski AJ, Daoud SS. Expression proteomics to p53 mutation reactivation with PRIMA-1 in breast cancer cells. BiochemBiophys Res Commun 2006; 349: 1117-1124. doi: 10.1016/j.bbrc.2006.08.152.
111. Lei D, Hong T, Li L, Chen L, Luo X, Wu Q, Liu Z. Isobaric tags for relative and absolute quantitation-based proteomics analysis of the effect of ginger oil on bisphenol A-induced breast cancer cell proliferation. Oncol Lett 2021; 21: 101. doi: 10.3892/ol.2020.12362.
112. Leong S, Nunez AC, Lin MZ, Crossett B, Christopherson RI, Baxter RC. iTRAQ-based proteomic profiling of breast cancer cell response to doxorubicin and TRAIL. J Proteome Res2012; 11: 3561-3572.doi: 10.1021/pr2012335.
113. Li G, Zhao F, Cui Y.Proteomics using mammospheres as a model system to identify proteins deregulated in breast cancer stem cells. Curr Mol Med 2013; 13: 459-463.
114. Li H, Meng F, Jiang L, Ren Y, Qiu Z, Yu P, Peng J. Comparison of LC-MS/MS-based targeted proteomics and conventional analytical methods for monitoring breast cancer resistance protein expression. Comparative Study Life Sci 2019: 231: 116548. doi: 10.1016/j.lfs.2019.116548.
115. Li X, Sun H, Hou Y, Jin W. Comprehensive Combined Proteomics and Genomics Analysis Identifies Prognostic Related Transcription Factors in Breast Cancer and Explores the Role of DMAP1 in Breast Cancer. J Pers Med 2021; 11: 1068. doi: 10.3390/jpm11111068.
116. Liao Z, Thomas SN, Wan Y, Lin HH, Ann DK, Yang AJ. An Internal Standard-Assisted Synthesis and Degradation Proteomic Approach Reveals the Potential Linkage between VPS4B Depletion and Activation of Fatty Acid β-Oxidation in Breast Cancer Cells. Int J Proteomics 2013: 2013: 291415. doi: 10.1155/2013/291415.
117. Ließem A, Leimer U, Germann GK, Köllensperger E.Adipokines in Breast Cancer: Decoding Genetic and Proteomic Mechanisms Underlying Migration, Invasion, and Proliferation. Breast Cancer (Dove Med Press) 2025; 17: 79-102. doi: 10.2147/BCTT.S491277.
118. Liu C, Xu M, Li W, Cao X, Wang Y, Chen H, Zhang T, Lu M, Xie H, Chen Y.Quantitative Pattern of hPTMs by Mass Spectrometry-Based Proteomics with Implications for Triple-Negative Breast Cancer. J Proteome Res 2024; 23: 1495-1505. doi: 10.1021/acs.jproteome.4c00034.
119. Liu Y, Liu H, Han B, Zhang JT.Identification of 14-3-3sigma as a contributor to drug resistance in human breast cancer cells using functional proteomic analysis.Cancer Res 2006; 66: 3248-3255. doi: 10.1158/0008-5472.CAN-05-3801.

120. Madden SF, Cremona M, Farrelly AM, Low WH, McBryan J. Proteomic time course of breast cancer cells highlights enhanced sensitivity to Stat3 and Src inhibitors prior to endocrine resistance development. Cancer Gene Ther 2023; 30: 324-334. doi: 10.1038/s41417-022-00548-0.
121. Madoz-Gúrpide J, Serrano-López J, Sanz-Álvarez M, Morales-Gallego M, Rodríguez-Pinilla SM, Rovira A, Albanell J, Rojo F. Adaptive Proteomic Changes in Protein Metabolism and Mitochondrial Alterations Associated with Resistance to Trastuzumab and Pertuzumab Therapy in HER2-Positive Breast Cancer.Int J Mol Sci 2025; 26: 1559. doi: 10.3390/ijms26041559.
122. Malorni L, Cacace G, Cuccurullo M, Pocsfalvi G, Chambery A, Farina A, Di Maro A, Parente A, Malorni A.Proteomic analysis of MCF-7 breast cancer cell line exposed to mitogenic concentration of 17beta-estradiol. Proteomics 2006; 6: 5973-5982. doi: 10.1002/pmic.200600333.
123. Maurizio E, Wiśniewski JR, Ciani Y, Amato A, Arnoldo L, Penzo C, Pegoraro S, Giancotti V, Zambelli A, Piazza S, Manfioletti G, Sgarra R.Translating Proteomic Into Functional Data: An High Mobility Group A1 (HMGA1) Proteomic Signature Has Prognostic Value in Breast Cancer. Mol Cell Proteomics 2016; 15: 109-123. doi: 10.1074/mcp.M115.050401.
124. Melwani PK, Balla MMS, S N, Padwal M, Chaurasia RK, Basu B, Ghosh A, Pandey BN. Integrated transcriptomic and proteomic analysis of microplasts derived from macrophage-conditioned medium-treated MCF-7 breast cancer cells. FEBS Lett 2021; 595: 1844-1860. doi: 10.1002/1873-3468.14108.
125. Miao W, Bade D, Wang Y. Targeted Proteomic Analysis Revealed Kinome Reprogramming during Acquisition of Radioresistance in Breast Cancer Cells. J Proteome Res 2021; 20: 2830-2838. doi: 10.1021/acs.jproteome.1c00075.
126. Mishra M, Sharma A, Thacker G, Trivedi AK. Nano-LC based proteomic approach identifies that E6AP interacts with ENO1 and targets it for degradation in breast cancer cells.IUBMB Life 2019; 71: 1896-1905. doi: 10.1002/iub.2132.
127. Mittal L, Aryal UK, Camarillo IG, Ferreira RM, Sundararajan R. Quantitative proteomic analysis of enhanced cellular effects of electrochemotherapy with Cisplatin in triple-negative breast cancer cells. Sci Rep 2019; 9: 13916. doi: 10.1038/s41598-019-50048-9.
128. Mittal L, Aryal UK, Camarillo IG, Raman V, Sundararajan R. Effective electrochemotherapy with curcumin in MDA-MB-231-human, triple negative breast cancer cells: A global proteomics study. Bioelectrochemistry 2020: 131: 107350. doi: 10.1016/j.bioelechem.2019.107350.
129. Monge M, Vilaseca M, Soto-Cerrato V, Montaner B, Giralt E, Pérez-Tomás R. Proteomic analysis of prodigiosin-induced apoptosis in a breast cancer mitoxantrone-resistant (MCF-7 MR) cell line. Invest New Drugs 2007; 25: 21-29.doi: 10.1007/s10637-006-7774-8.
130. Moradpoor R, Gharebaghian A, Shahi F, Mousavi A, Salari S, Akbari ME, Ajdari S, Salimi M.Identification and Validation of Stage-Associated PBMC Biomarkers in Breast Cancer Using MS-Based Proteomics. Front Oncol 2020: 10: 1101. doi: 10.3389/fonc.2020.01101.
131. Morrison C, Mancini S, Cipollone J, Kappelhoff R, Roskelley C, Overall C. Microarray and proteomic analysis of breast cancer cell and osteoblast co-cultures: role of osteoblast matrix metalloproteinase (MMP)-13 in bone metastasis. J Biol Chem 2011; 286: 34271-34285.doi: 10.1074/jbc.M111.222513.
132. Nagaprashantha LD, Singhal J, Chikara S, Gugiu G, Horne D, Awasthi S, Salgia R, Singhal SS. 2'-Hydroxyflavanone induced changes in the proteomic profile of breast cancer cells. J Proteomics 2019: 192: 233-245. doi: 10.1016/j.jprot.2018.09.005.
133. Nagaraja GM, Othman M, Fox BP, Alsaber R, Pellegrino CM, Zeng Y, Khanna R, Tamburini P, Swaroop A, Kandpal RP. Gene expression signatures and biomarkers of noninvasive and invasive breast cancer cells: comprehensive profiles by representational difference analysis, microarrays and proteomics. Oncogene 2006; 25: 2328-2338.doi: 10.1038/sj.onc.1209265.
134. Netsirisawan P, Chokchaichamnankit D, Saharat K, Srisomsap C, Svasti J, Champattanachai V. Quantitative proteomic analysis of the association between decreasing O-GlcNAcylation and

metastasis in MCF-7 breast cancer cells. Int J Oncol 2020; 56: 1387-1404. doi: 10.3892/ijo.2020.5022.
135. Netsirisawan P, Chokchaichamnankit D, Srisomsap C, Svasti J, Champattanachai V. Proteomic Analysis Reveals Aberrant O-GlcNAcylation of Extracellular Proteins from Breast Cancer Cell Secretion. Cancer Genomics Proteomics 2015; 12: 201-209.
136. Ngoka LC. Sample prep for proteomics of breast cancer: proteomics and gene ontology reveal dramatic differences in protein solubilization preferences of radioimmunoprecipitation assay and urea lysis buffers. Proteome Sci 2008: 6: 30.doi: 10.1186/1477-5956-6-30.
137. Nie S, McDermott SP, Deol Y, Tan Z, Wicha MS, Lubman DM.A quantitative proteomics analysis of MCF7 breast cancer stem and progenitor cell populations. Proteomics 2015; 15: 3772-3783. doi: 10.1002/pmic.201500002.
138. Ning B, Liu C, Kucukdagli AC, Zhang J, Jing H, Zhou Z, Zhang Y, Dong Y, Chen Y, Guo H, Xu J. Proteomic profiling identifies upregulation of aurora kinases causing resistance to taxane-type chemotherapy in triple negative breast cancer. Sci Rep 2025; 15: 3211. doi: 10.1038/s41598-025-87315-x.
139. Niu M, Klingler-Hoffmann M, Brazzatti JA, Forbes B, Akekawatchai C, Hoffmann P, McColl SR. Comparative proteomic analysis implicates eEF2 as a novel target of PI3Kγ in the MDA-MB-231 metastatic breast cancer cell line. Proteome Sci 2013; 11: 4. doi: 10.1186/1477-5956-11-4.
140. Norouzi S, Yazdian Robati R, Ghandadi M, Abnous K, Behravan J, Mosaffa F.Comparative proteomics study of proteins involved in induction of higher rates of cell death in mitoxantrone-resistant breast cancer cells MCF-7/MX exposed to TNF-α. Iran J Basic Med Sci 2020; 23: 663-672. doi: 10.22038/ijbms.2020.40029.9486.
141. Obuchi W, Ohtsuki S, Uchida Y, Ohmine K, Yamori T, Terasaki T. Identification of transporters associated with Etoposide sensitivity of stomach cancer cell lines and methotrexate sensitivity of breast cancer cell lines by quantitative targeted absolute proteomics. Mol Pharmacol 2013; 83: 490-500. doi: 10.1124/mol.112.081083.
142. Okur E, Yerlikaya A. A novel and effective inhibitor combination involving bortezomib and OTSSP167 for breast cancer cells in light of label-free proteomic analysis. Cell Biol Toxicol 2019; 35: 33-47. doi: 10.1007/s10565-018-9435-z.
143. Oliveras-Ferraros C, Vazquez-Martin A, Martin-Castilló B, Pérez-Martínez MC, Cufí S, Del Barco S, Bernado L, Brunet J, López-Bonet E, Menendez JA. Pathway-focused proteomic signatures in HER2-overexpressing breast cancer with a basal-like phenotype: new insights into de novo resistance to trastuzumab (Herceptin). Int J Oncol 2010; 37: 669-678. doi: 10.3892/ijo_00000716.
144. Ouchida M, Kanzaki H, Ito S, Hanafusa H, Jitsumori Y, Tamaru S, Shimizu K. Novel direct targets of miR-19a identified in breast cancer cells by a quantitative proteomic approach. PLoS One 2012; 7: e44095.doi: 10.1371/journal.pone.0044095.
145. Palazzolo G, Albanese NN, DI Cara G, Gygax D, Vittorelli ML, Pucci-Minafra I.Proteomic analysis of exosome-like vesicles derived from breast cancer cells. Anticancer Res 2012; 32: 847-60.
146. Palma Cde S, Grassi ML, Thomé CH, Ferreira GA, Albuquerque D, Pinto MT, Ferreira Melo FU, Kashima S, Covas DT, Pitteri SJ, Faça VM. Proteomic Analysis of Epithelial to Mesenchymal Transition (EMT) Reveals Cross-talk between SNAIL and HDAC1 Proteins in Breast Cancer Cells. Mol Cell Proteomics 2016; 15: 906-917. doi: 10.1074/mcp.M115.052910.
147. Panneer Selvam A, Wong J, Flanagan K, Prasad S. Cellular level classification of breast cancer through proteomic markers using nanochannel array sensors. Nanomedicine (Lond) 2014; 9: 1957-1970. doi: 10.2217/nnm.13.152.
148. Park SS, Kim DS, Park KS, Song HJ, Kim SY. Proteomic analysis of high-molecular-weight protein polymers in a doxorubicin-resistant breast-cancer cell line. Proteomics Clin Appl 2007; 1: 555-560. doi: 10.1002/prca.200700122.

149. Pateetin P, Pisitkun T, McGowan E, Boonyaratanakornkit V. Differential quantitative proteomics reveals key proteins related to phenotypic changes of breast cancer cells expressing progesterone receptor A. J Steroid Biochem Mol Biol 2020: 198: 105560. doi: 10.1016/j.jsbmb.2019.105560.
150. Pavlou MP, Dimitromanolakis A, Diamandis EP. Coupling proteomics and transcriptomics in the quest of subtype-specific proteins in breast cancer. Proteomics 2013; 13: 1083-1095. doi: 10.1002/pmic.201200526.
151. Peng W, Zhang Y, Zhu R, Mechref Y.Comparative membrane proteomics analyses of breast cancer cell lines to understand the molecular mechanism of breast cancer brain metastasis. Comparative Study Electrophoresis 2017; 38: 2124-2134. doi: 10.1002/elps.201700027.
152. Peng W, Zhu R, Zhou S, Mirzaei P, Mechref Y. Integrated Transcriptomics, Proteomics, and Glycomics Reveals the Association between Up-regulation of Sialylated N-glycans/Integrin and Breast Cancer Brain Metastasis. Sci Rep 2019; 9: 17361. doi: 10.1038/s41598-019-53984-8.
153. Perera CN, Spalding HS, Mohammed SI, Camarillo IG. Identification of proteins secreted from leptin stimulated MCF-7 breast cancer cells: a dual proteomic approach. Exp Biol Med (Maywood) 2008; 233: 708-720.doi: 10.3181/0710-RM-281.
154. Perri AM, Agosti V, Olivo E, Concolino A, Angelis M, Tammè L, Fiumara CV, Cuda G, Scumaci D. Histone proteomics reveals novel post-translational modifications in breast cancer. Aging (Albany NY) 2019; 11: 11722-11755. doi: 10.18632/aging.102577.
155. Pionneau C, Canelle L, Bousquet J, Hardouin J, Bigeard J, Caron M, Joubert-Caron R. Proteomic Analysis of Membrane-associated Proteins from the Breast Cancer Cell Line MCF7. Cancer Genomics Proteomics 2005; 2: 199-207.
156. Pralea IE, Moldovan RC, Țigu AB, Moldovan CS, Fischer-Fodor E, Iuga CA. Cellular Responses Induced by NCT-503 Treatment on Triple-Negative Breast Cancer Cell Lines: A Proteomics Approach. Biomedicines 2024; 12: 1087. doi: 10.3390/biomedicines12051087.
157. Pucci-Minafra I, Cancemi P, Di Cara G, Minafra L, Feo S, Forlino A, Tira ME, Tenni R, Martini D, Ruggeri A, Minafra S.Decorin transfection induces proteomic and phenotypic modulation in breast cancer cells 8701-BC. Connect Tissue Res 2008; 49: 30-41.doi: 10.1080/03008200701820443.
158. Pucci-Minafra I, Fontana S, Cancemi P, Alaimo G, Minafra S. Proteomic patterns of cultured breast cancer cells and epithelial mammary cells. Ann N Y Acad Sci 2002: 963: 122-139. doi: 10.1111/j.1749-6632.2002.tb04103.x.
159. Pucci-Minafra I, Fontana S, Cancemi P, Basiricò L, Caricato S, Minafra S. A contribution to breast cancer cell proteomics: detection of new sequences. Proteomics 2002; 2: 919-927. doi: 10.1002/1615-9861(200207)2:7<919::AID-PROT919>3.0.CO;2-P.
160. Qattan AT, Mulvey C, Crawford M, Natale DA, Godovac-Zimmermann J.Quantitative organelle proteomics of MCF-7 breast cancer cells reveals multiple subcellular locations for proteins in cellular functional processes. J Proteome Res 2010; 9: 495-508.doi: 10.1021/pr9008332.
161. Raman V, Aryal UK, Hedrick V, Ferreira RM, Fuentes Lorenzo JL, Stashenko EE, Levy M, Levy MM, Camarillo IG.Proteomic Analysis Reveals That an Extract of the Plant Lippia origanoides Suppresses Mitochondrial Metabolism in Triple-Negative Breast Cancer Cells. J Proteome Res 2018; 17: 3370-3383. doi: 10.1021/acs.jproteome.8b00255.
162. Ramdas P, Radhakrishnan AK, Abdu Sani AA, Abdul-Rahman PS. Tocotrienols Modulate Breast Cancer Secretomes and Affect Cancer-Signaling Pathways in MDA-MB-231 Cells: A Label-Free Quantitative Proteomic Analysis. Nutr Cancer 2019; 71: 1263-1271. doi: 10.1080/01635581.2019.1607407.
163. Ramdas P, Radhakrishnan AK, Abdu Sani AA, Kumari M, Anandha Rao JS, Abdul-Rahman PS. Advancing the Role of Gamma-Tocotrienol as Proteasomes Inhibitor: A Quantitative Proteomic Analysis of MDA-MB-231 Human Breast Cancer Cells. Biomolecules 2019; 10: 19. doi: 10.3390/biom10010019.

164. Rees JS, Cheung LCC, Hamaia SW, Davies G, Sandercock A, Lilley KS, Tigue N, Jackson AP. Identification of the cis-molecular neighbours of the immune checkpoint protein B7-H4 in the breast cancer cell-line SK-BR-3 by proteomic proximity labelling. Int J Oncol 2020; 57: 87-99. doi: 10.3892/ijo.2020.5037.
165. Rehman A, Chahal MS, Tang X, Bruce JE, Pommier Y, Daoud SS. Proteomic identification of heat shock protein 90 as a candidate target for p53 mutation reactivation by PRIMA-1 in breast cancer cells. Breast Cancer Res 2005; 7: R765-74. doi: 10.1186/bcr1290.
166. Roehrer S, Stork V, Ludwig C, Minceva M, Behr J. Analyzing bioactive effects of the minor hop compound xanthohumol C on human breast cancer cells using quantitative proteomics. PLoS One 2019; 14: e0213469. doi: 10.1371/journal.pone.0213469.
167. Rontogianni S, Synadaki E, Li B, Liefaard MC, Lips EH, Wesseling J, Wu W, Altelaar M. Proteomic profiling of extracellular vesicles allows for human breast cancer subtyping. Commun Biol 2019: 2: 325. doi: 10.1038/s42003-019-0570-8.
168. Rosso M, Lapyckyj L, Besso MJ, Monge M, Reventós J, Canals F, Quevedo Cuenca JO, Matos ML, Vazquez-Levin MH. Characterization of the molecular changes associated with the overexpression of a novel epithelial cadherin splice variant mRNA in a breast cancer model using proteomics and bioinformatics approaches: identification of changes in cell metabolism and an increased expression of lactate dehydrogenase B. Cancer Metab 2019: 7: 5. doi: 10.1186/s40170-019-0196-9.
169. Rowell C, Carpenter DM, Lamartiniere CA. Chemoprevention of breast cancer, proteomic discovery of genistein action in the rat mammary gland. J Nutr 2005; 135: 2953S-2959S.doi: 10.1093/jn/135.12.2953S.
170. Rust S, Guillard S, Sachsenmeier K, Hay C, Davidson M, Karlsson A, Karlsson R, Brand E, Lowne D, Elvin J, Flynn M, Kurosawa G, Hollingsworth R, Jermutus L, Minter R. Combining phenotypic and proteomic approaches to identify membrane targets in a 'triple negative' breast cancer cell type. Mol Cancer 2013: 12: 11. doi: 10.1186/1476-4598-12-11.
171. Sacco F, Silvestri A, Posca D, Pirrò S, Gherardini PF, Castagnoli L, Mann M, Cesareni G.Deep Proteomics of Breast Cancer Cells Reveals that Metformin Rewires Signaling Networks Away from a Pro-growth State. Cell Syst 2016; 2: 159-171. doi: 10.1016/j.cels.2016.02.005.
172. Sandhu C, Hewel JA, Badis G, Talukder S, Liu J, Hughes TR, Emili A. Evaluation of data-dependent versus targeted shotgun proteomic approaches for monitoring transcription factor expression in breast cancer. J Proteome Res 2008; 7: 1529-1541.doi: 10.1021/pr700836q.
173. Segovia-Mendoza M, Gómez de León CT, García-Becerra R, Ambrosio J, Nava-Castro KE, Morales-Montor J. The chemical environmental pollutants BPA and BPS induce alterations of the proteomic profile of different phenotypes of human breast cancer cells: A proposed interactome. Environ Res 2020: 191:109960. doi: 10.1016/j.envres.2020.109960.
174. Sengodan SK, Rajan A, Hemalatha SK, Nadhan R, Jaleel A, Srinivas P. Proteomic Profiling of β-hCG-Induced Spheres in BRCA1 Defective Triple Negative Breast Cancer Cells. J Proteome Res 2018; 17: 276-289. doi: 10.1021/acs.jproteome.7b00562.
175. Sharaf BM, Giddey AD, Al-Hroub HM, Menon V, Okendo J, El-Awady R, Mousa M, Almehdi A, Semreen MH, Soares NC. Mass spectroscopy-based proteomics and metabolomics analysis of triple-positive breast cancer cells treated with tamoxifen and/or trastuzumab. Cancer Chemother Pharmacol 2022; 90: 467-488. doi: 10.1007/s00280-022-04478-4.
176. Shaw PG, Chaerkady R, Wang T, Vasilatos S, Huang Y, Van Houten B, Pandey A, Davidson NE. Integrated proteomic and metabolic analysis of breast cancer progression. PLoS One 2013; 8: e76220. doi: 10.1371/journal.pone.0076220.
177. Shen S, Tu C, Shen H, Li J, Frangou C, Zhang J, Qu J. Comparative Proteomics Analysis of Exosomes Identifies Key Pathways and Protein Markers Related to Breast Cancer Metastasis. Int J Mol Sci 2023; 24: 4033. doi: 10.3390/ijms24044033.

178. Shin J, Kim G, Lee JW, Lee JE, Kim YS, Yu JH, Lee ST, Ahn SH, Kim H, Lee C. Identification of ganglioside GM2 activator playing a role in cancer cell migration through proteomic analysis of breast cancer secretomes. Cancer Sci 2016; 107: 828-835. doi: 10.1111/cas.12935.
179. Sinai-Livne T, Pasmanik-Chor M, Cohen Z, Tsarfaty I, Werner H, Berger R. Proteomic analysis of combined IGF1 receptor targeted therapy and chemotherapy identifies signatures associated with survival in breast cancer patients. Oncotarget 2020; 11: 1515-1530. doi: 10.18632/oncotarget.27566.
180. Singh R, Avliyakulov NK, Braga M, Haykinson MJ, Martinez L, Singh V, Parveen M, Chaudhuri G, Pervin S. Proteomic identification of mitochondrial targets of arginase in human breast cancer. PLoS One 2013; 8: e79242. doi: 10.1371/journal.pone.0079242.
181. Smith L, Welham KJ, Watson MB, Drew PJ, Lind MJ, Cawkwell L. The proteomic analysis of cisplatin resistance in breast cancer cells. Oncol Res 2007; 16: 497-506.doi: 10.3727/096504007783438358.
182. Somasekhara D, Dammalli M, Nadumane VK. Proteomic Analysis of Human Breast Cancer MCF-7 Cells to Identify Cellular Targets of the Anticancer Pigment OR3 from Streptomyces coelicolor JUACT03. Appl Biochem Biotechnol 2023; 195: 236-252. doi: 10.1007/s12010-022-04128-8.
183. Sommer AK, Hermawan A, Ljepoja B, Fröhlich T, Arnold GJ, Wagner E, Roidl A. A proteomic analysis of chemoresistance development via sequential treatment with doxorubicin reveals novel players in MCF-7 breast cancer cells. Int J Mol Med 2018; 42: 1987-1997. doi: 10.3892/ijmm.2018.3781.
184. Sotoca AM, Gelpke MD, Boeren S, Ström A, Gustafsson JÅ, Murk AJ, Rietjens IM, Vervoort J. Quantitative proteomics and transcriptomics addressing the estrogen receptor subtype-mediated effects in T47D breast cancer cells exposed to the phytoestrogen genistein. Mol Cell Proteomics 2011; 10: M110.002170. doi: 10.1074/mcp.M110.002170.
185. Spínola-Lasso E, Montero JC, Jiménez-Monzón R, Estévez F, Quintana J, Guerra B, Elokely KM, León F, Del Rosario H, Fernández-Pérez L, López MR, Díaz-Chico BN, McNaughton-Smith G, Pandiella A, Díaz-Chico JC. Chemical-proteomics Identify Peroxiredoxin-1 as an Actionable Target in Triple-negative Breast Cancer. Int J Biol Sci 2023; 19: 1731-1747. doi: 10.7150/ijbs.78554.
186. Stebbing J, Zhang H, Xu Y, Grothey A, Ajuh P, Angelopoulos N, Giamas G.Characterization of the Tyrosine Kinase-Regulated Proteome in Breast Cancer by Combined use of RNA interference (RNAi) and Stable Isotope Labeling with Amino Acids in Cell Culture (SILAC) Quantitative Proteomics. Mol Cell Proteomics 2015; 14: 2479-2492. doi: 10.1074/mcp.M115.048090.
187. Strouhalova D, Macejova D, Lastovickova M, Brtko J, Bobalova J. CD44 and vimentin, markers involved with epithelial-mesenchymal transition: A proteomic analysis of sequential proteins extraction of triple-negative breast cancer cells after treatment with all-trans retinoic acid. Gen Physiol Biophys 2020; 39: 399-405. doi: 10.4149/gpb_2020026.
188. Strouhalova D, Toporova L, Lastovickova M, Macejova D, Bobalova J, Brtko J. Novel insights into the combined effect of triorganotin compounds and all-trans retinoic acid on expression of selected proteins associated with tumor progression in breast cancer cell line MDA-MB-231: Proteomic approach. Gen Physiol Biophys 2019; 38: 135-144. doi: 10.4149/gpb_2018042.
189. Sukjoi W, Young C, Acland M, Siritutsoontorn S, Roytrakul S, Klingler-Hoffmann M, Hoffmann P, Jitrapakdee S. Proteomic analysis of holocarboxylase synthetase deficient-MDA-MB-231 breast cancer cells revealed the biochemical changes associated with cell death, impaired growth signaling, and metabolism. Front Mol Biosci 2024: 10: 1250423. doi: 10.3389/fmolb.2023.1250423.
190. Sun R, Ge W, Zhu Y, Sayad A, Luna A, Lyu M, Liang S, Tobalina L, Rajapakse VN, Yu C, Zhang H, Fang J, Wu F, Xie H, Saez-Rodriguez J, Ying H, Reinhold WC, Sander C, Pommier Y, Neel BG, Aebersold R, Guo T. Proteomic Dynamics of Breast Cancer Cell Lines Identifies

Potential Therapeutic Protein Targets. Mol Cell Proteomics 2023; 22: 100602. doi: 10.1016/j.mcpro.2023.100602.
191. Sun X, Lu Z, Liang Z, Deng B, Zhu Y, Shi J, Lu X. Transcriptomics and Proteomics Characterizing the Anticancer Mechanisms of Natural Rebeccamycin Analog Loonamycin in Breast Cancer Cells. Molecules 2022; 27: 6958. doi: 10.3390/molecules27206958.
192. Tang Y, Mackey J, Lai R, Ghosh S, Santos C, Graham K, Damaraju S, Pasdar M, Li L. Quantitative proteomic analysis of HER2 normal and overexpressing MCF-7 breast cancer cells revealed proteomic changes accompanied with HER2 gene amplification. J Proteomics 2013: 91: 200-209. doi: 10.1016/j.jprot.2013.06.034.
193. Tapia IJ, Perico D, Wolos VJ, Villaverde MS, Abrigo M, Di Silvestre D, Mauri P, De Palma A, Fiszman GL.Proteomic Characterization of a 3D HER2+ Breast Cancer Model Reveals the Role of Mitochondrial Complex I in Acquired Resistance to Trastuzumab. Int J Mol Sci 2024; 25: 7397. doi: 10.3390/ijms25137397.
194. Taverna E, De Bortoli M, Maffioli E, Corno C, Ciusani E, Trivulzio S, Pinelli A, Tedeschi G, Perego P, Bongarzone I. Alterations of RNA Metabolism by Proteomic Analysis of Breast Cancer Cells Exposed to Marycin: A New Optically Active Porphyrin. Curr Mol Pharmacol 2019; 12: 147-159. doi: 10.2174/1874467212666190204102112.
195. Tenga MJ, Lazar IM. Proteomic snapshot of breast cancer cell cycle: G1/S transition point. Proteomics 2013; 13: 48-60.doi: 10.1002/pmic.201200188.
196. Tenga MJ, Lazar IM. Proteomic study reveals a functional network of cancer markers in the G1-Stage of the breast cancer cell cycle. BMC Cancer 2014: 14: 710. doi: 10.1186/1471-2407-14-710.
197. Thakur C, Carruthers NJ, Zhang Q, Xu L, Fu Y, Bi Z, Qiu Y, Zhang W, Wadgaonkar P, Almutairy B, Guo C, Stemmer PM, Chen F. Depletion of *Mdig* Changes Proteomic Profiling in Triple Negative Breast Cancer Cells. Biomedicines 2022; 10: 2021. doi: 10.3390/biomedicines10082021.
198. Thuault S, Mamelonet C, Salameh J, Ostacolo K, Chanez B, Salaün D, Baudelet E, Audebert S, Camoin L, Badache A. A proximity-labeling proteomic approach to investigate invadopodia molecular landscape in breast cancer cells. Sci Rep 2020; 10: 6787. doi: 10.1038/s41598-020-63926-4.
199. Tilghman SL, Townley I, Zhong Q, Carriere PP, Zou J, Llopis SD, Preyan LC, Williams CC, Skripnikova E, Bratton MR, Zhang Q, Wang G. Proteomic signatures of acquired letrozole resistance in breast cancer: suppressed estrogen signaling and increased cell motility and invasiveness. Mol Cell Proteomics 2013; 12: 2440-2455. doi: 10.1074/mcp.M112.023861.
200. Toillon RA, Lagadec C, Page A, Chopin V, Sautière PE, Ricort JM, Lemoine J, Zhang M, Hondermarck H, Le Bourhis X.Proteomics demonstration that normal breast epithelial cells can induce apoptosis of breast cancer cells through insulin-like growth factor-binding protein-3 and maspin. Mol Cell Proteomics 2007; 6: 1239-1247. doi: 10.1074/mcp.M600477-MCP200.
201. Torun V, Degerli E, Cansaran-Duman D.Revealing the Molecular Signatures of miR-185-5p on Breast Cancer Cells Using Proteomic. Protein Pept Lett 2024 Sep 25. doi: 10.2174/0109298665322427240906060626.
202. Uppala PT, Dissmore T, Lau BH, Andacht T, Rajaram S. Selective inhibition of cell proliferation by lycopene in MCF-7 breast cancer cells in vitro: a proteomic analysis. Phytother Res 2013; 27: 595-601.doi: 10.1002/ptr.4764.
203. Valle A, Sastre-Serra J, Pol C, Miró AM, Oliver J, Roca P. Proteomic analysis of MCF-7 breast cancer cell line exposed to leptin. Anal Cell Pathol (Amst) 2011; 34: 147-157.doi: 10.3233/ACP-2011-013.
204. Vasilescu J, Smith JC, Ethier M, Figeys D. Proteomic analysis of ubiquitinated proteins from human MCF-7 breast cancer cells by immunoaffinity purification and mass spectrometry. J Proteome Res 2005; 4: 2192-2200.doi: 10.1021/pr050265i.

205. Vasiliou SK, Filippou PS, Clotet-Freixas S, Soosaipillai A, Batruch I, Viktor Tsianos F, Konvalinka A, Diamandis EP. Transcriptome profiling and proteomic validation reveals targets of the androgen receptor signaling in the BT-474 breast cancer cell line. Clin Proteomics 2022 May 14;19(1):14. doi: 10.1186/s12014-022-09352-2.
206. Vercoutter-Edouart AS, Lemoine J, Le Bourhis X, Louis H, Boilly B, Nurcombe V, Révillion F, Peyrat JP, Hondermarck H. Proteomic analysisreveals that 14-3-3sigma is down-regulated in human breast cancer cells. Cancer Res 2001; 61: 76-80.
207. Vercoutter-Edouart AS, Czeszak X, Crépin M, Lemoine J, Boilly B, Le Bourhis X, Peyrat JP, Hondermarck H.Proteomic detection of changes in protein synthesis induced by fibroblast growth factor-2 in MCF-7 human breast cancer cells. Exp Cell Res 2001; 262: 59-68. doi: 10.1006/excr.2000.506
208. Vergara D, Simeone P, Latorre D, Cascione F, Leporatti S, Trerotola M, Giudetti AM, Capobianco L, Lunetti P, Rizzello A, Rinaldi R, Alberti S, Maffia M.Proteomics analysis of E-cadherin knockdown in epithelial breast cancer cells. J Biotechnol 2015: 202: 3-11.doi: 10.1016/j.jbiotec.2014.10.034.
209. Wang K, Shan Z, Duan L, Gong T, Liu F, Zhang Y, Wang Z, Shen J, Lei L. iTRAQ-based quantitative proteomic analysis of Yamanaka factors reprogrammed breast cancer cells. Oncotarget 2017; 8: 34330-34339. doi: 10.18632/oncotarget.16125.
210. Wang W, Lei B, Li L, Liu J, Li Z, Pang Y, Liu T, Li Z. Single-Cell Proteomic Profiling Identifies Nanoparticle Enhanced Therapy for Triple Negative Breast Cancer Stem Cells. Cells 2021; 10: 2842. doi: 10.3390/cells10112842.
211. Wang Y, He QY, Chen H, Chiu JF.Synergistic effects of retinoic acid and tamoxifen on human breast cancer cells: proteomic characterization. Exp Cell Res 2007; 313: 357-68. doi: 10.1016/j.yexcr.2006.10.016.
212. Wang Z, Liang S, Lian X, Liu L, Zhao S, Xuan Q, Guo L, Liu H, Yang Y, Dong T, Liu Y, Liu Z, Zhang Q.Identification of proteins responsible for adriamycin resistance in breast cancer cells using proteomics analysis. Sci Rep 2015: 5: 9301. doi: 10.1038/srep09301.
213. Wei C, Mao A, Liu Y, Zhang Q, Pan G, Liu W, Liu J.Proteomics Analysis of Polyphyllin D-Treated Triple-Negative Breast Cancer Cells Reveal the Anticancer Mechanisms of Polyphyllin D. Appl Biochem Biotechnol 2024; 196: 3148-3161. doi: 10.1007/s12010-023-04679-4.
214. Whelan SA, He J, Lu M, Souda P, Saxton RE, Faull KF, Whitelegge JP, Chang HR. Mass spectrometry (LC-MS/MS) identified proteomic biosignatures of breast cancer in proximal fluid. J Proteome Res 2012; 11: 5034-5045.doi: 10.1021/pr300606e.
215. Witt AE, Hines LM, Collins NL, Hu Y, Gunawardane RN, Moreira D, Raphael J, Jepson D, Koundinya M, Rolfs A, Taron B, Isakoff SJ, Brugge JS, LaBaer J.Functional proteomics approach to investigate the biological activities of cDNAs implicated in breast cancer. J Proteome Res 2006; 5: 599-610.doi: 10.1021/pr050395r.
216. Wu SL, Hancock WS, Goodrich GG, Kunitake ST. An approach to the proteomic analysis of a breast cancer cell line (SKBR-3). Proteomics 2003; 3: 1037-1046.doi: 10.1002/pmic.200300382.
217. Wyrebska A, Pawlowska Z, Gach K, Komorowski P, Protas A, Walkowiak B, Janecka A. Proteomic analysis of proteins engaged in α-methylene-δ-lactone cytotoxic effects in hormone-independent breast cancer MDA-MB-231 cells. Chem Biol Drug Des 2014; 84: 300-306. doi: 10.1111/cbdd.12317.
218. Xia S, Li X, Xu S, Ni X, Zhan W, Zhou W. Sublethal heat treatment promotes breast cancer metastasis and its molecular mechanism revealed by quantitative proteomic analysis. Aging (Albany NY) 2022; 14: 1389-1406. doi: 10.18632/aging.203884.
219. Xiang S, Zhu L, Zhang Z, Wang S, Cui R, Xiang M. Proteomic analysis of inhibitor of apoptosis protein-like protein-2 on breast cancer cell proliferation. Mol Med Rep 2022; 25: 89. doi: 10.3892/mmr.2022.12605.
220. Xu F, Yang T, Fang D, Xu Q, Chen Y. An investigation of heat shock protein 27 and P-glycoprotein mediated multi-drug resistance in breast cancer using liquid chromatography-

tandem mass spectrometry-based targeted proteomics. J Proteomics 2014: 108: 188-197. doi: 10.1016/j.jprot.2014.05.016.
221. Xu SG, Yan PJ, Shao ZM. Differential proteomic analysis of a highly metastatic variant of human breast cancer cells using two-dimensional differential gel electrophoresis. J Cancer Res Clin Oncol 2010; 136: 1545-1556.doi: 10.1007/s00432-010-0812-0.
222. Xu X, Qiao M, Zhang Y, Jiang Y, Wei P, Yao J, Gu B, Wang Y, Lu J, Wang Z, Tang Z, Sun Y, Wu W, Shi Q.Quantitative proteomics study of breast cancer cell lines isolated from a single patient: discovery of TIMM17A as a marker for breast cancer. Proteomics 2010; 10: 1374-90. doi: 10.1002/pmic.200900380.
223. Xu X, Zhang J, Zhang Z, Wang M, Liu Y, Li X.Systems pharmacology in combination with proteomics reveals underlying mechanisms of Xihuang pill against triple-negative breast cancer. Bioengineered 2020; 11: 1170-1188. doi: 10.1080/21655979.2020.1834726.
224. Yan GR, Xu SH, Tan ZL, Liu L, He QY. Global identification of miR-373-regulated genes in breast cancer by quantitative proteomics. Proteomics 2011; 11: 912-920.doi: 10.1002/pmic.201000539.
225. Yang T, Xu F, Xu J, Fang D, Yu Y, Chen Y. Comparison of liquid chromatography-tandem mass spectrometry-based targeted proteomics and conventional analytical methods for the determination of P-glycoprotein in human breast cancer cells. J Chromatogr B Analyt Technol Biomed Life Sci 2013: 936: 18-24. doi: 10.1016/j.jchromb.2013.07.023.
226. Yang Y, Chaerkady R, Beer MA, Mendell JT, Pandey A. Identification of miR-21 targets in breast cancer cells using a quantitative proteomic approach. Proteomics 2009; 9: 1374-1384.doi: 10.1002/pmic.200800551.
227. Yao Z, Li J, Liu Z, Zheng L, Fan N, Zhang Y, Jia N, Lv J, Liu N, Zhu X, Du J, Lv C, Xie F, Liu Y, Wang X, Fei Z, Gao C.Integrative bioinformatics and proteomics-based discovery of an eEF2K inhibitor (cefatrizine) with ER stress modulation in breast cancer cells. Mol Biosyst 2016; 12: 729-736. doi: 10.1039/c5mb00848d.
228. Ye X, Zhang Y, He B, Meng Y, Li Y, Gao Y. Quantitative proteomic analysis identifies new effectors of FOXM1 involved in breast cancer cell migration. Int J Clin Exp Pathol 2015; 8: 15836-44.
229. Yerlikaya A, Okur E, Baykal AT, Acılan C, Boyacı I, Ulukaya E. A proteomic analysis of p53-independent induction of apoptosis by bortezomib in 4T1 breast cancer cell line. J Proteomics 2015: 113: 315-325. doi: 10.1016/j.jprot.2014.09.010.
230. Ying Y, Bian L, Meng Y, Zhang M, Yao Y, Bo F, Li D. Comparative Proteomic Analysis of Irradiation-Induced Radioresistant Breast Cancer Cells Using Label-Free Quantitation. Front Biosci (Landmark Ed) 2023; 28: 244. doi: 10.31083/j.fbl2810244.
231. Zaripov EA, Khraibah A, Kasyanchyk P, Radchanka A, Hüttmann N, Berezovski MV. CE-MS Metabolomic and LC-MS Proteomic Analyses of Breast Cancer Exosomes Reveal Alterations in Purine and Carnitine Metabolism.J Proteome Res 2025. doi: 10.1021/acs.jproteome.4c00795.
232. Zarrineh M, Ashrafian S, Jensen P, Nawrocki A, Ansari AM, Rezadoost H, Ghassempour A, Larsen MR. Comprehensive proteomics and sialiomics of the anti-proliferative activity of safranal on triple negative MDA-MB-231 breast cancer cell lines. J Proteomics 2022: 259: 104539. doi: 10.1016/j.jprot.2022.104539.
233. Zembroski AS, Andolino C, Buhman KK, Teegarden D. Proteomic Characterization of Cytoplasmic Lipid Droplets in Human Metastatic Breast Cancer Cells. Front Oncol 2021: 11: 576326. doi: 10.3389/fonc.2021.576326.
234. Zeng Q, Zhang P, Wu Z, Xue P, Lu D, Ye Z, Zhang X, Huang Z, Feng J, Song L, Yang D, Jiang T, Yan X. Quantitative proteomics reveals ER-α involvement in CD146-induced epithelial-mesenchymal transition in breast cancer cells. J Proteomics 2014: 103:153-169. doi: 10.1016/j.jprot.2014.03.033.
235. Zhang EY, Cristofanilli M, Robertson F, Reuben JM, Mu Z, Beavis RC, Im H, Snyder M, Hofree M, Ideker T, Omenn GS, Fanayan S, Jeong SK, Paik YK, Zhang AF, Wu SL, Hancock WS.

Genome wide proteomics of ERBB2 and EGFR and other oncogenic pathways in inflammatory breast cancer. J Proteome Res 2013; 12: 2805-2817. doi: 10.1021/pr4001527.
236. Zheng G, Peng F, Ding R, Yu Y, Ouyang Y, Chen Z, Xiao Z, He Z.Identification of proteins responsible for the multiple drug resistance in 5-fluorouracil-induced breast cancer cell using proteomics analysis. J Cancer Res Clin Oncol 2010; 136: 1477-1488. doi: 10.1007/s00432-010-0805-z.
237. Zhang H, Angelopoulos N, Xu Y, Grothey A, Nunes J, Stebbing J, Giamas G. Proteomic profile of KSR1-regulated signalling in response to genotoxic agents in breast cancer. Breast Cancer Res Treat 2015; 151: 555-568. doi: 10.1007/s10549-015-3443-y.
238. Zhou C, Nitschke AM, Xiong W, Zhang Q, Tang Y, Bloch M, Elliott S, Zhu Y, Bazzone L, Yu D, Weldon CB, Schiff R, McLachlan JA, Beckman BS, Wiese TE, Nephew KP, Shan B, Burow ME, Wang G. Proteomic analysis of tumor necrosis factor-alpha resistant human breast cancer cells reveals a MEK5/Erk5-mediated epithelial-mesenchymal transition phenotype. Breast Cancer Res2008;10(6):R105.doi: 10.1186/bcr2210.
239. Zhou Q, Chaerkady R, Shaw PG, Kensler TW, Pandey A, Davidson NE. Screening for therapeutic targets of vorinostat by SILAC-based proteomic analysis in human breast cancer cells. Proteomics 2010; 10: 1029-1039.doi: 10.1002/pmic.200900602.
240. Zhou Z, Sicairos B, Zhou J, Du Y. Proteomic Analysis Reveals Major Proteins and Pathways That Mediate the Effect of 17-β-Estradiol in Cell Division and Apoptosis in Breast Cancer MCF7 Cells.J Proteome Res 2024; 23: 4835-4848. doi: 10.1021/acs.jproteome.4c00102.
241. Zhu Z, Boobis AR, Edwards RJ. Identification of estrogen-responsive proteins in MCF-7 human breast cancer cells using label-free quantitative proteomics. Proteomics 2008;8: 1987-2005.doi: 10.1002/pmic.200700901.
242. Ziegler YS, Moresco JJ, Tu PG, Yates JR 3rd, Nardulli AM. Plasma membrane proteomics of human breast cancer cell lines identifies potential targets for breast cancer diagnosis and treatment. PLoS One 2014; 9: e102341.doi: 10.1371/journal.pone.0102341.
243. Ziegler YS, Moresco JJ, Tu PG, Yates JR 3rd, Nardulli AM.Proteomic analysis identifies highly expressed plasma membrane proteins for detection and therapeutic targeting of specific breast cancer subtypes. Clin Proteomics 2018: 15:30. doi: 10.1186/s12014-018-9206-0.

Supplementary File S2: List of In Silico Studies

1. Ahn J, Yoon Y, Yeu Y, Lee H, Park S. Impact of TGF-b on breast cancer from a quantitative proteomic analysis. Comput Biol Med 2013; 43: 2096-2102. doi: 10.1016/j.compbiomed.2013.09.022.
2. Borcherding N, Cole K, Kluz P, Jorgensen M, Kolb R, Bellizzi A, Zhang W. Re-Evaluating E-Cadherin and β-Catenin: A Pan-Cancer Proteomic Approach with an Emphasis on Breast Cancer. Am J Pathol 2018; 188: 1910-1920. doi: 10.1016/j.ajpath.2018.05.003.
3. Collins KAL, Stuhlmiller TJ, Zawistowski JS, East MP, Pham TT, Hall CR, Goulet DR, Bevill SM, Angus SP, Velarde SH, Sciaky N, Oprea TI, Graves LM, Johnson GL, Gomez SM. Proteomic analysis defines kinase taxonomies specific for subtypes of breast cancer. Oncotarget 2018; 9: 15480-15497. doi: 10.18632/oncotarget.24337.
4. Cox TR, Schoof EM, Gartland A, Erler JT, Linding R. Dataset for the proteomic inventory and quantitative analysis of the breast cancer hypoxic secretome associated with osteotropism. Data Brief 2015: 5: 621-5. doi: 10.1016/j.dib.2015.09.039.
5. Fu-Jun L, Shao-Hua J, Xiao-Fang S. Differential proteomic analysis of pathway biomarkers in human breast cancer by integrated bioinformatics. Oncol Lett 2012; 4: 1097-1103. doi: 10.3892/ol.2012.881.
6. Hacking S, Chou C, Baykara Y, Wang Y, Uzun A, Gamsiz Uzun ED. MMR Deficiency Defines Distinct Molecular Subtype of Breast Cancer with Histone Proteomic Networks. Int J Mol Sci 2023; 24: 5327. doi: 10.3390/ijms24065327
7. Hari PS, Balakrishnan L, Kotyada C, Everad John A, Tiwary S, Shah N, Sirdeshmukh R. Proteogenomic Analysis of Breast Cancer Transcriptomic and Proteomic Data, Using De Novo Transcript Assembly: Genome-Wide Identification of Novel Peptides and Clinical Implications. Mol Cell Proteomics 2022; 21: 100220. doi: 10.1016/j.mcpro.2022.100220.
8. Jia G, Yang Y, Ping J, Xu S, Liu L, Guo X, Tao R, Long J, Zheng W. Identification of target proteins for breast cancer genetic risk loci and blood risk biomarkers in a large study by integrating genomic and proteomic data. Int J Cancer 2023; 152: 2314-2320. doi: 10.1002/ijc.34472.
9. Jézéquel P, Guette C, Lasla H, Gouraud W, Boissard A, Guérin-Charbonnel C, Campone M. iTRAQ-Based Quantitative Proteomic Analysis Strengthens Transcriptomic Subtyping of Triple-Negative Breast Cancer Tumors. Proteomics 2019; 19: e1800484. doi: 10.1002/pmic.201800484.
10. Kim SS, Shin H, Ahn KG, Park YM, Kwon MC, Lim JM, Oh EK, Kim Y, Han SM, Noh DY. Quantifiable peptide library bridges the gap for proteomics based biomarker discovery and validation on breast cancer. Sci Rep 2023; 13: 8991. doi: 10.1038/s41598-023-36159-4.
11. Kim TR, Jeong HH, Sohn KA. Topological integration of RPPA proteomic data with multi-omics data for survival prediction in breast cancer via pathway activity inference. BMC Med Genomics 2019; 12:94. doi: 10.1186/s12920-019-0511-x.
12. Lawrence RT, Perez EM, Hernández D, Miller CP, Haas KM, Irie HY, Lee SI, Blau CA, Villén J. The proteomic landscape of triple-negative breast cancer. Cell Rep 2015; 11: 630-644. doi: 10.1016/j.celrep.2015.03.050.
13. Li CL, Moi SH, Lin HS, Hou MF, Chen FM, Shih SL, Kan JY, Kao CN, Wu YC, Kao LC, Chen YH, Lee YC, Chiang CP.Comprehensive Transcriptomic and Proteomic Analyses Identify a Candidate Gene Set in Cross-Resistance for Endocrine Therapy in Breast Cancer. Int J Mol Sci 2022; 23: 10539. doi: 10.3390/ijms231810539.
14. Muraoka S, Kume H, Adachi J, Shiromizu T, Watanabe S, Masuda T, Ishihama Y, Tomonaga T. In-depth membrane proteomic study of breast cancer tissues for the generation of a chromosome-based protein list. J Proteome Res 2013; 12: 208-213. doi: 10.1021/pr300824m.
15. Ősz Á, Lánczky A, Győrffy B. Survival analysis in breast cancer using proteomic data from four independent datasets. Sci Rep 2021; 11: 16787. doi: 10.1038/s41598-021-96340-5.

16. Ramkumar C, Buturovic L, Malpani S, Kumar Attuluri A, Basavaraj C, Prakash C, Madhav L, Doval DC, Mehta A, Bakre MM. Development of a Novel Proteomic Risk-Classifier for Prognostication of Patients With Early-Stage Hormone Receptor-Positive Breast Cancer. Biomark Insights 2018: 13: 1177271918789100. doi: 10.1177/1177271918789100.
17. Ren J, Wang B, Li J.Integrating proteomic and phosphoproteomic data for pathway analysis in breast cancer. BMC Syst Biol 2018; 12: 130. doi: 10.1186/s12918-018-0646-y.
18. Song J, Yang H. Identifying new biomarkers and potential therapeutic targets for breast cancer through the integration of human plasma proteomics: a Mendelian randomization study and colocalization analysis. Front Endocrinol (Lausanne) 2024: 15: 1449668. doi: 10.3389/fendo.2024.1449668.
19. Tobiasz J, Polanska J.Proteomic Profile Distinguishes New Subpopulations of Breast Cancer Patients with Different Survival Outcomes. Cancers (Basel) 2023; 15: 4230. doi: 10.3390/cancers15174230.
20. Wang Y, Yi K, Chen B, Zhang B, Jidong G. Elucidating the susceptibility to breast cancer: an in-depth proteomic and transcriptomic investigation into novel potential plasma protein biomarkers. Front Mol Biosci 2024: 10: 1340917. doi: 10.3389/fmolb.2023.1340917.
21. Yousefian Naeini Z, Esfandiari N, Hashemi M, Hushmandi K, Arbabian S, Entezari M. Introduced the ITGB1-DT as a novel biomarker associated with five potential drugs using bioinformatics analysis of breast cancer proteomics data and RT-PCR. Mol Cell Probes 2023: 71: 101930. doi: 10.1016/j.mcp.2023.101930.
22. Yu Y, Dong L, Dong C, Zhang X. Validation of a Proteomic-Based Prognostic Model for Breast Cancer and Immunological Analysis. Int J Genomics 2023: 2023: 1738750. doi: 10.1155/2023/1738750.
23. Zatorski N, Sun Y, Elmas A, Dallago C, Karl T, Stein D, Rost B, Huang KL, Walsh M, Schlessinger A. Structural analysis of genomic and proteomic signatures reveal dynamic expression of intrinsically disordered regions in breast cancer. iScience 2024; 27: 110640. doi: 10.1016/j.isci.2024.110640.

Supplementary File S3: List of Reviews

1. Aslebagh R, Channaveerappa D, Pentecost BT, Arcaro KF, Darie CC. Combinatorial Electrophoresis and Mass Spectrometry-Based Proteomics in Breast Milk for Breast Cancer Biomarker Discovery. Adv Exp Med Biol 2019: 1140: 451-467. doi: 10.1007/978-3-030-15950-4_26.
2. Behera RN, Bisht VS, Giri K, Ambatipudi K. Realm of proteomics in breast cancer management and drug repurposing to alleviate intricacies of treatment. Proteomics Clin Appl 2023; 17: e2300016. doi: 10.1002/prca.202300016.
3. Blucher AS, Mills GB, Tsang YH. Which path to follow? Utilizing proteomics to improve therapy choices for breast cancer patients. Expert Rev Proteomics 2020; 17: 187-190. doi: 10.1080/14789450.2020.1757442.
4. Brožová K, Hantusch B, Kenner L, Kratochwill K. Spatial Proteomics for the Molecular Characterization of Breast Cancer. Proteomes 2023; 11: 17. doi: 10.3390/proteomes11020017.
5. Celis JE, Gromov P, Gromova I, Moreira JM, Cabezón T, Ambartsumian N, Grigorian M, Lukanidin E, Thor Straten P, Guldberg P, Bartkova J, Bartek J, Lukas J, Lukas C, Lykkesfeldt A, Jäättelä M, Roepstorff P, Bolund L, Ørntoft T, Brünner N, Overgaard J, Sandelin K, Blichert-Toft M, Mouridsen H, Rank FE. Integrating proteomic and functional genomic technologies in discovery-driven translational breast cancer research. Mol Cell Proteomics 2003; 2: 369-377. doi: 10.1074/mcp.R300007-MCP200.
6. Chatterji S, Krzoska E, Thoroughgood CW, Saganty J, Liu P, Elsberger B, Abu-Eid R, Speirs V. Defining genomic, transcriptomic, proteomic, epigenetic, and phenotypic biomarkers with prognostic capability in male breast cancer: a systematic review. Lancet Oncol 2023; 24: e74-e85. doi: 10.1016/S1470-2045(22)00633-7.
7. Chung L, Baxter RC.Breast cancer biomarkers: proteomic discovery and translation to clinically relevant assays. Expert Rev Proteomics 2012; 9: 599-614. doi: 10.1586/epr.12.62.
8. Cowherd SM, Espina VA, Petricoin EF 3rd, Liotta LA. Proteomic analysis of human breast cancer tissue with laser-capture microdissection and reverse-phase protein microarrays. Clin Breast Cancer 2004; 5: 385-392.doi: 10.3816/cbc.2004.n.046.
9. Gromov P, Moreira JM, Gromova I. Proteomic analysis of tissue samples in translational breast cancer research. Expert Rev Proteomics 2014; 11: 285-302. doi: 10.1586/14789450.2014.899469.
10. Guerin M, Gonçalves A, Toiron Y, Baudelet E, Audebert S, Boyer JB, Borg JP, Camoin L.How may targeted proteomics complement genomic data in breast cancer? Expert Rev Proteomics 2017; 14: 43-54. doi: 10.1080/14789450.2017.1256776.
11. Kizhakkeppurath Kumaran A, Sahu A, Singh A, Aynikkattil Ravindran N, Sekhar Chatterjee N, Mathew S, Verma S. Proteoglycans in breast cancer, identification and characterization by LC-MS/MS assisted proteomics approach: A review. Proteomics Clin Appl 2023; 17: e2200046. doi: 10.1002/prca.202200046.
12. Lam SW, Jimenez CR, Boven E. Breast cancer classification by proteomic technologies: current state of knowledge. Cancer Treat Rev 2014; 40: 129-138. doi: 10.1016/j.ctrv.2013.06.006.
13. Laronga C, Drake RR.Proteomic approach to breast cancer. Cancer Control 2007; 14: 360-368.doi: 10.1177/107327480701400406.
14. Li Y, Kong X, Wang Z, Xuan L. Recent advances of transcriptomics and proteomics in triple-negative breast cancer prognosis assessment. J Cell Mol Med 2022; 26: 1351-1362. doi: 10.1111/jcmm.17124.
15. Lumachi F, Chiara GB, Foltran L, Basso SM.Proteomics as a Guide for Personalized Adjuvant Chemotherapy in Patients with Early Breast Cancer. Cancer Genomics Proteomics 2015; 12: 385-390.
16. Mardamshina M, Geiger T. Next-Generation Proteomics and Its Application to Clinical Breast Cancer Research. Am J Pathol 2017; 187: 2175-2184. doi: 10.1016/j.ajpath.2017.07.003.

17. Mazur MG, Pyatchanina TV. The use of proteomic technologies in breast cancer research. Exp Oncol 2016; 38: 146-57.
18. Metwali E, Pennington S. Mass Spectrometry-Based Proteomics for Classification and Treatment Optimisation of Triple Negative Breast Cancer. J Pers Med 2024; 14: 944. doi: 10.3390/jpm14090944.
19. Miah S, Banks CA, Adams MK, Florens L, Lukong KE, Washburn MP. Advancement of mass spectrometry-based proteomics technologies to explore triple negative breast cancer. Mol Biosyst 2016; 13: 42-55. doi: 10.1039/c6mb00639f.
20. Mouridsen HT, Brünner N. Clinical infrastructures to support proteomic studies of tissue and fluids in breast cancer. Mol Cell Proteomics 2004; 3: 302-310.doi: 10.1074/mcp.R400003-MCP200.
21. Nalla LV, Kanukolanu A, Yeduvaka M, Gajula SNR. Advancements in Single-Cell Proteomics and Mass Spectrometry-Based Techniques for Unmasking Cellular Diversity in Triple Negative Breast Cancer. Proteomics Clin Appl 2024: e202400101. doi: 10.1002/prca.202400101.
22. Neagu AN, Jayathirtha M, Whitham D, Mutsengi P, Sullivan I, Petre BA, Darie CC. Proteomics-Based Identification of Dysregulated Proteins in Breast Cancer. Proteomes 2022; 10: 35. doi: 10.3390/proteomes10040035.
23. Neagu AN, Whitham D, Buonanno E, Jenkins A, Stratulat TA, Tamba BI, Darie CC. Proteomics and its applications in breast cancer. Am J Cancer Res 2021; 11: 4006-4049. eCollection 2021.
24. Neagu AN, Whitham D, Seymour L, Haaker N, Pelkey I, Darie CC. Proteomics-Based Identification of Dysregulated Proteins and Biomarker Discovery in Invasive Ductal Carcinoma, the Most Common Breast Cancer Subtype. Review Proteomes 2023; 11: 13. doi: 10.3390/proteomes11020013.
25. Neubauer H, Fehm T, Schütz C, Speer R, Solomayer E, Schrattenholz A, Cahill MA, Kurek R. Proteomic expression profiling of breast cancer. Recent Results Cancer Res2007:176:89-120.doi: 10.1007/978-3-540-46091-6_9.
26. Ruhlen RL, Sauter ER.Proteomic analysis of breast tissue and nipple aspirate fluid for breast cancer detection. Biomark Med 2007; 1: 251-260.doi: 10.2217/17520363.1.2.251.
27. van der Werff MP, Mertens B, de Noo ME, Bladergroen MR, Dalebout HC, Tollenaar RA, Deelder AM. Case-control breast cancer study of MALDI-TOF proteomic mass spectrometry data on serum samples. Stat Appl Genet Mol Biol2008; 7: Article2. doi: 10.2202/1544-6115.1352.
28. Varnum SM, Covington CC, Woodbury RL, Petritis K, Kangas LJ, Abdullah MS, Pounds JG, Smith RD, Zangar RC. Proteomic characterization of nipple aspirate fluid: identification of potential biomarkers of breast cancer. Breast Cancer Res Treat 2003; 80: 87-97.doi: 10.1023/A:1024479106887.
29. Whitham D, Bruno P, Haaker N, Arcaro KF, Pentecost BT, Darie CC. Deciphering a proteomic signature for the early detection of breast cancer from breast milk: the role of quantitative proteomics. Review Expert Rev Proteomics 2024; 21: 81-98. doi: 10.1080/14789450.2024.2320158.
30. Wulfkuhle JD, McLean KC, Paweletz CP, Sgroi DC, Trock BJ, Steeg PS, Petricoin EF 3rd. New approaches to proteomic analysis of breast cancer. Proteomics 2001; 1: 1205-1215. doi: 10.1002/1615-9861(200110)1:10<1205::AID-PROT1205>3.0.CO;2-X.
31. Zhu Z, Jiang L, Ding X. Advancing Breast Cancer Heterogeneity Analysis: Insights from Genomics, Transcriptomics and Proteomics at Bulk and Single-Cell Levels. Review Cancers (Basel) 2023; 15: 4164. doi: 10.3390/cancers15164164.
32. Zografos E, Gazouli M, Tsangaris G, Marinos E. The Significance of Proteomic Biomarkers in Male Breast Cancer. Cancer Genomics Proteomics 2016; 13: 183-190.
